# DNA‐Encoded Libraries: Aryl Fluorosulfonates as Versatile Electrophiles Enabling Facile On‐DNA Suzuki, Sonogashira, and Buchwald Reactions

**DOI:** 10.1002/advs.201901551

**Published:** 2019-09-30

**Authors:** Hongtao Xu, Fei Ma, Nan Wang, Wei Hou, Huan Xiong, Fengping Lu, Jie Li, Shuyue Wang, Peixiang Ma, Guang Yang, Richard A. Lerner

**Affiliations:** ^1^ Shanghai Institute for Advanced Immunochemical Studies ShanghaiTech University Shanghai 201210 China; ^2^ College of Pharmaceutical Science and Institute of Drug Development & Chemical Biology (IDD & CB) Zhejiang University of Technology Hangzhou 310014 China; ^3^ Department of Chemistry Scripps Research Institute La Jolla CA 92037 USA

**Keywords:** Buchwald, DNA‐encoded library, fluorosulfonate, palladium, Sonogashira, Suzuki

## Abstract

Using (hetero)aryl fluorosulfonates as versatile electrophiles, facile on‐DNA cross‐coupling reactions of Suzuki, Sonogashira, and Buchwald are reported here. Notably, all of these reactions show excellent functional group tolerance, mild reaction conditions (relative low temperature and open to air), rich heterocyclic coupling partners, and more importantly, DNA‐compatibility. Thus, these new reactions based on efficient formation of C(sp^2^)‐C(sp^2^), C(sp^2^)‐C(sp), and C(sp^2^)‐N bonds are highly amenable to synthesis of DNA‐encoded libraries with great molecular diversity.

## Graphical abstract

Mild on‐DNA Suzuki, Sonogashira, and Buchwald cross‐coupling reactions use (hetero)aryl fluorosulfonates as versatile electrophiles.

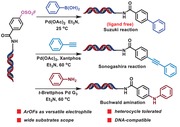

## 1 Introduction

Since the pioneer work by Lerner and Brenner in 1992,^1^ DNA‐encoded library (DEL) technology which combines DNA synthesis, amplification, and sequencing with DNA‐compatible organic reactions has emerged as a powerful platform in small molecule drug discovery.^2^, ^3^, ^4^, ^5^ It enables the screening of ultra‐large compound libraries in an unprecedented high‐throughput and cost‐efficient manner.^6^ Compounds in DELs consist of building blocks (BBs) serving as diversity elements, which are encoded by unique DNA sequences as identification “barcodes,” and are assembled combinatorial using split‐and‐pool strategy or alternative procedures via DNA‐compatible reactions.^7^, ^8^, ^9^ DELs comprising millions or even billions of DNA‐tagged druglike molecules can be effectively synthesized via this technology and screened against various protein targets of interest in a single pooled assay. In a typical affinity‐based selection experiment, when non‐ and low‐affinity binders are washed away, the DNA tag of the remaining compounds can be amplified using polymerase chain reaction and the relative frequency of the remaining compounds before and after selection is determined by counting the number of DNA tags in high‐throughput DNA sequencing experiments.^10^, ^11^ To date, a large number of high‐quality hits have been identified by the DEL technology for various therapeutically relevant targets, such as kinases,^12^ phosphatases,^13^ and G‐protein coupled receptors.^14^ Several drug candidates derived from their corresponding DEL hits, such as soluble epoxide hydrolase inhibitor GSK2256294, and death domain receptor‐associated adaptor kinase RIP1 inhibitor GSK2982772 have progressed to late‐stage clinical development,^15^, ^16^ further emphasizing DEL as a powerful technology for small molecular drug discovery.

**Figure 1:**
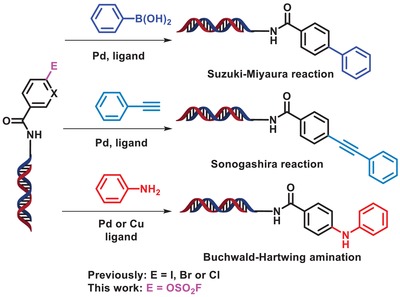
ArOFs‐based on‐DNA Suzuki‐Miyaura, Sonogashira, and Buchwald‐Hartwig cross‐coupling reactions development.

Despite these successes, the great potential of DEL technology in drug discovery has not yet been fully realized. One of the most fundamental challenges is the synthesis of high‐quality libraries with more structural diversity, which in turn depends on the development of new and robust DNA‐compatible reactions that allow more flexibility in DEL's design and synthesis. Among various DNA‐compatible reactions developed in recent years, transition‐metal‐promoted reactions such as Suzuki‐Miyaura coupling,^17^, ^18^, ^19^, ^20^, ^21^, ^22^, ^23^ Sonogashira coupling, and Buchwald‐Hartwig amination using DNA‐conjugated aryl halides as electrophiles have been elegantly developed (**Figure** 1),^24^, ^25^, ^26^ and some of them have been developed for DEL synthesis. However, the environmental toxicity and high costs of aryl halides have hindered their large‐scale applications in industry. Thus, much attention has been paid to phenol‐derived electrophiles recently,^27^ which, as compared to aryl halides, offer a more sustainable starting material because most of them are readily available from biomass.^28^ In addition, phenol modules are also important components of natural products (NPs), bioactive molecules, and pharmaceutical drugs. Coupling reactions with phenol‐derived electrophiles could offer new synthetic strategies for direct or late‐stage DELs.

Phenol‐derived aryl tosylates and mesylates have been successfully applied for various transition‐metal‐catalyzed reactions in spite of the fact that they are still expensive and even exhibit low reactivity.^29^, ^30^ Fluorosulfonates, also known as “super sulfates,” were first reported more than four decades ago,^31^, ^32^, ^33^, ^34^, ^35^, ^36^ their chemistry, however, is relatively unexplored. In 2014, by revisiting the unique properties of fluorosulfonates, the Sharpless group reported a simple and reliable method for the synthesis of aryl fluorosulfonates from phenols and relatively cheaper sulfuryl fluoride (SO_2_F_2_).^37^ Since then numerous investigations of the chemistry of fluorosulfonates have been sparked,^38^, ^39^ especially the transition‐metal‐promoted reactions, such as Suzuki‐Miyaura coupling,^40^, ^41^ and Buchwald‐Hartwig amination using fluorosulfonates as electrophiles.^42^ These works demonstrated that the ‐OSO_2_F group is an atom‐efficient and economically viable alternative to halide and/or triflate.

To further develop fluorosulfonates' chemistry and expand the toolbox of DEL syntheses, our group aimed to develop fluorosulfonates‐based DNA‐compatible coupling reactions. Notably, the following considerations have to be confronted: 1) Due to the high functionalization of DNA,^17^, ^43^ on‐DNA reactions are typically carried out in aqueous media (with > 20% water as co‐solvent) at extremely high dilution (0.1 × 10^–3^−1 × 10^−3^ m for DELs vs 0.1–1 m for conventional chemical reactions) under mild reaction conditions (pH 4–14 and 25–90 °C); 2) the reactions must be robust and amenable to a wide scope of substrates and coupling partners, especially for those containing heterocycles, since heterocycles such as indole, pyridine, etc., are privileged structures in small molecule drugs. We reason that this challenging task could be accomplished by fine‐tuning the catalytic systems. Herein, we describe the development of a general strategy using mild on‐DNA palladium‐promoted Suzuki‐Miyaura, Sonogashira, and Buchwald‐Hartwig cross‐coupling reactions with (hetero)aryl fluorosulfonates (ArOFs) as versatile electrophiles (Figure 1).

## 2 Results

Previous reports showed that sulfuryl fluoride (SO_2_F_2_) is a versatile and powerful reagent that could quantitatively decorate various phenols under mild conditions, even in the presence of water.^44^ Considering the feasibility and safety of practical operation and the characteristic of small reaction scale of on‐DNA synthesis, we chose solid reagents [4‐(acetylamino)phenyl]imidodisulfuryl difluoride (AISF)^45^ and fluorosulfuryl imidazolium salt^46^ that had recently developed by scientists at BioDuro/Pfizer, and the Dong group, respectively, for the on‐DNA synthesis of ArOSO_2_F. The results of comparative trials suggested that AISF was a more suitable reagent for the current on‐DNA ‐SO_2_F decoration (see Table S1, Supporting Information). Following the reaction of DNA‐conjugated 4‐hydroxyl benzoic acid with 100 equivalents of AISF and 200 equivalents of 1,8‐diazabicyclo[5.4.0]undec‐7‐ene (DBU) in borate buffer (pH 9.4) at room temperature for 2 h, the desired DNA‐conjugate aryl fluorosulfonate (HP‐ArOFs‐**1**) was obtained in 96% yield. Under optimized reaction conditions, a series of DNA‐conjugated ArOFs have been obtained in high efficiency (see Schemes S1 and S2, Supporting Information).

**Scheme 1:**
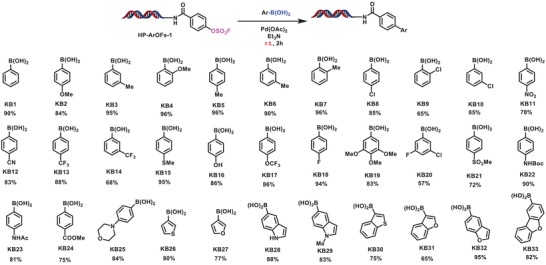
On‐DNA Suzuki‐Miyaura coupling of representative boronic acids with HP‐ArOFs‐**1**. Reaction conditions: 1 equiv of HP‐ArOFs‐**1** (1 × 10^−3^ m in borate buffer), 400 equiv of the relative boronic acid (400 × 10^−3^ m in DMA), 20 equiv of Pd(OAc)_2_ (20 × 10^−3^ m in DMA), 1000 equiv of Et_3_N (5000 × 10^−3^ m in DMA), water, r.t. The conversion of HP‐ArOFs‐**1** was determined by liquid chromatography–mass spectrometry (LC‐MS).

With HP‐ArOFs in hand, in light of the importance of biaryls in medicinal chemistry and the universe of boronic acids, we investigated the on‐DNA potential of ArOFs in Suzuki‐Miyaura cross‐coupling reaction with various boronic acids. Suzuki‐Miyaura coupling is one of the powerful and convenient approach for C(sp^2^)−C(sp^2^) bond formation, as it has the properties of mild reaction conditions, high tolerance toward functional groups, high stability, and wide diversity of commercially available boronic acids.^47^ In recent years, tremendous progresses have been made in conducting Suzuki reactions in water or biphasic water‐organic solvent systems from green chemistry point of views.^48^, ^49^, ^50^ In particular, Jiang's lab reported the palladium‐catalyzed cross‐coupling reaction with aryl boronic acids, which was carried out in water at room temperature with excellent yields.^40^ Inspired by this work, we explored the reaction conditions using HP‐ArOFs‐**1** and boronic acid **KB1** as model substrates, and palladium(II)acetate as catalyst. We evaluated the effect of bases and co‐solvents and found that, in the presence of 20 equivalents of Pd(OAc)_2_, 400 equivalents of boronic acid, and 1000 equivalents of triethylamine (Et_3_N) with *N*,*N*‐dimethylaniline (DMA) as a co‐solvent, the cross‐coupling reaction proceeded smoothly with excellent conversion (90%) at room temperature in a ligand‐free style (see Table S2, Supporting Information). Subsequently, we examined the scope of boronic acids in on‐DNA Suzuki cross‐coupling reaction. As shown in **Scheme** 1, the reaction displayed good reactivity and tolerance to aryl boronic acids with functional groups both electron‐rich (**KB1**, **KB2**, **KB3**, **KB4**, **KB16**, **KB19**, and **KB25**) and electron‐deficient (**KB11**, **KB12**, **KB13**, **KB14**, **KB21**, and **KB24**). Notably, sterically hindered *ortho*‐substituted aryl boronic acids (**KB4**, **KB7**, and **KB9**) afforded good to excellent conversion; some sensitive functional groups such as nitrile (**KB12**), ester (**KB24**), and methylthio (**KB15**) that are labile to hydrolysis or temper catalytic activity of palladium were all well‐tolerated in the reaction;^20^ and nitrogen‐ or sulfur‐containing heteroaryl boronic acids, which are typically difficult to react,^51^, ^52^ could also be efficiently coupled with HP‐ArOFs‐**1** to give good conversion in this context. More importantly, we showed that the chloride‐containing boronic acids (**KB8**, **KB9**, and **KB10**) afforded the desired products with satisfactory conversion, and this is in accordance with the previously reported reactivity profile of common electrophilic leaving groups (‐I > ‐Br > OFs > Cl).^41^, ^53^ To further exploit the potential, it would be of great interest to perform stepwise chemo‐selective on‐DNA synthesis of poly‐substituted aryl and/or heteroaryl molecules,^18^ which are challenging tasks even in conventional organic synthesis.^53^, ^54^ To our delight, the heterocycle‐contained boronic acids such as thiophene, furan, and indole were all well‐tolerated and yielded corresponding products with good to excellent conversion (**KB25‐33**, 65–95%). We next investigated the substrate scope regarding DNA‐conjugated (hetero)aryl sulfonates (see Scheme S3, Supporting Information). The Suzuki reaction proved to be highly efficient for cross‐coupling of the corresponding (hetero)aryl sulfonates, especially of which, DNA‐conjugated (hetero)aryl sulfonates (HP‐ArOFs‐**4,** HP‐ArOFs‐**5**, and HP‐ArOFs‐**8**, 73–86%) were shown to give satisfactory conversion.

**Scheme 2:**
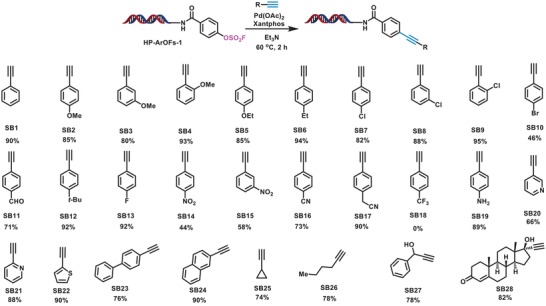
On‐DNA Sonogashira coupling of representative alkynes with HP‐ArOFs‐**1**. Reaction conditions: 1 equiv of HP‐ArOFs‐**1** (1 × 10^−3^ m in water), 200 equiv of 1‐ethynyl‐4‐methoxybenzene (200 × 10^−3^ m in DMA), 20 equiv of Pd(OAc)_2_ (20 × 10^−3^ m in DMA), 40 equiv of Xantphos (40 × 10^−3^ m in DMA), 1000 equiv of base (5000 × 10^−3^ m in DMA), 60 °C, 2 h. Conversion of HP‐ArOFs‐**1** was determined by LC‐MS.

Among various transition‐metal‐catalyzed cross‐coupling reactions, the palladium‐catalyzed C(sp^2^)‐C(sp) coupling reaction between alkynes and aryl or alkenyl halides or triflates, in the presence or absence of a copper (I), is another important method to prepare arylalkynes and/or conjugated enynes, which are key precursors for various NPs and pharmaceuticals.^55^ Previously, only a few pilot experiments reported palladium‐catalyzed cross‐coupling reaction of ArOFs and terminal alkyne with moderate yields in conventional organic synthesis. The use of ArOFs as electrophiles to perform the on‐DNA Sonogashira reaction has yet to be reported.^40^, ^45^ We started our investigation by testing the on‐DNA reaction of HP‐ArOFs‐**1** using phenylacetylene **SB1**. A systemic investigation on the influence of palladium source, ligands, base, and reaction temperature was performed. Results showed that by using 20 equivalents of Pd(OAc)_2_, 40 equivalents of Xantphos (40 × 10^−3^ m in DMA), 1000 equivalents of triethylamine (Et_3_N), the desired cross‐coupling product could be obtained with 85% conversion in a copper‐free fashion (see Table S3, Supporting Information). To determine the substrate scope, we investigated reactions of HP‐ArOFs‐**1** with various terminal alkynes. **Scheme** 2 summarizes the corresponding alkynes that provided the expected C(sp^2^)‐C(sp) coupling products in moderate to excellent conversion. As shown in Scheme 1, both electron‐neutral (**SB1**, **SB6**, and **SB12**) and electron‐rich (**SB2**, **SB3**, **SB4**, **SB5**, **SB19**, and **SB26**) terminal aryl alkynes could perform efficient Sonogashira cross‐coupling reaction with HP‐ArOFs‐**1** in high conversion yields; whereas electron‐deficient (**SB10**, **SB11**, **SB14**, **SB15**, and **SB16**) terminal aryl alkynes generally afforded moderate cross‐coupling yield. Gratifyingly, chloride‐substituted terminal aryl alkynes (**SB7**, **SB8**, and **SB9**) were also found to be efficient coupling partners that generated final coupling products in high yield. The intactness of chloride in **SB7**, **SB8**, and **SB9** during the Sonogashira reaction makes these BBs highly suitable as bifunctional BBs, a feature of great importance in DEL synthesis. Furthermore, we found that nitrogen‐ or sulfur‐containing heteroaryl alkynes (**SB20**, **SB21**, and **SB22**) as well as alkynes bearing functional groups such as cyclopropyl (**SB25**), hydroxyl (**SB27** and **SB28**) could also be efficiently coupled with HP‐ArOFs‐**1** to give good conversion under current reaction conditions. We next investigated the substrate scope regarding DNA‐conjugated terminal (hetero)aryl alkynes (see Scheme S4, Supporting Information). The Sonogashira reaction proved to be highly efficient in cross‐coupling of the corresponding (hetero)aryl sulfonates, especially DNA‐conjugated (hetero)aryl sulfonates (HP‐ArOFs‐**4**, HP‐ArOFs‐**5**, and HP‐ArOFs‐**8**), which could react with phenylacetylene **SB1** to give the corresponding DNA‐conjugated aryl alkynes with moderate to high conversion (50–90%).

**Scheme 3:**
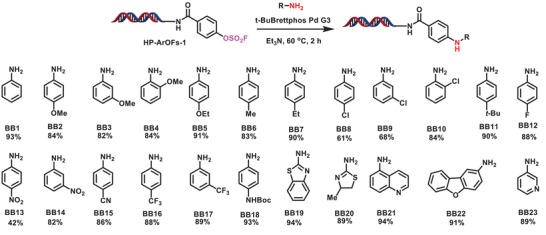
On‐DNA Buchwald amination of representative amines with HP‐ArOFs‐**1**. Reaction conditions: 1 equiv of HP‐ArOFs‐**1** (1 × 10^−3^ m in water), 200 equiv of amine (200 × 10^−3^ m in DMA), 10 equiv of *t*‐Brettphos Pd G3 (10 × 10^−3^ m in DMA), 1000 equiv of base (5000 × 10^−3^ m in DMA), 60 °C, 2 h. The conversion of HP‐ArOFs‐**1** was determined by LC‐MS.

Encouraged by the success of the above Suzuki and Sonogashira cross‐coupling reactions and in consideration of the importance of Buchwald amination reaction in providing medicinal chemistry critical C‐N cross‐coupling products, we next seek to investigate the potential of ArOFs in Csp^2^‐N formation. Actually, C–N cross‐coupling is much more appealing than C–C coupling for DNA‐conjugated aryl halides because amines are important module to adjust the *p*Ka, solubility, as well as permeability of small molecule drugs. We systemically investigated the influence of palladium source, ligands, bases, and reaction temperatures on the cross‐coupling reaction. Results showed that *t*‐Brettphos Pd G3 (10 equiv) was an effective catalyst, combined with 200 equivalents of aniline, 1000 equivalents of Et_3_N, the desired coupling reaction product could afford 93% conversion yield (see Table S4, Supporting Information). Subsequently, to determine the substrate scope, we investigated the Buchwald cross‐coupling reaction of HP‐ArOFs‐**1** with various aryl amines. **Scheme** 3 summarized the tested aryl amines that provided desired C(sp^2^)‐N coupling products in good conversion yields. As shown in Scheme 2, both electron‐rich (**BB2**, **BB3**, **BB4**, and **BB5**) and electron‐deficient (**B16**, **B17**, and **B18**) aryl amines, except for *p*‐nitroaniline (**BB13**), could undergo efficient cross‐coupling reaction with HP‐ArOFs‐**1** in high conversion yields. We found that sterically hindered *ortho*‐substituted aryl amines (**B4** and **B10**) could also give good conversion; functional groups, e.g., nitrile (**BB16**) was highly compatible; chloride‐substituted aryl amines (**BB8**, **BB9**, and **BB10**) were efficient coupling partners that gave the resulting coupling products in high yield. Similar to the above Suzuki and Sonogashira reactions, these results showed great promise of these BBs as bifunctional linkers for DEL synthesis. Again, like in the content of Suzuki and Sonogashira reactions, nitrogen‐ and sulfur‐containing heteroaryl amines (**BB19, BB20**, **BB21**, **BB22**, and **BB23**) were shown to efficiently couple with HP‐ArOFs‐**1** to provide the desired DNA‐conjugated diarylamines, and the substrate scope regarding DNA‐conjugated ArOFs was investigated (see Scheme S5, Supporting Information). Under the current reaction conditions, the Buchwald amination reaction proved to be highly efficient in cross‐coupling of the corresponding ArOFs, especially DNA‐conjugated (hetero)aryl sulfonates (HP‐ArOFs‐**4** and HP‐ArOFs‐**9**), which could react efficiently with aniline **BB1** to give the corresponding DNA‐conjugated diarylamines in moderate to good conversion yields (50–91%).

## 3 Discussion and Conclusion

To realize the full potential of DEL technology, new chemistry that enables greater structural diversity attracts attention of both the academia and pharmaceutical industry. Two general approaches, conventional split‐pool synthesis in the presence of DNA and late‐stage DNA annotation of existing NPs,^56^, ^57^ both require an orthogonal bifunctional chemical linker compatible with aqueous reaction condition and DNAs. We have identified that ArOFs, a versatile electrophile, is a novel reactive agent in palladium‐catalyzed on‐DNA Suzuki, Sonogashira, and Buchwald cross‐coupling reactions. We optimized reaction conditions in terms of function tolerability, DNA compatibility, and heterocyclic coupling partners' diversity. We demonstrated that the formation of C(sp^2^)‐C(sp^2^), C(sp^2^)‐C(sp), and C(sp^2^)‐N bonds in these cross‐coupling reactions were facile at low reaction temperature and under air. The reactivity profile of ‐OFs and other common electrophilic leaving groups (‐I > ‐Br > OFs > Cl) makes it possible to carry out sequential on‐DNA synthesis to form poly‐substituted aryl and/or heteroaryl molecules in DELs. In addition to maximizing the reaction scope and substrates of DNA‐conjugated ArOFs, these new on‐DNA coupling reactions could be applied to modifications of phenol‐containing NPs, bioactive molecules, and pharmaceutical drugs. The method described herein represents one important synthetic strategy in DEL chemistry to surmount the limitation of on‐DNA reactions of many reactive chemical structures.

## Conflict of Interest

The authors declare no conflict of interest.

## Supporting information

Supplementary
