# Supplementary material for: DNA‐Encoded Libraries: Aryl Fluorosulfonates as Versatile Electrophiles Enabling Facile On‐DNA Suzuki, Sonogashira, and Buchwald Reactions
Source: Adv Sci (Weinh). 2019 Sep 30;6(23):1901551. doi: 10.1002/advs.201901551 (PMC6891896; doi:10.1002/advs.201901551)
Supplement: Supplementary file 1 — Supplementary [file ADVS-6-1901551-s001.pdf]

Copyright WILEY-VCH Verlag GmbH & Co. KGaA, 69469 Weinheim, Germany, 2019.

Supporting Information

for *Adv. Sci.,* DOI: 10.1002/advs.201901551

DNA-Encoded Libraries: Aryl Fluorosulfonates as Versatile Electrophiles Enabling Facile On-DNA Suzuki, Sonogashira, and Buchwald Reactions

Hongtao Xu, Fei Ma, Nan Wang, Wei Hou, Huan Xiong, Fengping Lu, Jie Li, Shuyue Wang, Peixiang Ma, Guang Yang, and Richard A. Lerner**

Electronic Supporting Information

DNA encoded libraries: aryl fluorosulfonates as versatile electrophiles enabling facile on-DNA Suzuki, Sonogashira, and Buchwald reactions

Hongtao Xu [a#], Fei Ma [a#], Nan Wang [a], Wei Hou [b], Huan Xiong [a], Fengping Lu [a], Jie Li [a], Shuyue Wang [a], Peixiang Ma [a], Guang Yang [a,*], Richard A. Lerner [c*]

[a]Shanghai Institute for Advanced Immunochemical Studies, ShanghaiTech University, 201210 Shanghai, China
[b]College of Pharmaceutical Science, and Institute of Drug Development & Chemical Biology (IDD & CB), Zhejiang University of Technology, 310014 Hangzhou, China.
[c]Department of Chemistry, Scripps Research Institute, La Jolla, CA 92037

[#] Contributed equally

* To whom correspondence may be addressed.
Email: yangguang@shanghaitech.edu.cn, or rlerner@scripps.edu.

Contents

General Methods..2
Structure of DNA Headpiece 3
Scheme S1. Synthesis of DNA conjugated phenols...4
Table S1. Optimization of reaction conditions for HP-DNA-Aryl fluorosulfonates synthesis[a]..5
Scheme S2. Preparation of DNA conjugated (hetero) aryl fluorosulfonates 5
Table S2. Optimization of on-DNA Suzuki-Miyaura coupling reaction conditions[a] ...6
General Procedures for on-DNA Suzuki-Miyaura Coupling of Representative Boronic Acids and HP-ArOFs-1...7
Scheme S3. Reactions of DNA conjugated (hetero) aryl fluorosulfonates with phenylboronic acid..7
Table S3. Optimization of on-DNA Sonogoshira reaction conditions[a] 9
General Procedures for on-DNA Sonogashira Coupling of Representative Alkynes and HP-ArOFs-1 10
Scheme S4. Reactions of DNA conjugated (hetero) aryl fluorosulfonates with 1-ethynyl-benzene...11
Table S4. Optimization of on-DNA Buchwald reaction conditions[a] 12
General Procedures for on-DNA Buchwald Amination of Representative Amines and HP-ArOFs-1...13
Scheme S5. Reactions of DNA conjugated (hetero) aryl fluorosulfonates with anilines 14
MS Spectra of DNA conjugated phenols 15
MS Spectra of DNA conjugated (hetero)aryl fluorosulfonates.............................20
MS Spectra of on-DNA Suzuki-Miyaura coupling products...............................25
MS Spectra of on-DNA Sonogashira coupling products.......................................46
MS Spectra of on-DNA Buchwald amination products 64

General Methods

All reagents and DNA headpiece HP-NH_2 (5'- / 5phos / GAGTCA / iSp9 / iUniAmM / iSp9 / TGACTCCC-3', Figure 1) were obtained from commercial sources unless

otherwise noted and used as received. All on-DNA reactions were performed in 1.5 mL or 5 mL Eppendorf tubes. On-DNA reactions in the studies of reaction condition optimization and substrate scope extension were analyzed by LC-MS. Typically, samples were dissolved in an appropriate amount of distilled and deionized water (ddH_2O) and injected into a reverse-phase chromatography column (Xbridge Oligonucleotide BEH C18 column, 1.7 μm, 2.1×50 mm). The elution was carried out as followings: 5–95% solvent B over 4.5 min, 0.4 mL/min, λ = 260 nm; solvent A: 0.75% v/v hexafluoroisopropanol/ 0.038% v/v triethylamine in methanol/water = 5/95; solvent B: 0.75% v/v hexafluoroisopropanol/ 0.038% v/v triethylamine in methanol/water = 90/10. The effluents were analyzed by an ABSCIEX 4600 TOF electrospray mass spectrometer in negative ion mode. Mass deconvolution was carried out using Peakview software. Percent conversion for DNA conjugated reactions were based on the areas of TIC peaks of LC-MS.

The Structure of DNA Headpiece

DNA headpiece HP-NH_2(5′/5Phos/GAGTCA/iSp9/iUniAmM/iSp9/TGACTCCC-3′)

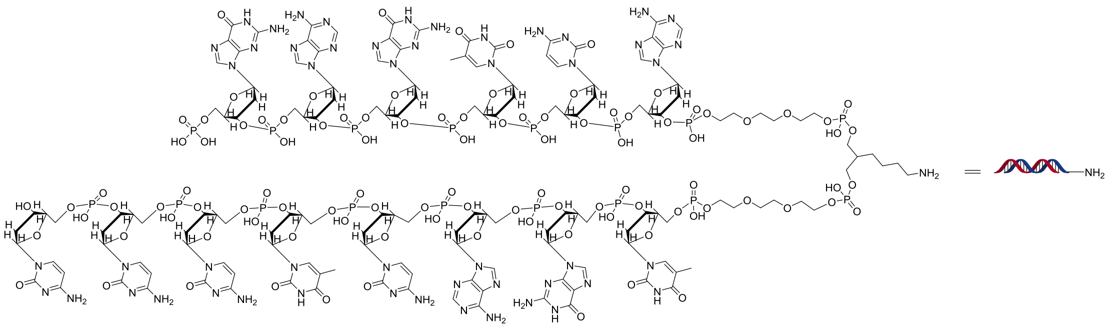

Figure 1. The Structure of DNA Headpiece

Scheme S1. Synthesis of DNA conjugated phenols

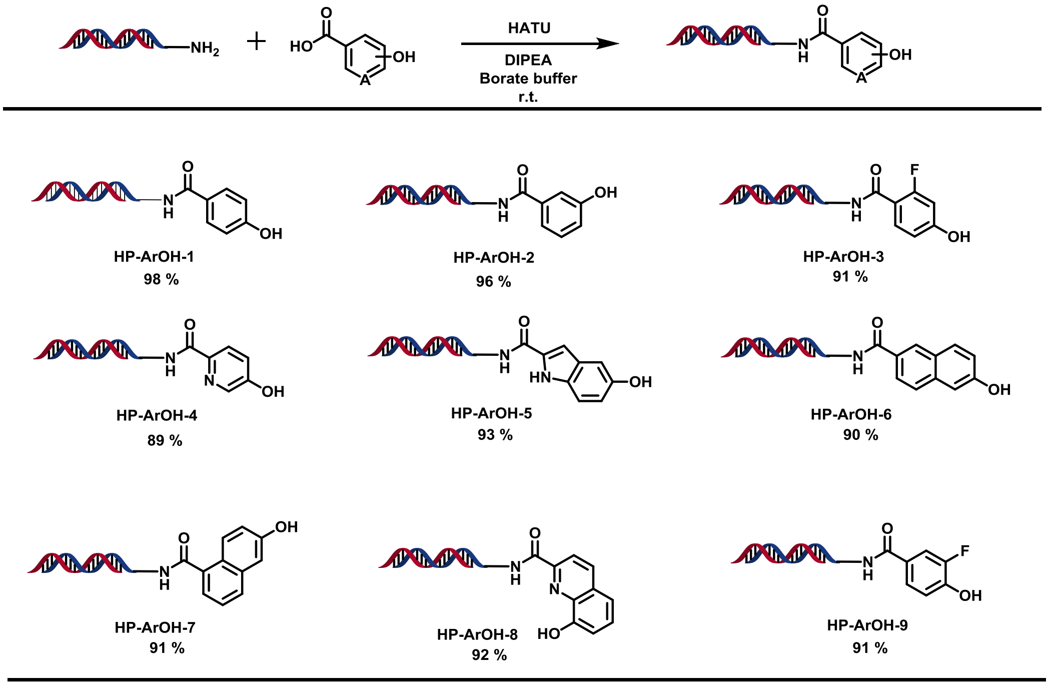

To a solution of DNA headpiece (600 μL, 300 nmol) in borate buffer (250 mM, pH = 9.4), was added a mixture of DMA solution of HATU (60 μL, 200 mM), DIPEA (60 μL, 200 mM) and acids (60 μL, 200 mM). The resultant mixture was vortexed and stood at 25 °C for 8 hours. 5M NaCl (78 μL) and cold ethanol (2.34 mL) were sequentially added, and the resultant mixture was stored at −80 °C for 30 min. The mixture was centrifuged at 4 °C for 30 min at 12000 rpm to remove the supernatant. The resulting pellet was re-dissolved in ddH_2O (300 μL), which was used in following reaction without further purification.

Table S1. Optimization of reaction conditions for HP-DNA-Aryl fluorosulfonates synthesis[a]

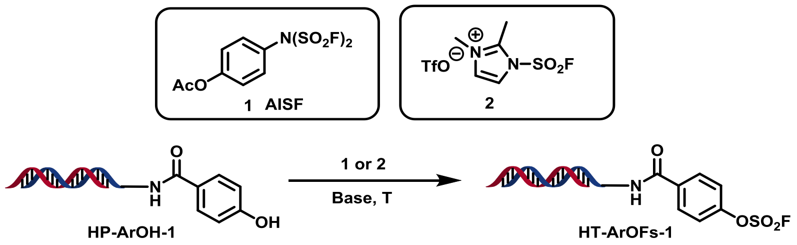

Entry	SO_2F	base	T(h)	Conversion (%)[b]
1	**1**	DBU	0.5	54
2	**1**	DBU	1	73
3	**1**	DBU	1.5	86
**4**	**1**	**DBU**	**2**	**96**
5	**1**	DBU	4	90
6	**2**	Et_3N	0.5	71
7	**2**	Et_3N	1	86
8	**2**	Et_3N	1.5	84
9	**2**	Et_3N	2	82
10	**2**	Et_3N	4	80

[a]Reaction conditions: 1 equiv of **HP-Ar-OH-1** (1 mM in borate buffer), 100 equiv of **1** (100 mM in THF) or **2** (100 mM in CH_3CN), 200 equiv of DBU (200 mM in THF) or Et_3N (200 mM in CH_3CN), borate buffer (pH 9.4); [b] Conversion of **HP-Ar-OH-1** determined by LC-MS.

Scheme S2. Preparation of DNA conjugated (hetero)aryl fluorosulfonates

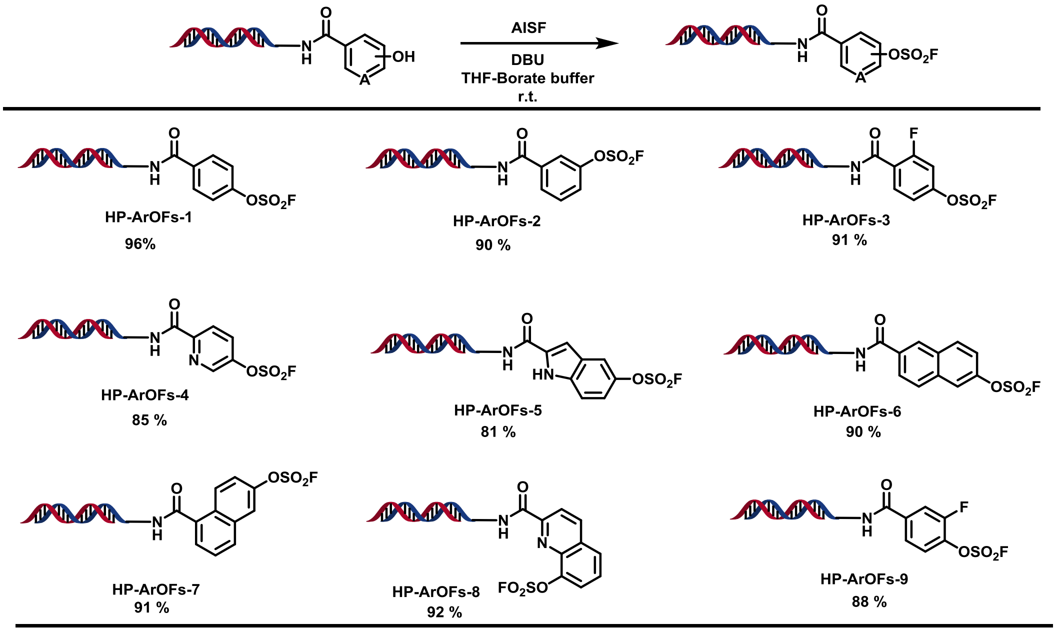

To the DNA conjugated phenols (300 μL, 1 mM), were added sequentially 300 μL AISF (100 mM), 300 μL borate buffer (250 mM, pH = 9.4), and 300 μL DBU (200

mM). The resultant mixture was vortexed and stood at 25 °C for 8 hours. 5.0M NaCl (120 μL) and cold neat ethanol (3.6 mL) were sequentially added, and the resultant mixture was incubated at −80 °C for at least 30 min. The mixture was centrifuged at 4 °C for 30 min at 12,000 rpm to remove the supernatant. The resulting pellet was re-dissolved in ddH_2O (300 μL), which was used in following reaction without further purification.

Table S2. Optimization of on-DNA Suzuki-Miyaura coupling reaction conditions[a]

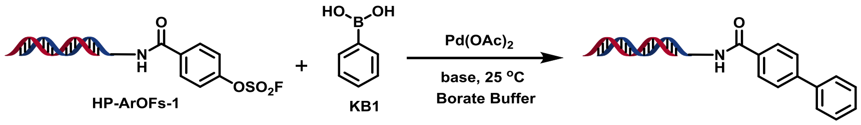

Entry	Base	Conversion (%)[b]
1	Et_3N	90
2	DIPEA	10
3	Na_2CO_3	0
4	K_2CO_3	0
5	NaOH	0

[a]Reaction conditions: 1 equiv of **HP-ArOFs-1** (1 mM in borate buffer), 400 equiv of **KB1** (400 mM in 1,4-dioxane), 20 equiv of $Pd(OAc)_2$ (20 mM in 1,4-dioxane), base (1000 mM in 1,4-dioxane), water; [b] Conversion of **HP-ArOFs-1** was determined by LC-MS.

General Procedures for on-DNA Suzuki-Miyaura Coupling of Representative Boronic Acids with HP-ArOFs-1

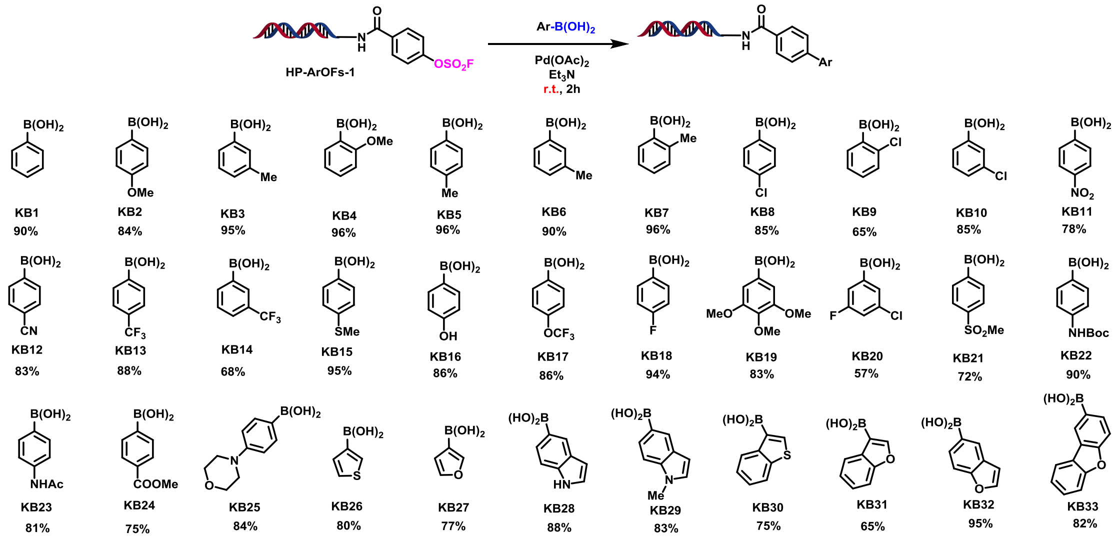

To **HP-ArOFs-1** (10 nmol, 10 μL, 1 mM in water), was added 400 equiv. of aromatic

boronic acids (10 μL, 400 mM in 1,4-dioxane) followed by 1000 equiv. of Et_3N (2 μL, 5 M in 1,4-dioxane), 20 equiv. of $Pd(OAc)_2$ (10 μL, 20 mM in 1,4-dioxane) and 20 μL H_2O. The mixture was vortexed and stood at 25 °C for 2 hours. After reaction, 30 equiv. of scavenger sodium diethyldithiocarbamic acid (compared with $Pd(OAc)_2$, 6 μL 1 M in ddH_2O) were added to the mixture, and the reaction mixture was heated at 60 °C for at least 30 minutes. The mixture was centrifuged at 4 °C for 30 min at 12,000 rpm, and the resultant supernatant was collected. Add 5 M NaCl solution (10% by volume) and cold ethanol (2.5 times by volume, ethanol stored at -20 °C) to the resultant supernatant. The mixture was vortexed and incubated at -80 °C for at least 30 minutes. The sample was centrifuged for 30 minutes at 4°C in a microcentrifuge at 12,000 rpm to remove the supernatant. The resulting pellet (precipitate) was re-dissolved in ddH_2O (300 μL) for LC-MS detection.

Scheme S3. Reactions of DNA conjugated (hetero) aryl fluorosulfonates with phenylboronic acid

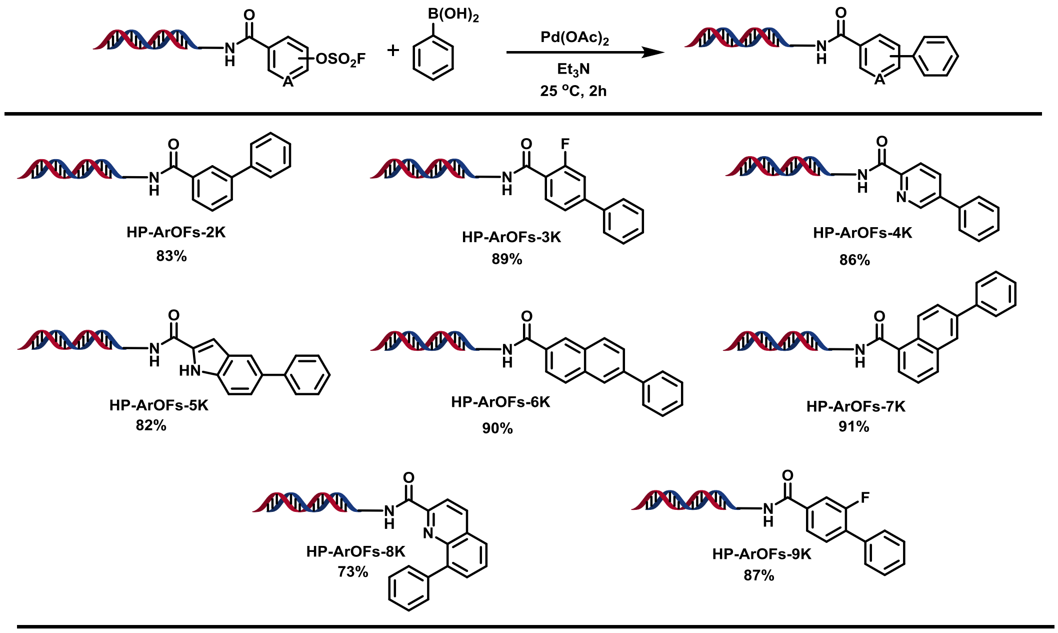

To the DNA conjugated (hetero) aryl fluorosulfates (10 nmol, 10 μL, 1 mM in ddH_2O), were added 400 equiv. of phenylboronic acid (10 μL, 400 mM in 1,4-dioxane) followed by 1000 equiv. of Et_3N (2 μL, 5 M in 1,4-dioxane), 20 equiv.

of $Pd(OAc)_2$ (10 μL, 20 mM in 1,4-dioxane), and 20 μL H_2O. The mixture was vortexed and stood at 25 °C for 2 hours. After reaction, 30 equiv. of scavenger sodium diethyldithiocarbamic acid (compared with $Pd(OAc)_2$, 6 μL 1000 mM in water) were added to the mixture, and the reaction mixture was heated at 60 °C for at least 30 minutes. The mixture was centrifuged at 4 °C for 30 minutes at 12,000 rpm, and the resultant supernatant was collected. Add 5 M NaCl solution (10% by volume) and cold ethanol (2.5 times by volume, ethanol stored at -20 °C) to the resultant supernatant. The mixture was vortexed and incubated at -80 °C for at least 30 minutes. The sample was centrifuged for 30 minutes at 4°C in a microcentrifuge at 12,000 rpm to remove the supernatant. The resulting pellet (precipitate) was re-dissolved in ddH_2O (300 μL) for LC-MS detection.

Table S3. Optimization of on-DNA Sonogoshira reaction conditions[a]

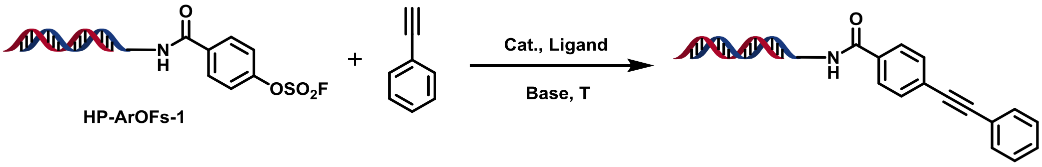

Entry	Cat.	Ligiand	Base	Conversion(%)[b]
**1**	$Pd(OAc)_2$	Xphos	Et_3N	0
**2**	$Pd(OAc)_2$	dppp	Et_3N	0
**3**	$Pd(OAc)_2$	Dppf	Et_3N	0
**4**	$Pd(OAc)_2$	P(t-Bu)3	Et_3N	0
**5**	$Pd(OAc)_2$	PPh3	Et_3N	0
**6**	$Pd(OAc)_2$	TPPS	Et_3N	0
**7**	$Pd(OAc)_2$	Xantphos	Et_3N	77
**8**	$Pd(PPh_3)_4$	Xantphos	Et_3N	68
**9**	$Pd_2(dba)_3$	Xantphos	Et_3N	48
**10**	$Pd(PPh_3)_2Cl_2$	Xantphos	Et_3N	46
**11**	$[PdCl(C_3H_5)]_2$	Xantphos	Et_3N	72
**12**	$Pd(OAc)_2$	Xantphos	DBU	0
**13**	$Pd(OAc)_2$	Xantphos	DIPEA	56
**14**	$Pd(OAc)_2$	Xantphos	DABCO	72
**15**	$Pd(OAc)_2$	Xantphos	NaHCO3	67
**16**	$Pd(OAc)_2$	Xantphos	Na2CO3	43
**17**	$Pd(OAc)_2$	Xantphos	K2CO3	32
**18**	$Pd(OAc)_2$	Xantphos	Cs2CO3	64
**19**	$Pd(OAc)_2$	Xantphos	\	62
**20**[c]	$Pd(OAc)_2$	Xantphos	Et_3N	0
**21**[d]	$Pd(OAc)_2$	Xantphos	Et_3N	41
**22**[e]	$Pd(OAc)_2$	Xantphos	Et_3N	69
**23**[f]	**$Pd(OAc)_2$**	**Xantphos**	**Et_3N**	**85**

[a]Reaction conditions: 1 equiv of **HP-ArOFs-1** (1 mM in ddH_2O), 200 equiv of 1-ethynyl-4-methoxybenzene (200 mM in DMA), 10 equiv of Cat. (10 mM in DMA), 20 equiv of ligand (20 mM in DMA), 1000 equiv of base (5 M in DMA), 60 ºC, 2h; [b]Conversion

of **HP-ArOFs-1** was determined by LC-MS; [c]25 ºC was used; [d]40 ºC was used; [e]80 ºC was used; [f]20 equiv of $Pd(OAc)_2$ (20 mM in DMA), 40 equiv of Xantphos (40 mM in DMA), 1000 equiv of Et_3N (5 M in DMA).

General Procedures for on-DNA Sonogashira Coupling of Representative Alkynes and HP-ArOFs-1

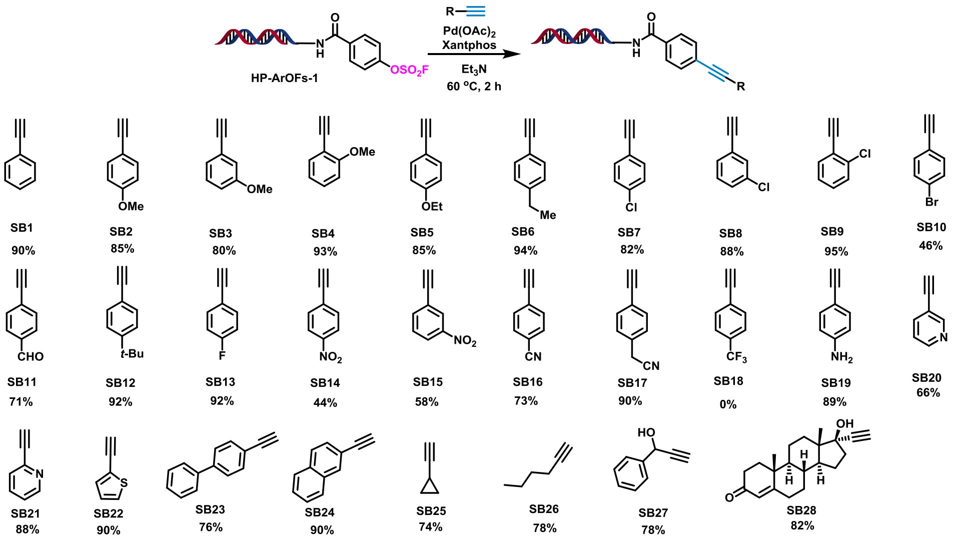

$Pd(OAc)_2$ (10 μL, 20 mM in DMA), Xantphos (10 μL, 40 mM in DMA) and alkynes (10 μL, 200 mM in DMA) were mixed to generate 30 μL 0.67 mM Pd / Xantphos / alkynes complex. To **HP-ArOFs-1** (10 nmol, 10 μL, 1 mM in ddH_2O), were added 1000 equiv. of Et_3N (2 μL, 5 M in DMA) and the above 30 μL 0.67 mM Pd / Xantphos / alkynes complex. The resulting mixture was vortexed and stood at 60 °C for 2 hours. After incubation, 30 equiv. of scavenger sodium diethyldithiocarbamic acid (compared with $Pd(OAc)_2$, 6 μL 1 M in ddH_2O) were added to the mixture, and heated at 60 °C for at least 30 minutes. The reaction mixture was next centrifuged at 4 °C for 30 min at 12,000 rpm, and the resultant supernatant was collected. To the supernatant, 5 M NaCl solution (10% by volume) and cold ethanol (2.5 times by volume, ethanol stored at -20°C) were added, vortexed, and incubated at -80°C for at least 30 minutes. The sample was centrifuged for 30 minutes at 4°C in a microcentrifuge at 12,000 rpm to remove the supernatant. The resulting pellet (precipitate) was re-dissolved in ddH2O (300 μL) for LC-MS detection.

Scheme S4. Reactions of DNA conjugated (hetero) aryl fluorosulfonates with 1-ethynyl-benzene

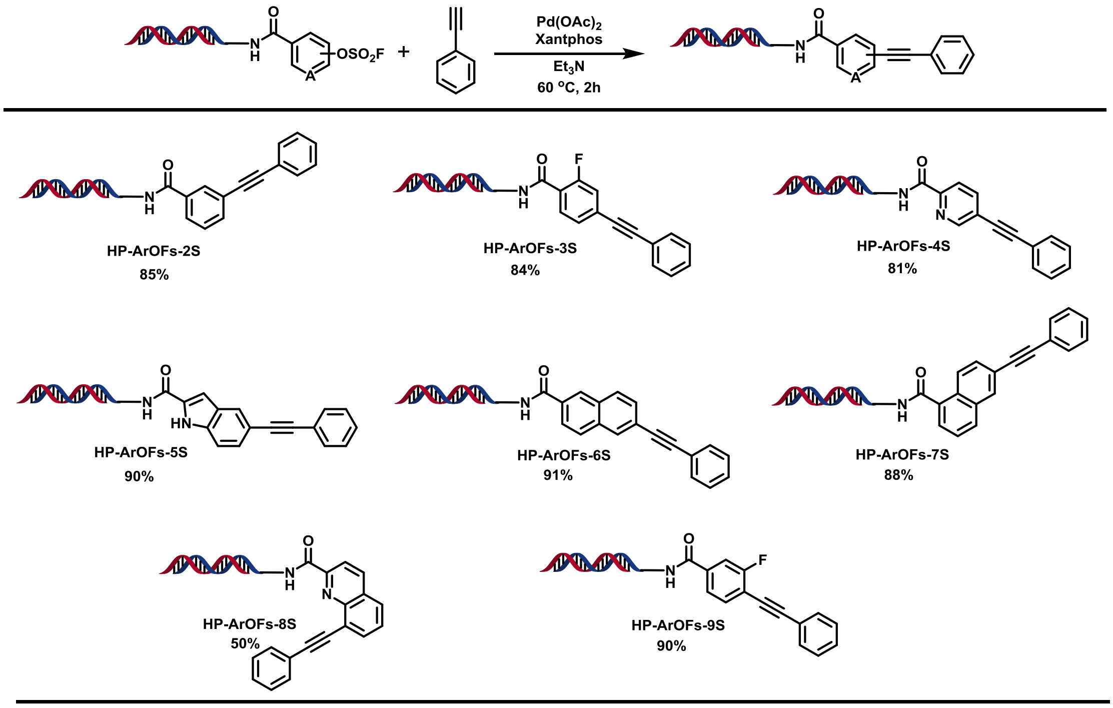

$Pd(OAc)_2$ (10 μL, 20 mM in DMA), Xantphos (10 μL, 40 mM in DMA) and Phenylacetylene (10 μL, 200 mM in DMA) were mixed to generate 30 μL 0.67 mM Pd / Xantphos / Phenylacetylene complex. To the DNA conjugated (hetero) aryl fluorosulfates (10 nmol, 10 μL, 1 mM in ddH_2O), was added 1000 equiv. of Et_3N (2 μL, 5 M in DMA) and the above 30μL 0.67 mM Pd / Xantphos / alkynes complex. The mixture was vortexed and stood at 60 °C for 2 hours. After incubation, 30 equiv. of scavenger sodium diethyldithiocarbamic acid (compared with $Pd(OAc)_2$, 6 μL 100 mM in ddH_2O) were added to the mixture, and heated at 60 °C for at least 30 minutes. The reaction mixture was centrifuged at 4 °C for 30 min at 12,000 rpm, and the resultant supernatant was collected. 5 M NaCl solution (10% by volume) and cold ethanol (2.5 times by volume, ethanol stored at -20°C) were added to the resultant supernatant. The resulting mixture was vortexed and incubated at -80°C for at least 30 minutes. The sample was centrifuged for 30 minutes at 4°C in a microcentrifuge at 12,000 rpm to remove the supernatant. The resulting pellet (precipitate) was re-dissolved in ddH_2O (300 μL) for LC-MS detection.

Table S4. Optimization of on-DNA Buchwald reaction conditions[a]

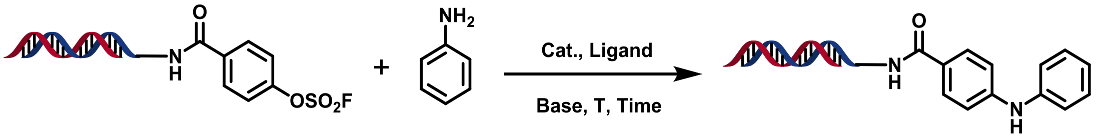

entry	Cat	ligiand	Base	Conversion (%) [b]
**1**	$Pd(OAc)_2$	Xphos	Et_3N	0
**2**	$Pd(OAc)_2$	dppp	Et_3N	0
**3**	$Pd(OAc)_2$	Dppf	Et_3N	0
**4**	$Pd(OAc)_2$	P(t-Bu)3	Et_3N	0
**5**	$Pd(OAc)_2$	PPh3	Et_3N	0
**6**	$Pd(OAc)_2$	TPPS	Et_3N	0
**7**	$Pd(OAc)_2$	Xantphos	Et_3N	0
**8**	$Pd(PPh_3)_4$	BINAP	Et_3N	0
**9**	Brettphos	\	Et_3N	89
**10**	t-BuBrettphos	\	Et_3N	91
**11**[c]	t-BuBrettphos	\	Et_3N	92
**12**[d]	**t-BuBrettphos**	****	**Et_3N**	**93**
**13**[e]	t-BuBrettphos	\	Et_3N	87
**14**[f]	t-BuBrettphos	\	Et_3N	83

[a] Reaction conditions: 1 equiv of **HP-ArOFs-1** (1 mM in water), 200 equiv of aniline (200 mM in DMA), 20 equiv of Cat. (10 mM in DMA), 40 equiv of ligand (20 mM in DMA), 1000 equiv of base (5000 mM in DMA), 60 °C, 2 hours; [b] Conversion of **HP-ArOFs-1** was determined by LC-MS; [c] 15 equiv of t-Brettphos Pd G3; [d] 10 equiv of t-Brettphos Pd G3; [e] 5 equiv of t-Brettphos Pd G3; [f] 3 equiv of t-Brettphos Pd G3.

General Procedures for on-DNA Buchwald Amination of Representative Amines with HP-ArOFs-1

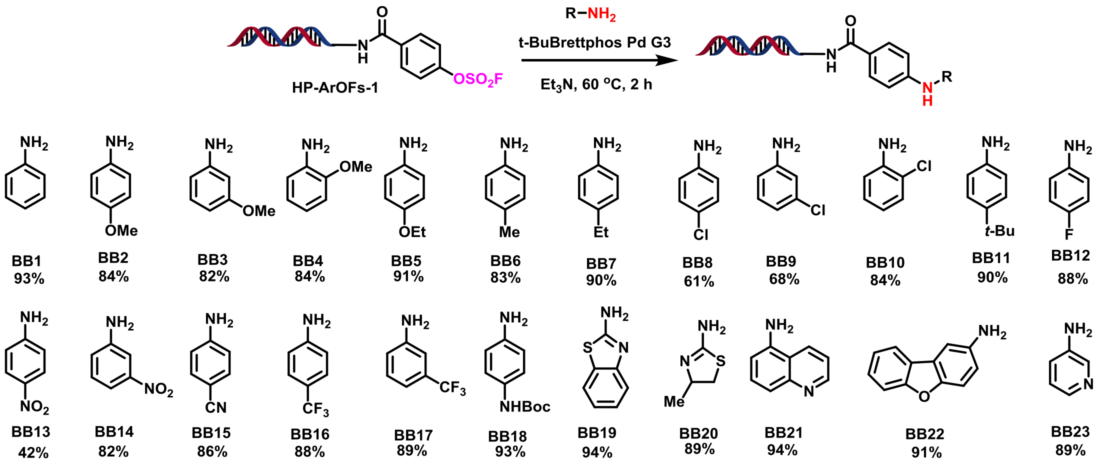

To **HP-ArOFs-1** (10 nmol, 10 μL, 1 mM in ddH$_2$O), was added 200 equiv. of aromatic amines (10 μL, 200 mM in DMA), followed by 1000 equiv. of Et$_3$N (2 μL, 5 M in DMA) and 10 equiv. of t-BuBrettphos Pd G3 (10 μL, 10 mM in DMA). The mixture was vortexed and stood at 60 °C for 2 hours. After the reaction, 30 equiv. of scavenger sodium diethyldithiocarbamic acid (compared with Pd(OAc)$_2$, 6 μL 1 M in ddH$_2$O) were added to the mixture, and the resulting mixture was heated at 60 °C for at least 30 minutes followed by centrifugation at 4 °C for 30 min at 12,000 rpm. The resultant supernatant was collected, mixed with 5 M NaCl solution (10% by volume) and cold ethanol (2.5 times by volume, ethanol stored at -20 °C), vortexed, and incubated at -80°C for at least 30 minutes. The sample was centrifuged for 30 minutes at 4°C in a micro centrifuge at 12,000 rpm to remove the supernatant. The resulting pellet (precipitate) was re-dissolved in ddH$_2$O (300 μL) for LC-MS detection.

Scheme S5. Reactions of DNA conjugated (hetero) aryl fluorosulfonates with anilines

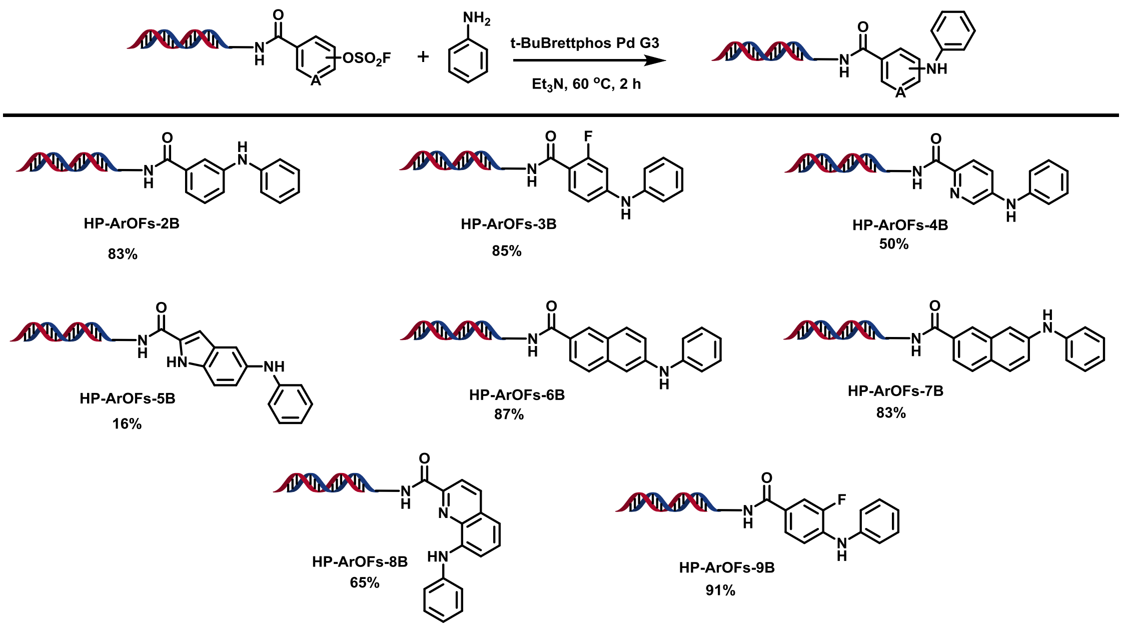

To DNA conjugated (hetero) aryl fluorosulfates (10 nmol, 10 μL, 1 mM in water), were added 200 equiv. of aniline (10 μL, 200 mM in DMA), followed by 1000 equiv. of Et_3N (2 μL, 5 M in DMA) and 10 equiv. of *t*-BuBrettphos Pd G3 (10 μL, 10 mM in DMA). The mixture was vortexed and stood at 60 °C for 2 hours. After the reaction, 30 equiv. of scavenger sodium diethyldithiocarbamic acid (compared with *t*-BuBrettphos Pd G3, 6 μL 1 M in ddH_2O) were added to the mixture, and the reaction mixture was heated at 60 °C for at least 30 minutes. The resulting mixture was centrifuged at 4 °C for 30 min at 12,000 rpm, and the resultant supernatant was collected. 5 M NaCl solution (10% by volume) and cold ethanol (2.5 times by volume, ethanol stored at -20°C) were added to the resultant supernatant, and the mixture was vortexed and incubated at -80°C for at least 30 minutes. The sample was centrifuged for 30 minutes at 4°C in a microcentrifuge at 12,000 rpm to remove the supernatant. The resulting pellet (precipitate) was re-dissolved in ddH_2O (300 μL) for LC-MS detection.

MS Spectra of DNA conjugated phenols

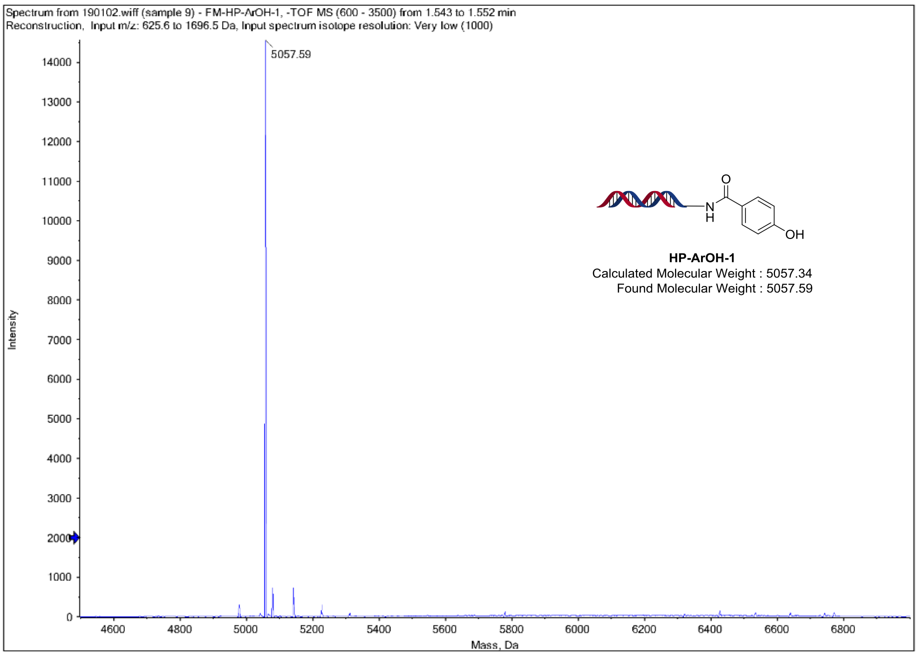

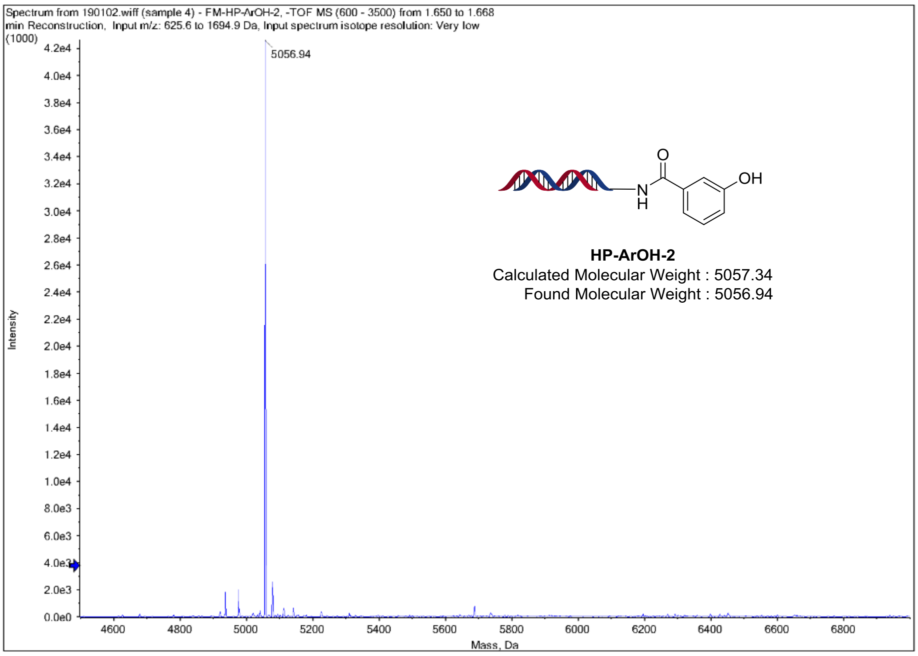

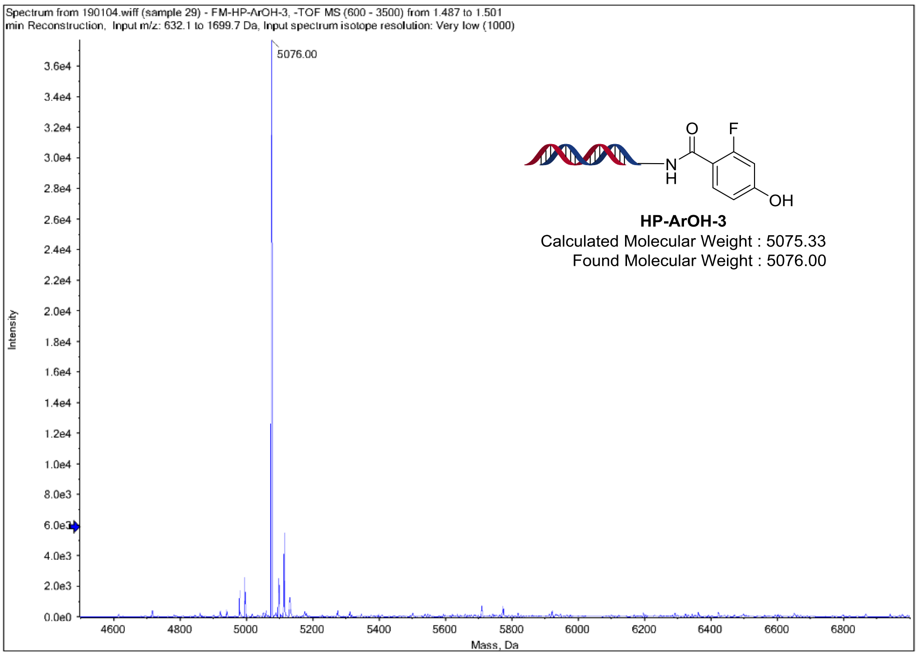

Spectrum from 190104.wiff (sample 29) - FM-HP-ArOH-3, -TOF MS (600 - 3500) from 1.487 to 1.501 min Reconstruction, Input m/z: 632.1 to 1699.7 Da, Input spectrum isotope resolution: Very low (1000)
5076.00
Intensity
Mass, Da
HP-ArOH-3
Calculated Molecular Weight : 5075.33
Found Molecular Weight : 5076.00

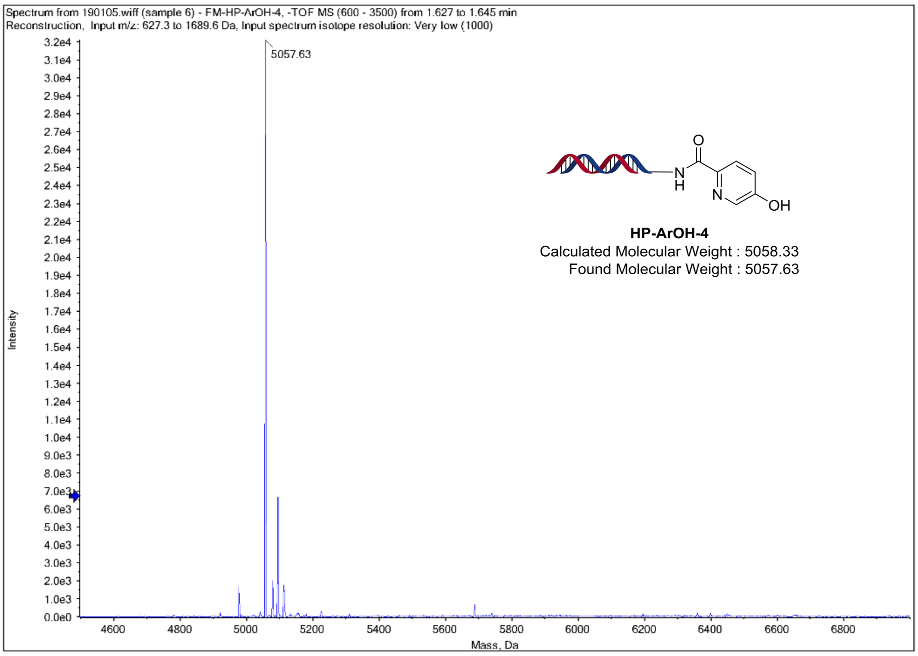

Spectrum from 190105.wiff (sample 6) - FM-HP-ArOH-4, -TOF MS (600 - 3500) from 1.627 to 1.645 min Reconstruction, Input m/z: 627.3 to 1689.6 Da, Input spectrum isotope resolution: Very low (1000)
5057.63
Intensity
Mass, Da
HP-ArOH-4
Calculated Molecular Weight : 5058.33
Found Molecular Weight : 5057.63

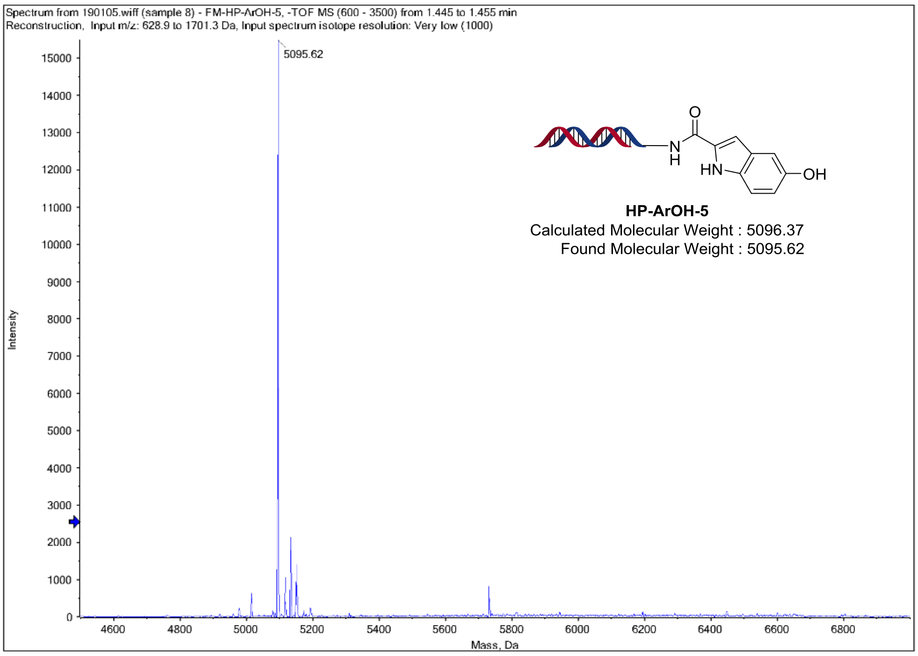
Spectrum from 190105.wiff (sample 8) - FM-HP-ArOH-5, -TOF MS (600 - 3500) from 1.445 to 1.455 min
Reconstruction, Input m/z: 628.9 to 1701.3 Da, Input spectrum isotope resolution: Very low (1000)
5095.62
Intensity
Mass, Da
HP-ArOH-5
Calculated Molecular Weight : 5096.37
Found Molecular Weight : 5095.62

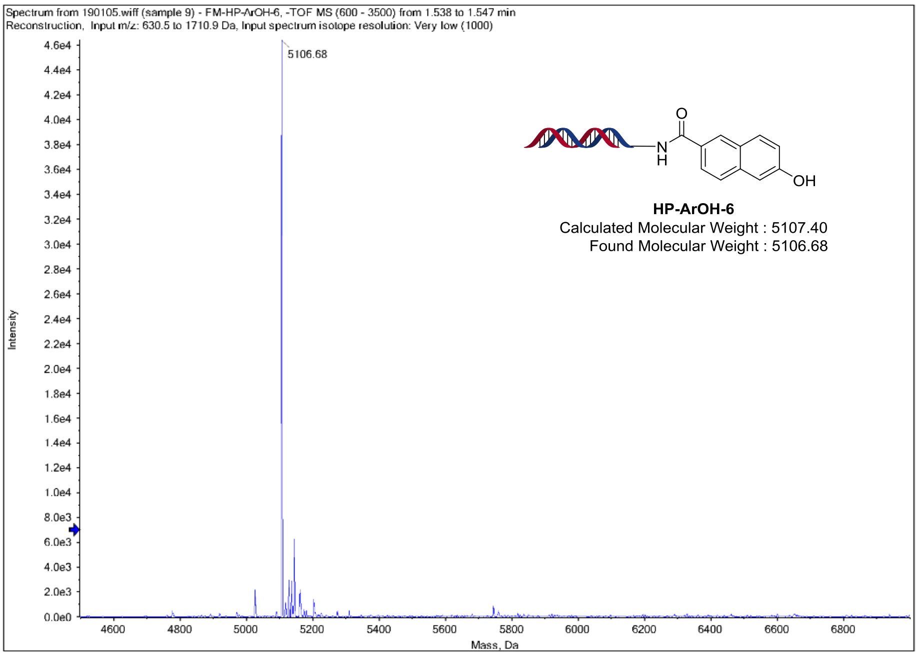
Spectrum from 190105.wiff (sample 9) - FM-HP-ArOH-6, -TOF MS (600 - 3500) from 1.538 to 1.547 min
Reconstruction, Input m/z: 630.5 to 1710.9 Da, Input spectrum isotope resolution: Very low (1000)
5106.68
Intensity
Mass, Da
HP-ArOH-6
Calculated Molecular Weight : 5107.40
Found Molecular Weight : 5106.68

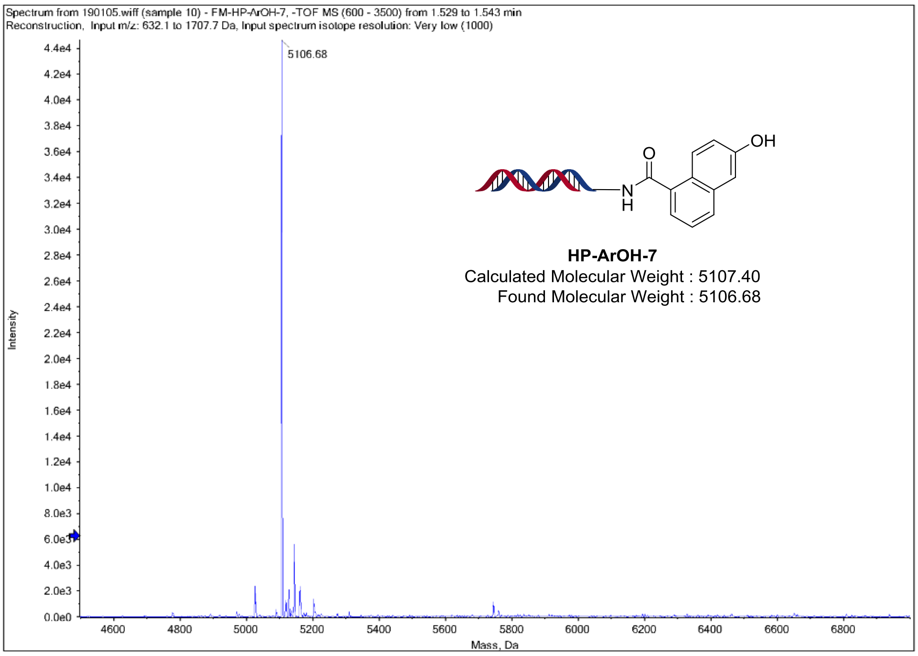
Spectrum from 190105.wiff (sample 10) - FM-HP-ArOH-7, -TOF MS (600 - 3500) from 1.529 to 1.543 min
Reconstruction, Input m/z: 632.1 to 1707.7 Da, Input spectrum isotope resolution: Very low (1000)
5106.68
Intensity
Mass, Da
OH
O
N
H
HP-ArOH-7
Calculated Molecular Weight : 5107.40
Found Molecular Weight : 5106.68

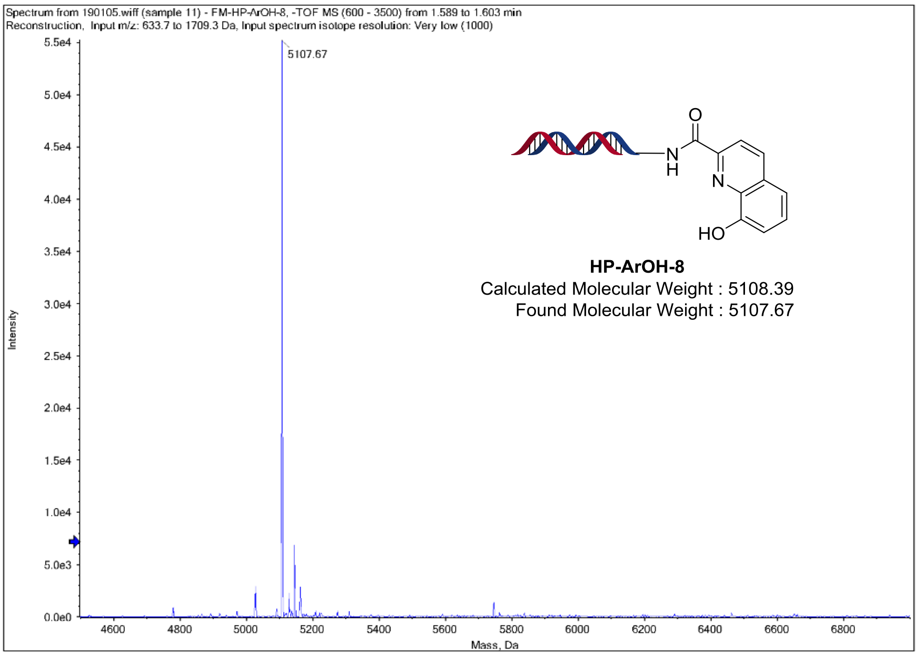
Spectrum from 190105.wiff (sample 11) - FM-HP-ArOH-8, -TOF MS (600 - 3500) from 1.589 to 1.603 min
Reconstruction, Input m/z: 633.7 to 1709.3 Da, Input spectrum isotope resolution: Very low (1000)
5107.67
Intensity
Mass, Da
O
N
H
N
HO
HP-ArOH-8
Calculated Molecular Weight : 5108.39
Found Molecular Weight : 5107.67

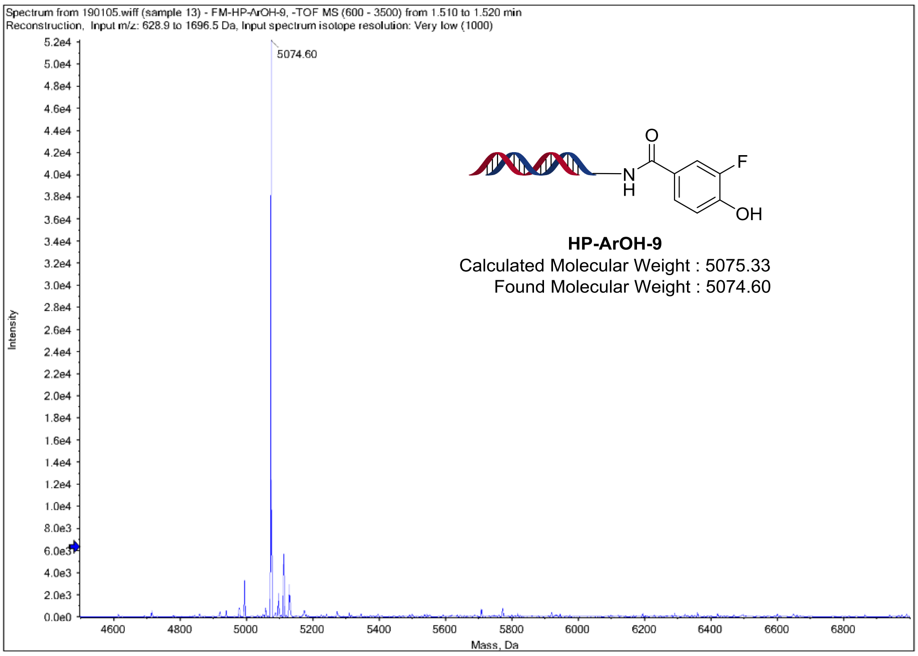
Spectrum from 190105.wiff (sample 13) - FM-HP-ArOH-9, -TOF MS (600 - 3500) from 1.510 to 1.520 min
Reconstruction, Input m/z: 628.9 to 1696.5 Da, Input spectrum isotope resolution: Very low (1000)
5074.60
Intensity
Mass, Da
HP-ArOH-9
Calculated Molecular Weight : 5075.33
Found Molecular Weight : 5074.60

MS Spectra of DNA conjugated (hetero)aryl fluorosulfonates.

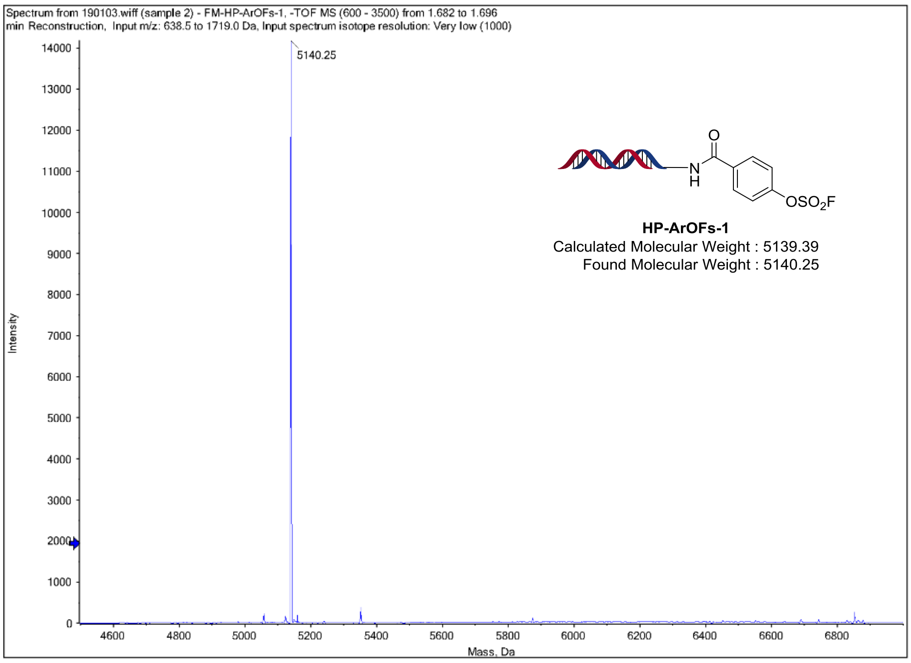

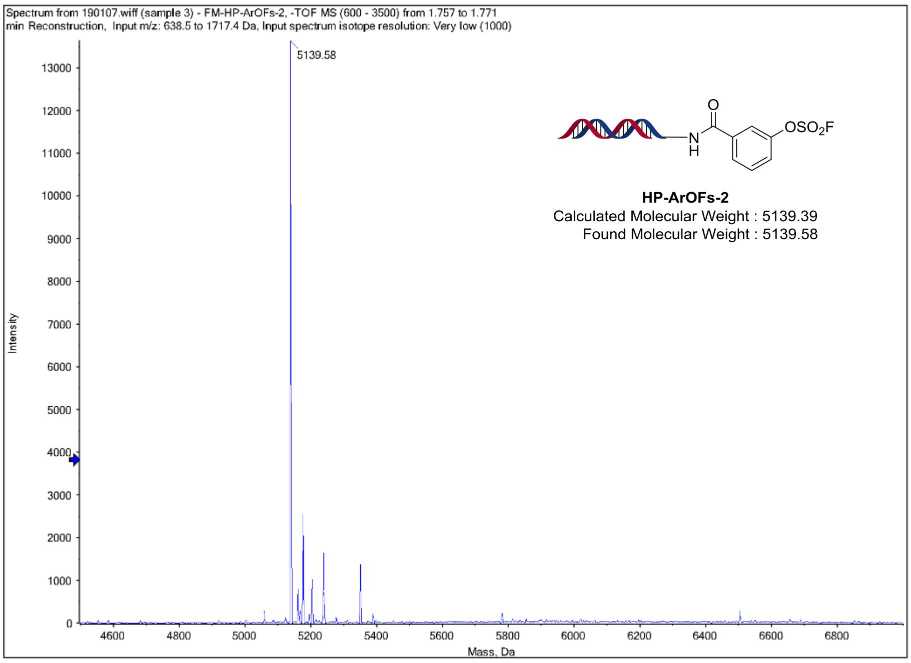

Spectrum from 190107.wiff (sample 3) - FM-HP-ArOFs-2, -TOF MS (600 - 3500) from 1.757 to 1.771 min Reconstruction, Input m/z: 638.5 to 1717.4 Da, Input spectrum isotope resolution: Very low (1000)
5139.58
Intensity
Mass, Da
HP-ArOFs-2
Calculated Molecular Weight : 5139.39
Found Molecular Weight : 5139.58

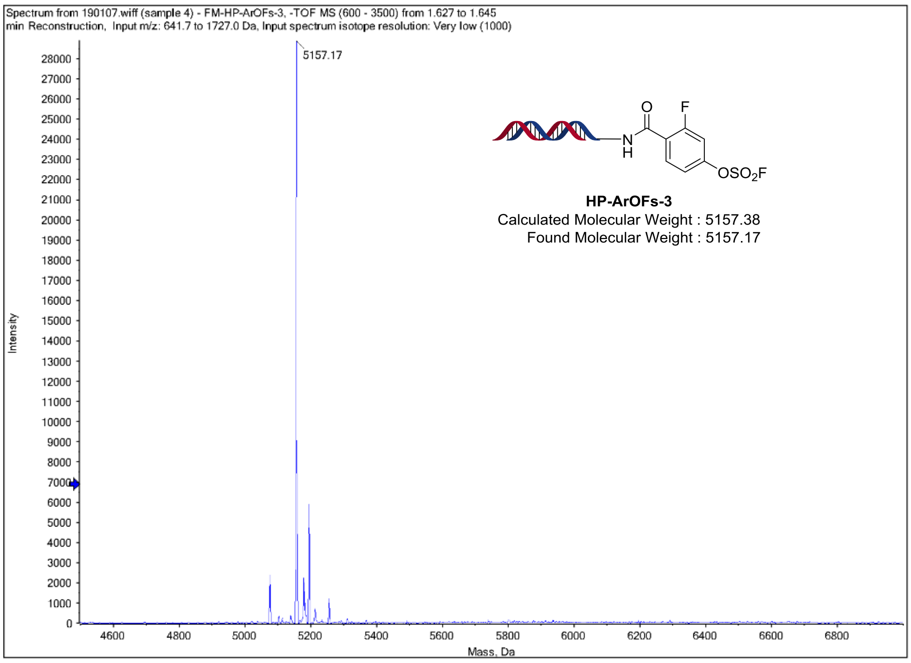

Spectrum from 190107.wiff (sample 4) - FM-HP-ArOFs-3, -TOF MS (600 - 3500) from 1.627 to 1.645 min Reconstruction, Input m/z: 641.7 to 1727.0 Da, Input spectrum isotope resolution: Very low (1000)
5157.17
Intensity
Mass, Da
HP-ArOFs-3
Calculated Molecular Weight : 5157.38
Found Molecular Weight : 5157.17

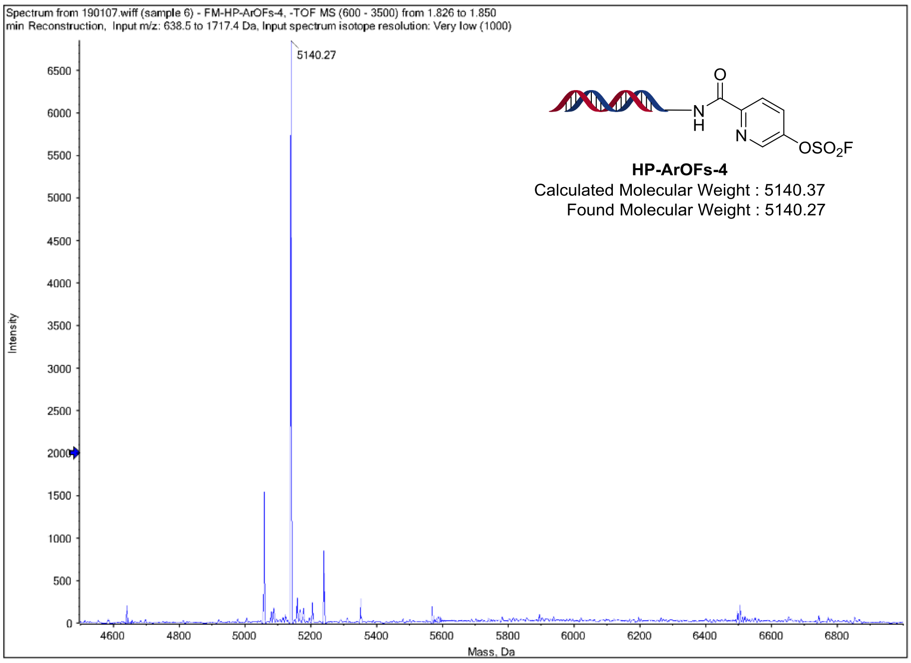

Spectrum from 190107.wiff (sample 6) - FM-HP-ArOFs-4, -TOF MS (600 - 3500) from 1.826 to 1.850 min Reconstruction, Input m/z: 638.5 to 1717.4 Da, Input spectrum isotope resolution: Very low (1000)
5140.27
HP-ArOFs-4
Calculated Molecular Weight : 5140.37
Found Molecular Weight : 5140.27
Intensity
Mass, Da

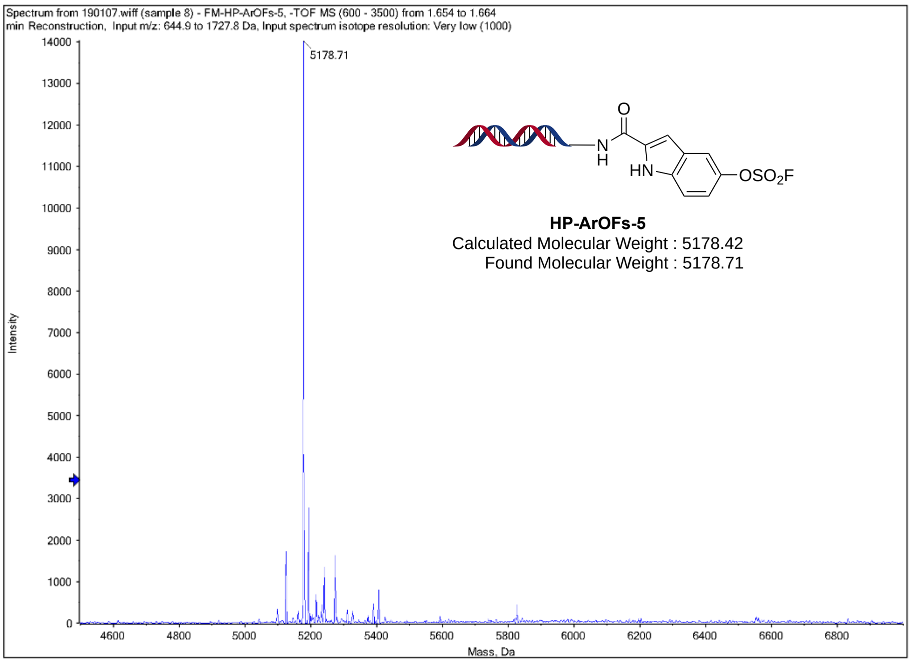

Spectrum from 190107.wiff (sample 8) - FM-HP-ArOFs-5, -TOF MS (600 - 3500) from 1.654 to 1.664 min Reconstruction, Input m/z: 644.9 to 1727.8 Da, Input spectrum isotope resolution: Very low (1000)
5178.71
HP-ArOFs-5
Calculated Molecular Weight : 5178.42
Found Molecular Weight : 5178.71
Intensity
Mass, Da

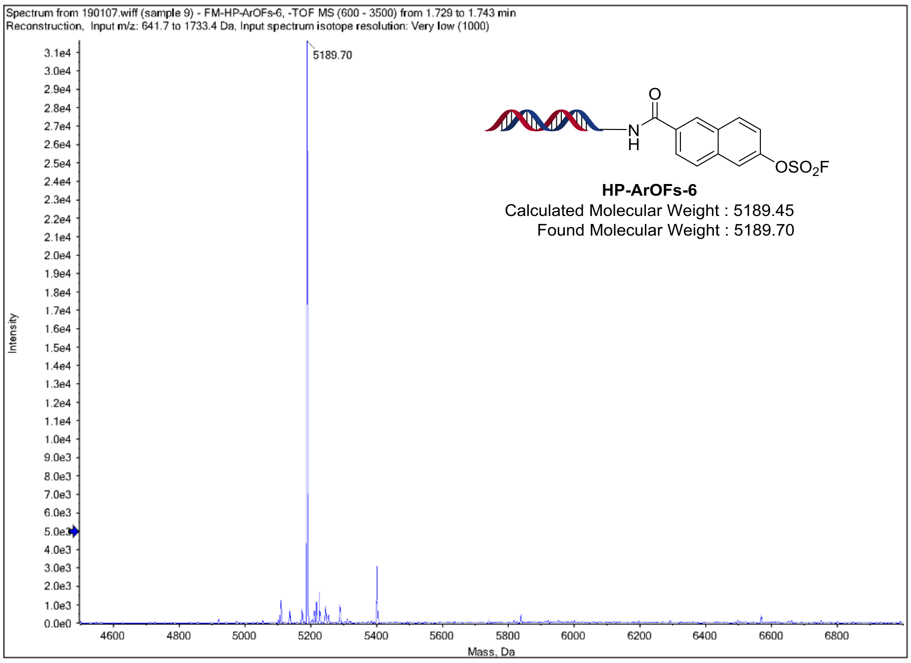

Spectrum from 190107.wiff (sample 9) - FM-HP-ArOFs-6, -TOF MS (600 - 3500) from 1.729 to 1.743 min
Reconstruction, Input m/z: 641.7 to 1733.4 Da, Input spectrum isotope resolution: Very low (1000)
5189.70
O
N
H
OSO2F
HP-ArOFs-6
Calculated Molecular Weight : 5189.45
Found Molecular Weight : 5189.70
Intensity
Mass, Da

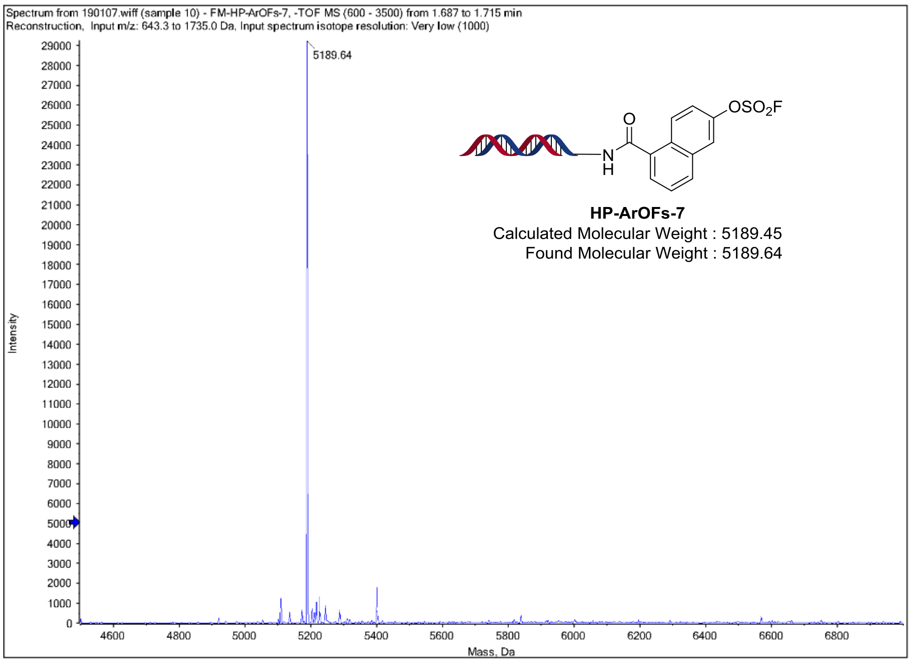

Spectrum from 190107.wiff (sample 10) - FM-HP-ArOFs-7, -TOF MS (600 - 3500) from 1.687 to 1.715 min
Reconstruction, Input m/z: 643.3 to 1735.0 Da, Input spectrum isotope resolution: Very low (1000)
5189.64
OSO2F
O
N
H
HP-ArOFs-7
Calculated Molecular Weight : 5189.45
Found Molecular Weight : 5189.64
Intensity
Mass, Da

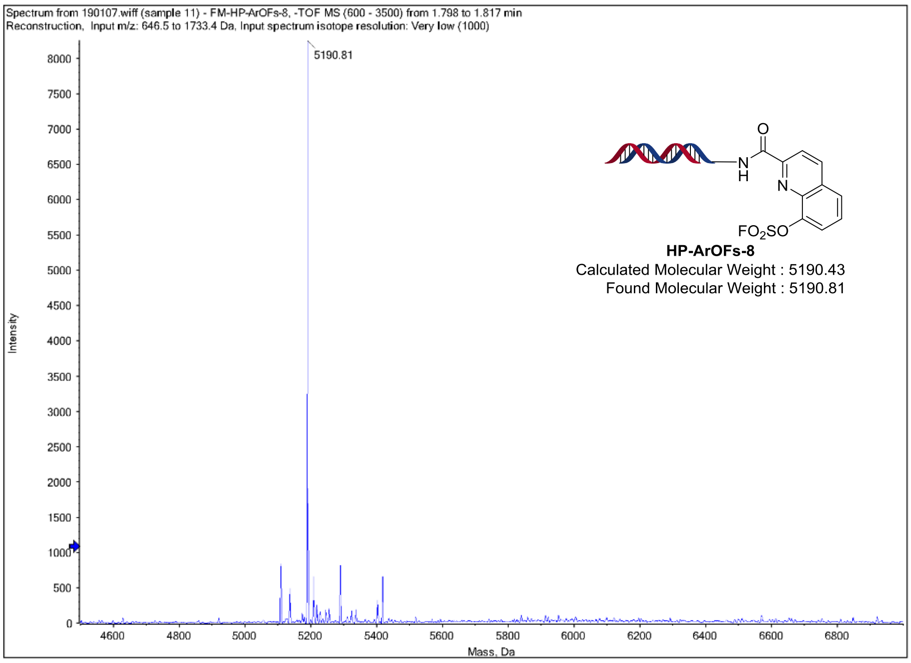
Spectrum from 190107.wiff (sample 11) - FM-HP-ArOFs-8, -TOF MS (600 - 3500) from 1.798 to 1.817 min
Reconstruction, Input m/z: 646.5 to 1733.4 Da, Input spectrum isotope resolution: Very low (1000)
5190.81
HP-ArOFs-8
Calculated Molecular Weight : 5190.43
Found Molecular Weight : 5190.81
Intensity
Mass, Da

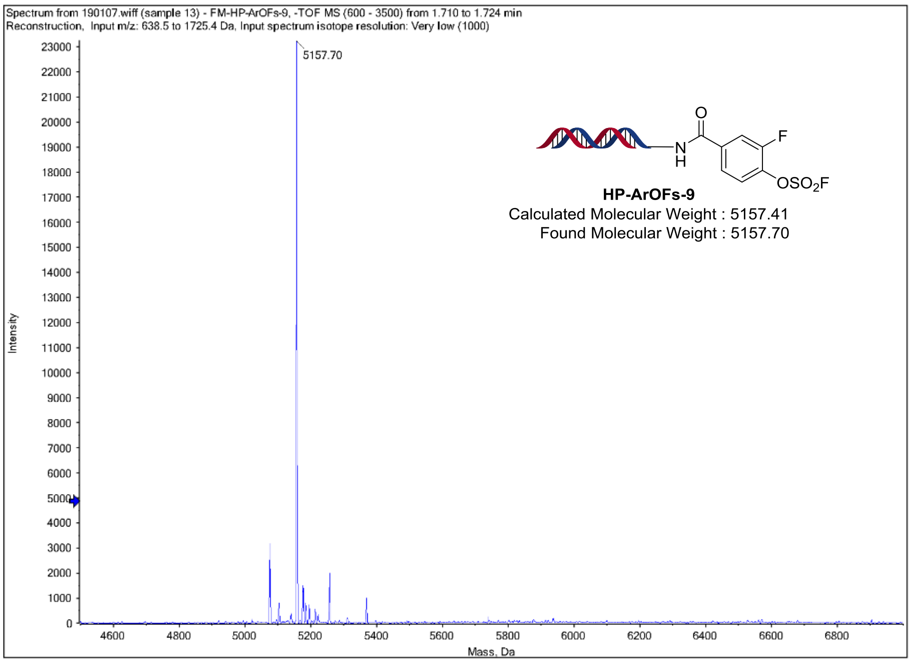
Spectrum from 190107.wiff (sample 13) - FM-HP-ArOFs-9, -TOF MS (600 - 3500) from 1.710 to 1.724 min
Reconstruction, Input m/z: 638.5 to 1725.4 Da, Input spectrum isotope resolution: Very low (1000)
5157.70
HP-ArOFs-9
Calculated Molecular Weight : 5157.41
Found Molecular Weight : 5157.70
Intensity
Mass, Da

MS Spectra of On-DNA Suzuki-Miyaura coupling products

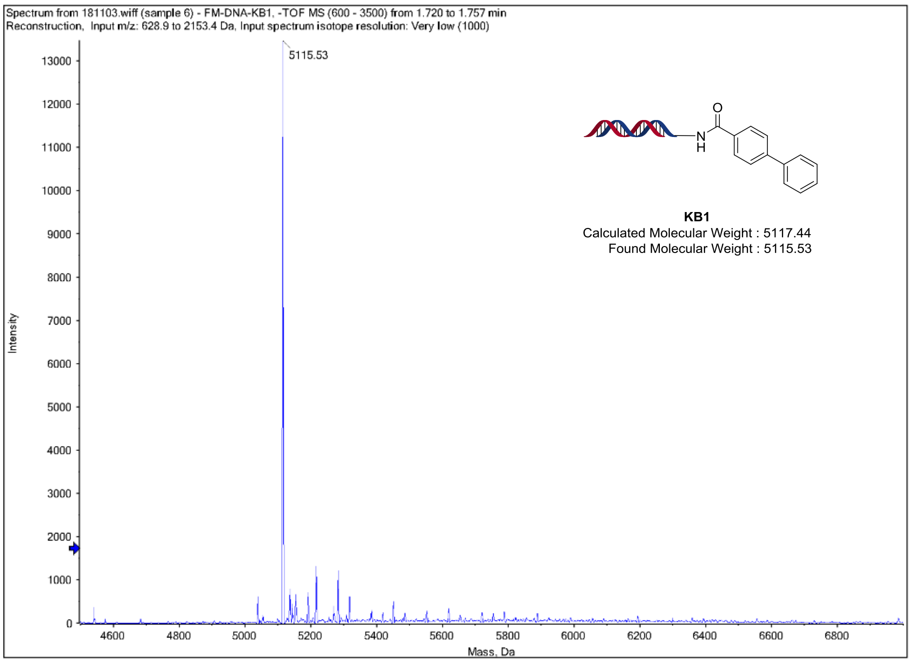

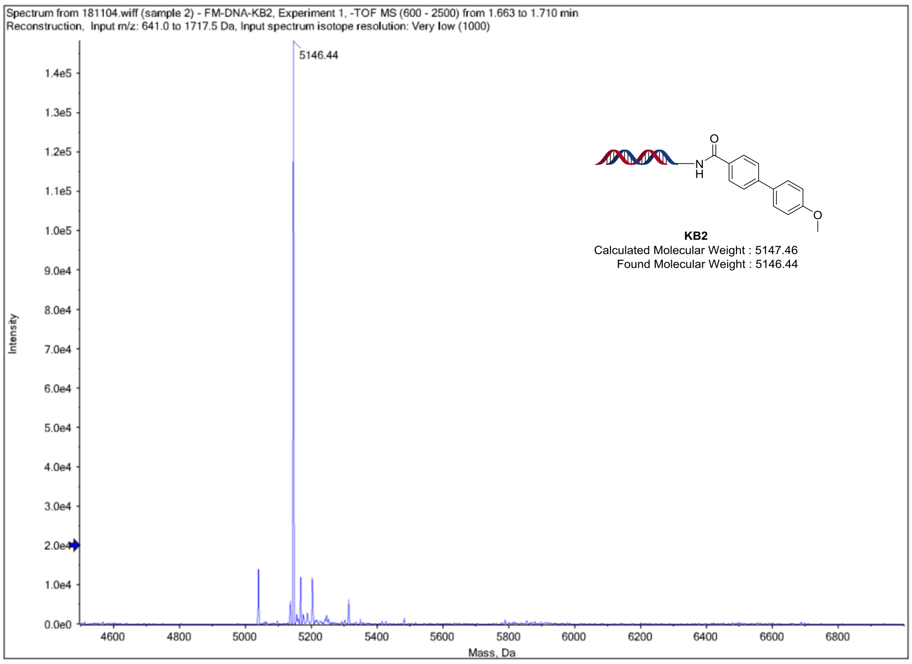
Spectrum from 181104.wiff (sample 2) - FM-DNA-KB2, Experiment 1, -TOF MS (600 - 2500) from 1.663 to 1.710 min
Reconstruction, Input m/z: 641.0 to 1717.5 Da, Input spectrum isotope resolution: Very low (1000)
5146.44
Intensity
Mass, Da
KB2
Calculated Molecular Weight : 5147.46
Found Molecular Weight : 5146.44

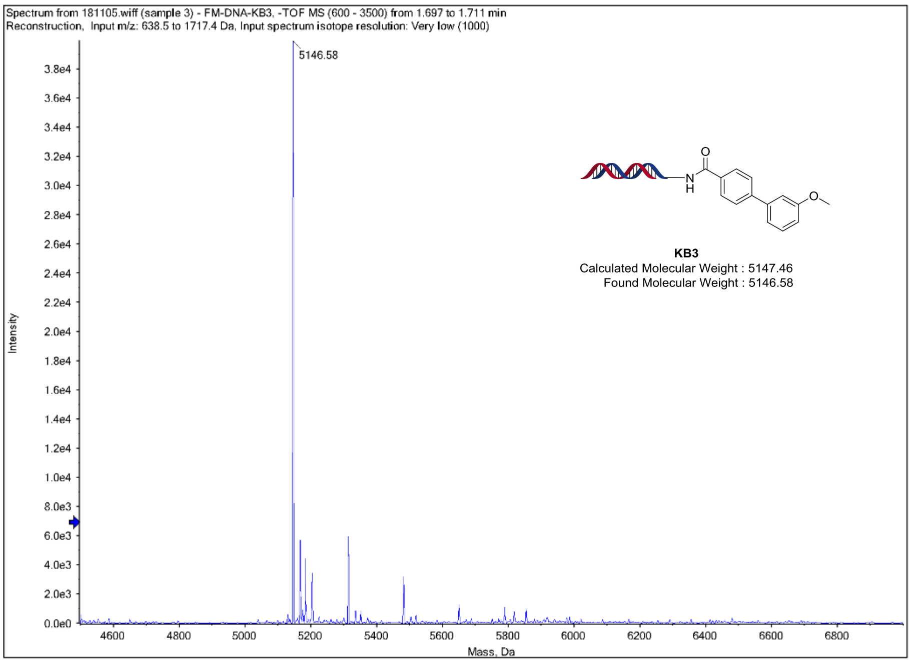
Spectrum from 181105.wiff (sample 3) - FM-DNA-KB3, -TOF MS (600 - 3500) from 1.697 to 1.711 min
Reconstruction, Input m/z: 638.5 to 1717.4 Da, Input spectrum isotope resolution: Very low (1000)
5146.58
Intensity
Mass, Da
KB3
Calculated Molecular Weight : 5147.46
Found Molecular Weight : 5146.58

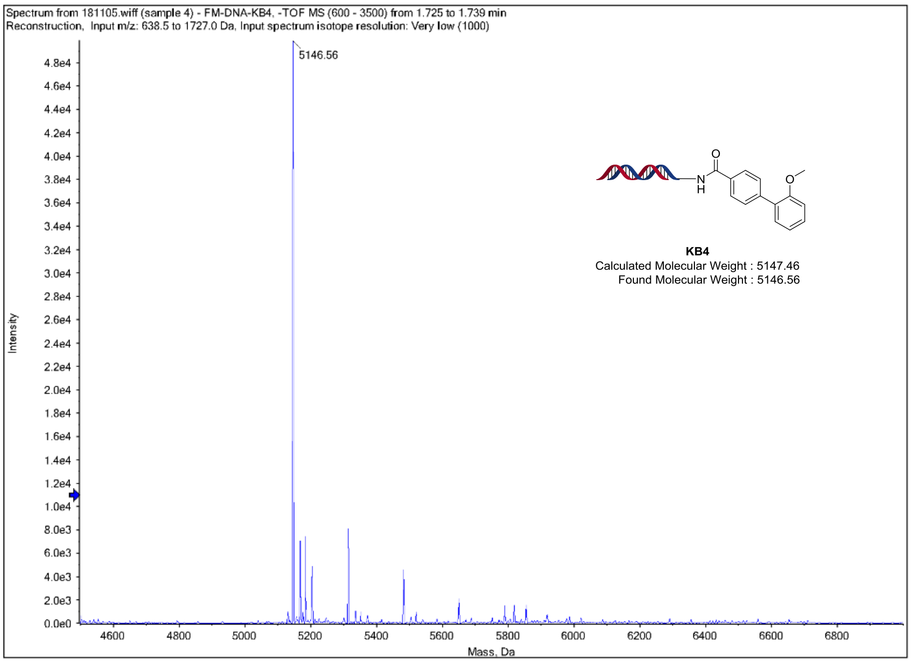
Spectrum from 181105.wiff (sample 4) - FM-DNA-KB4, -TOF MS (600 - 3500) from 1.725 to 1.739 min
Reconstruction, Input m/z: 638.5 to 1727.0 Da, Input spectrum isotope resolution: Very low (1000)
5146.56
KB4
Calculated Molecular Weight : 5147.46
Found Molecular Weight : 5146.56
Intensity
Mass, Da

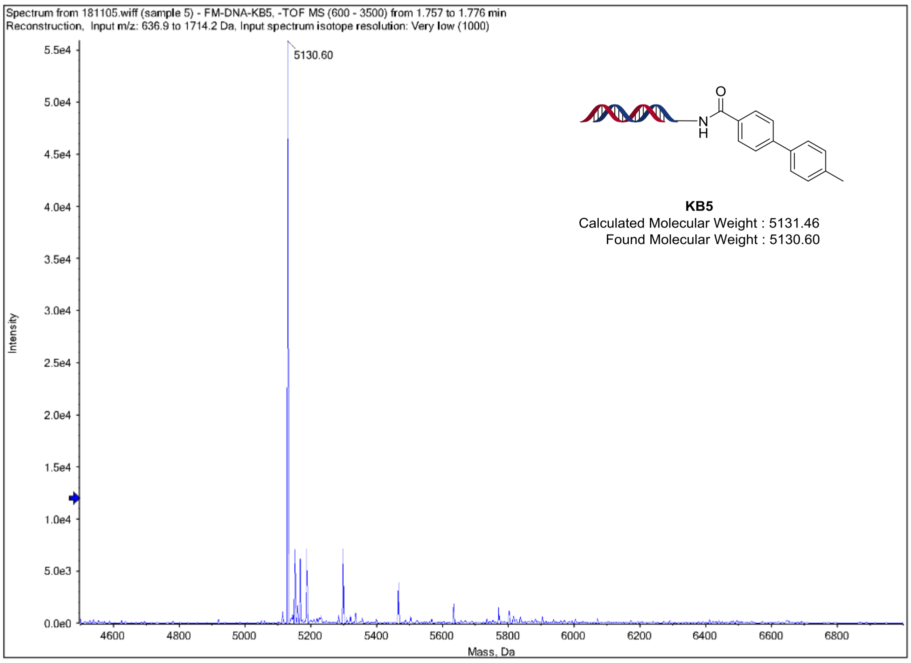
Spectrum from 181105.wiff (sample 5) - FM-DNA-KB5, -TOF MS (600 - 3500) from 1.757 to 1.776 min
Reconstruction, Input m/z: 636.9 to 1714.2 Da, Input spectrum isotope resolution: Very low (1000)
5130.60
KB5
Calculated Molecular Weight : 5131.46
Found Molecular Weight : 5130.60
Intensity
Mass, Da

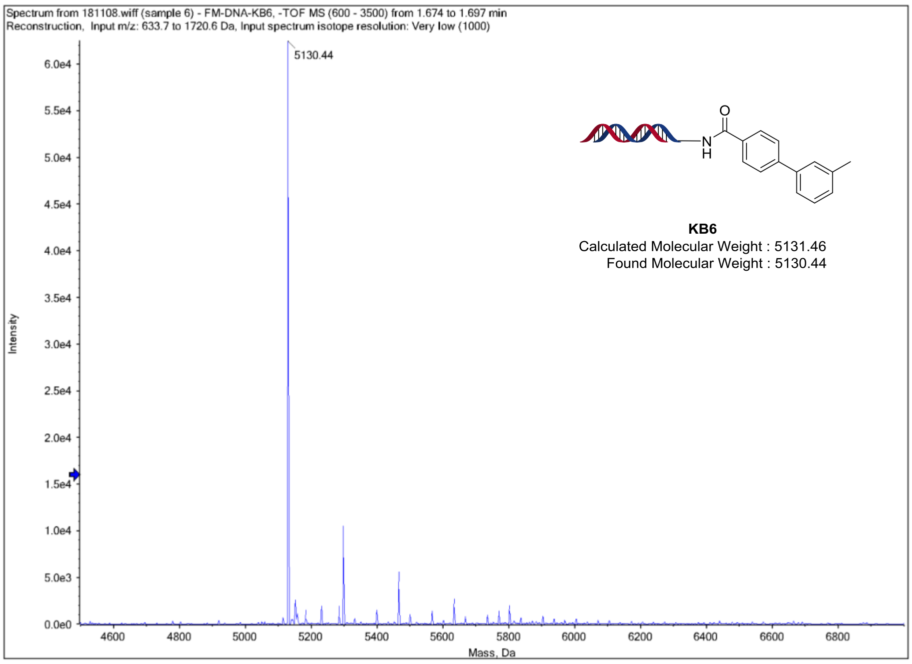
Spectrum from 181108.wiff (sample 6) - FM-DNA-KB6, -TOF MS (600 - 3500) from 1.674 to 1.697 min
Reconstruction, Input m/z: 633.7 to 1720.6 Da, Input spectrum isotope resolution: Very low (1000)
5130.44
Intensity
Mass, Da
KB6
Calculated Molecular Weight : 5131.46
Found Molecular Weight : 5130.44

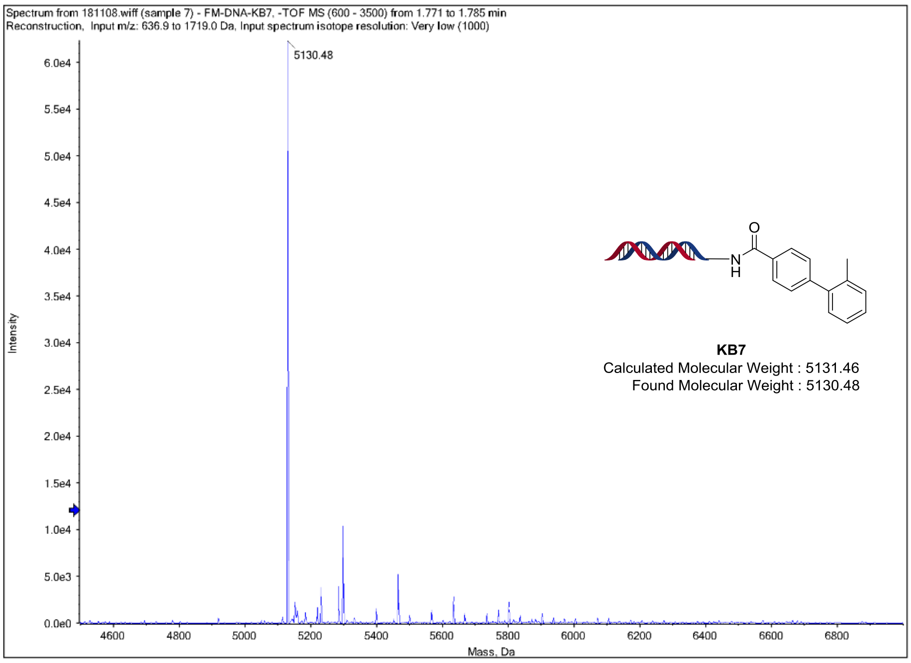
Spectrum from 181108.wiff (sample 7) - FM-DNA-KB7, -TOF MS (600 - 3500) from 1.771 to 1.785 min
Reconstruction, Input m/z: 636.9 to 1719.0 Da, Input spectrum isotope resolution: Very low (1000)
5130.48
Intensity
Mass, Da
KB7
Calculated Molecular Weight : 5131.46
Found Molecular Weight : 5130.48

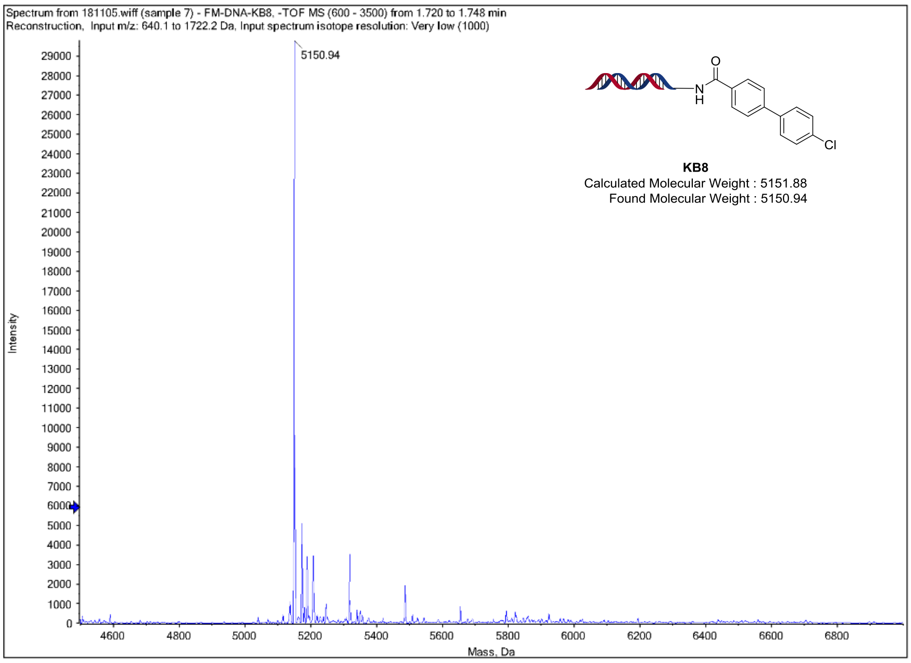
Spectrum from 181105.wiff (sample 7) - FM-DNA-KB8, -TOF MS (600 - 3500) from 1.720 to 1.748 min
Reconstruction, Input m/z: 640.1 to 1722.2 Da, Input spectrum isotope resolution: Very low (1000)
5150.94
KB8
Calculated Molecular Weight : 5151.88
Found Molecular Weight : 5150.94
Intensity
Mass, Da

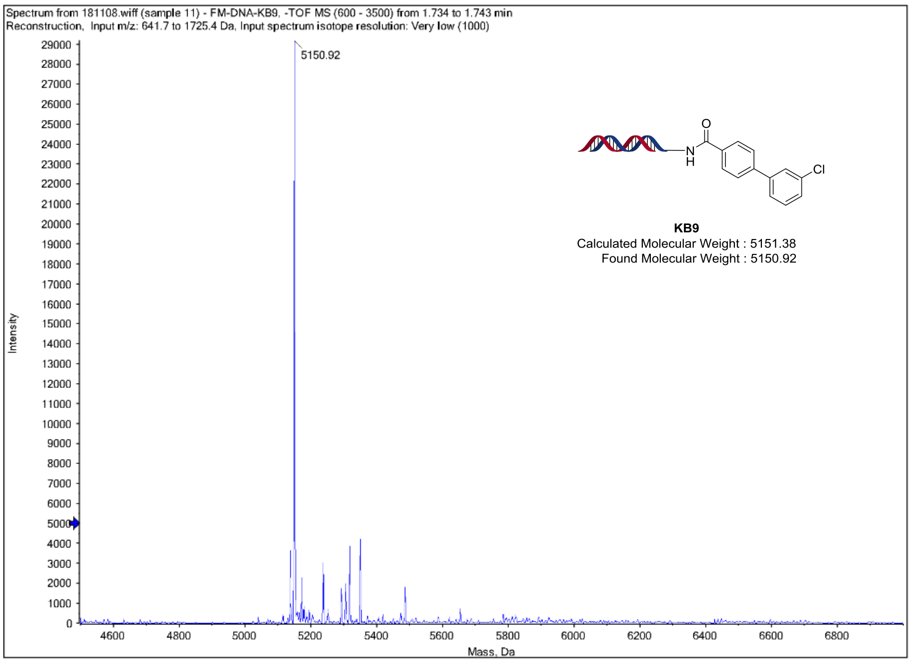
Spectrum from 181108.wiff (sample 11) - FM-DNA-KB9, -TOF MS (600 - 3500) from 1.734 to 1.743 min
Reconstruction, Input m/z: 641.7 to 1725.4 Da, Input spectrum isotope resolution: Very low (1000)
5150.92
KB9
Calculated Molecular Weight : 5151.38
Found Molecular Weight : 5150.92
Intensity
Mass, Da

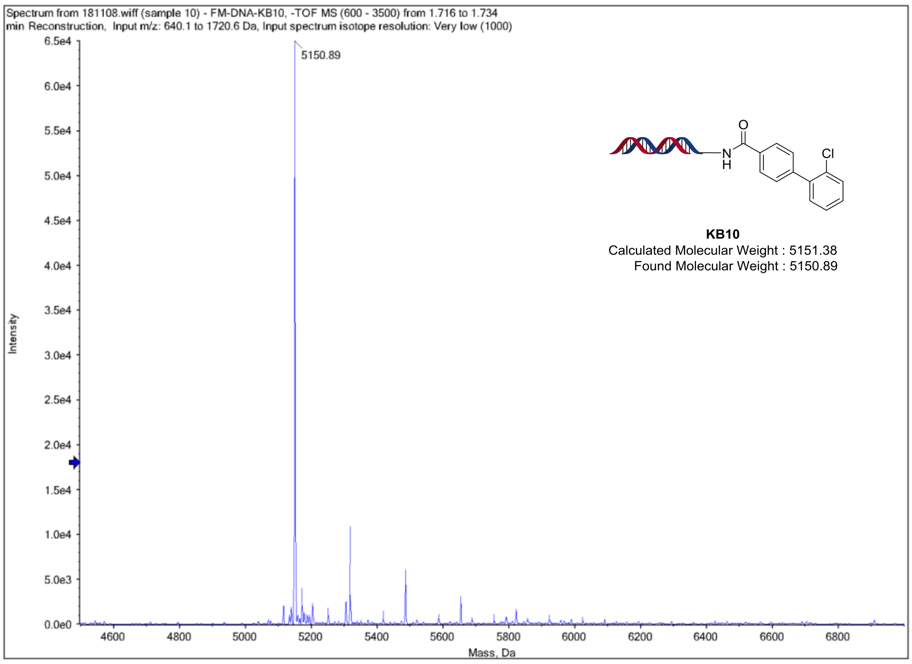
Spectrum from 181108.wiff (sample 10) - FM-DNA-KB10, -TOF MS (600 - 3500) from 1.716 to 1.734 min Reconstruction, Input m/z: 640.1 to 1720.6 Da, Input spectrum isotope resolution: Very low (1000)
5150.89
Intensity
Mass, Da
O
N
H
Cl
KB10
Calculated Molecular Weight : 5151.38
Found Molecular Weight : 5150.89

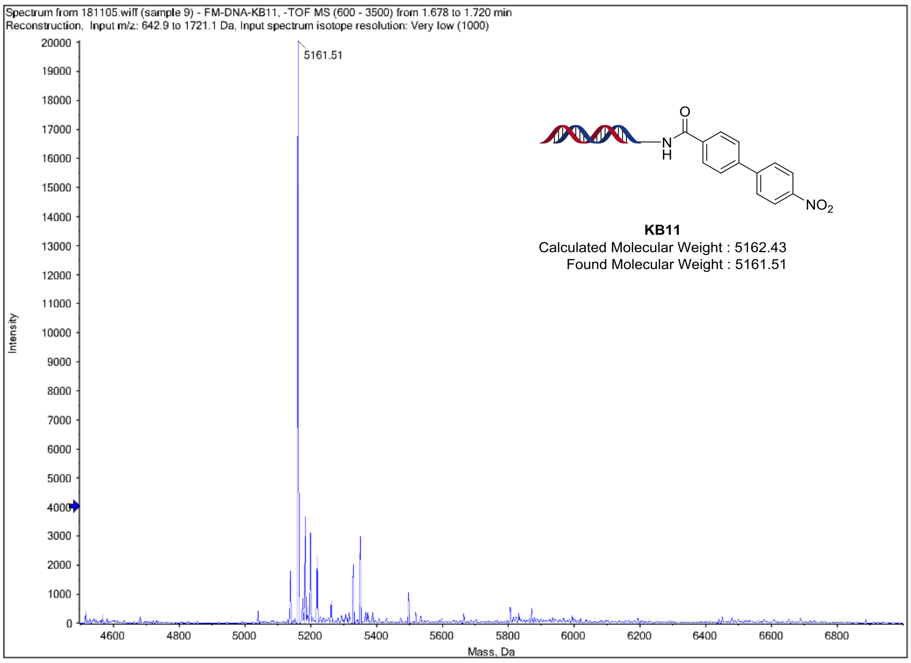
Spectrum from 181105.wiff (sample 9) - FM-DNA-KB11, -TOF MS (600 - 3500) from 1.678 to 1.720 min Reconstruction, Input m/z: 642.9 to 1721.1 Da, Input spectrum isotope resolution: Very low (1000)
5161.51
Intensity
Mass, Da
O
N
H
NO2
KB11
Calculated Molecular Weight : 5162.43
Found Molecular Weight : 5161.51

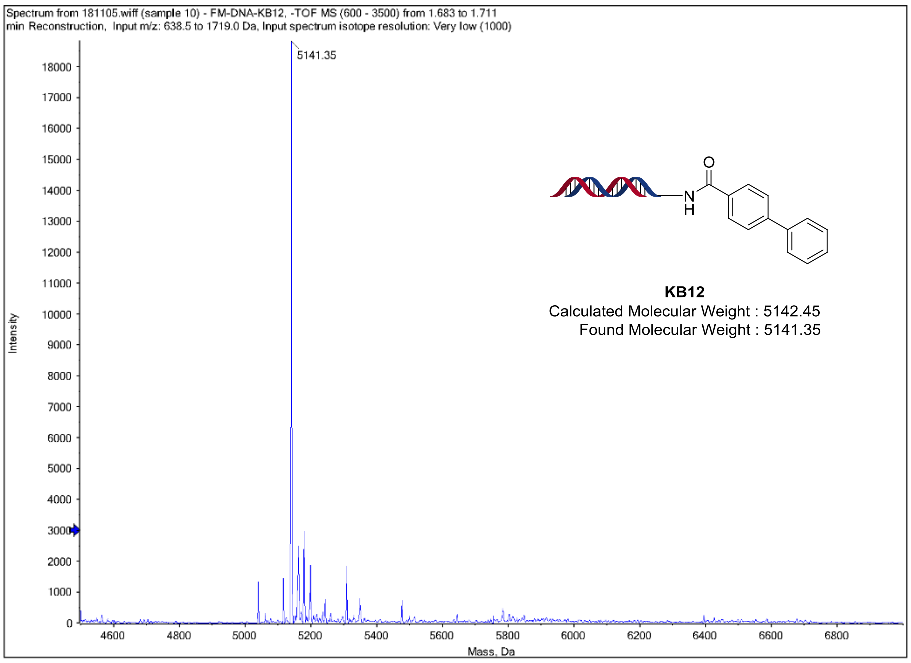
Spectrum from 181105.wiff (sample 10) - FM-DNA-KB12, -TOF MS (600 - 3500) from 1.683 to 1.711 min Reconstruction, Input m/z: 638.5 to 1719.0 Da, Input spectrum isotope resolution: Very low (1000)
5141.35
Intensity
Mass, Da
O
N
H
KB12
Calculated Molecular Weight : 5142.45
Found Molecular Weight : 5141.35

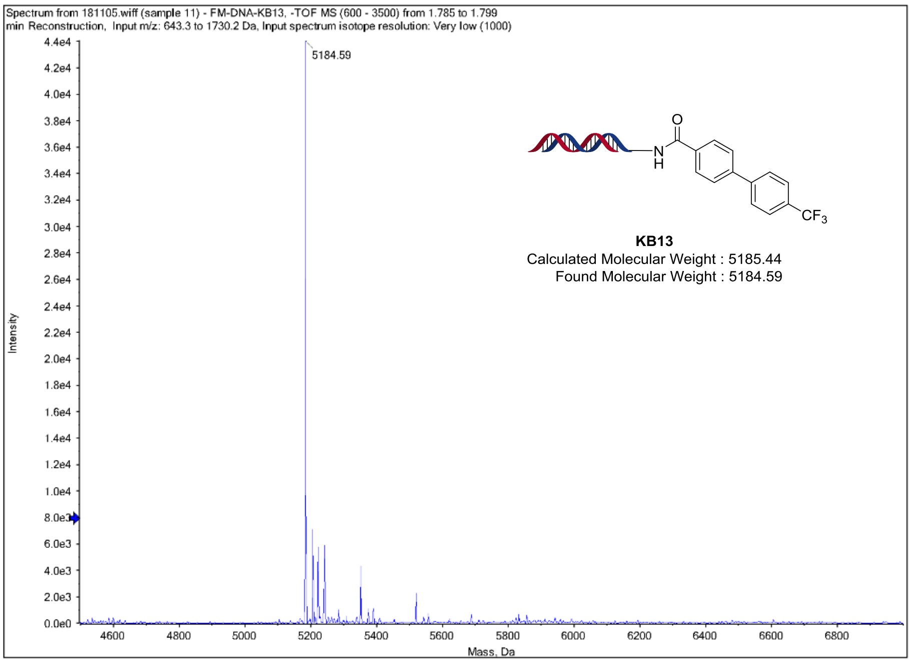
Spectrum from 181105.wiff (sample 11) - FM-DNA-KB13, -TOF MS (600 - 3500) from 1.785 to 1.799 min Reconstruction, Input m/z: 643.3 to 1730.2 Da, Input spectrum isotope resolution: Very low (1000)
5184.59
Intensity
Mass, Da
O
N
H
CF3
KB13
Calculated Molecular Weight : 5185.44
Found Molecular Weight : 5184.59

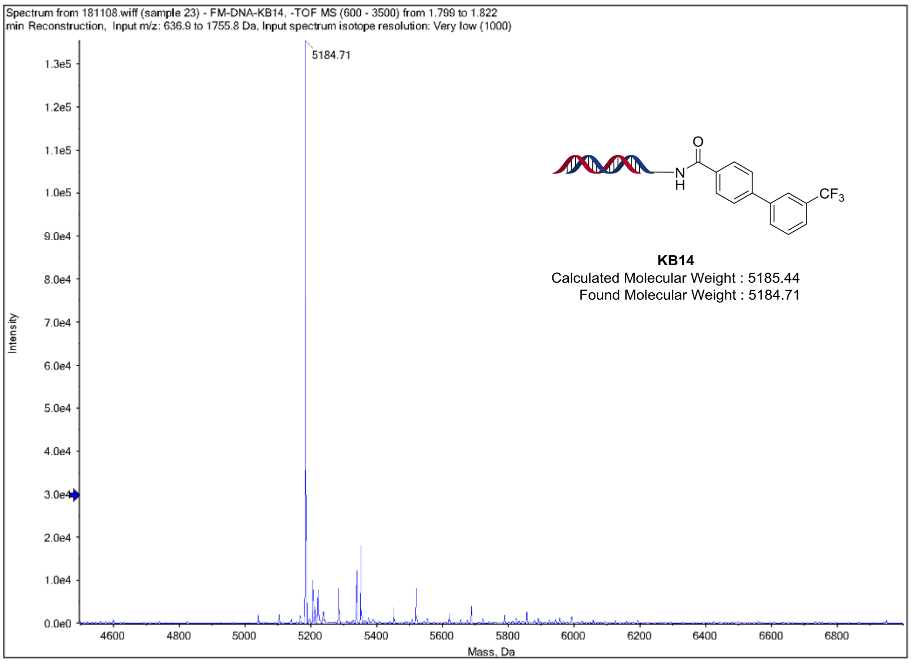
Spectrum from 181108.wiff (sample 23) - FM-DNA-KB14, -TOF MS (600 - 3500) from 1.799 to 1.822 min Reconstruction, Input m/z: 636.9 to 1755.8 Da, Input spectrum isotope resolution: Very low (1000)
5184.71
Intensity
Mass, Da
O
N
H
CF3
KB14
Calculated Molecular Weight : 5185.44
Found Molecular Weight : 5184.71

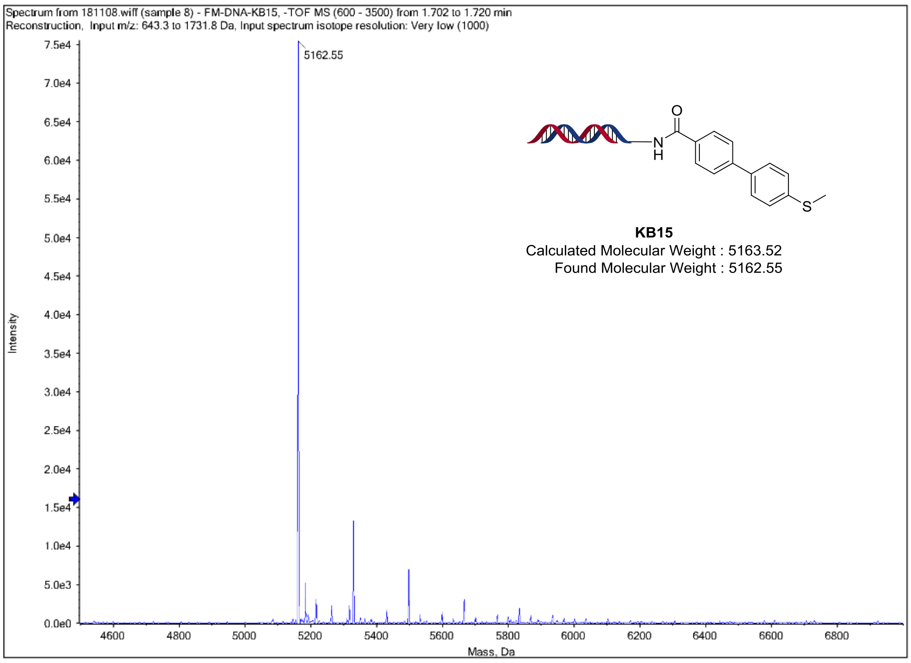
Spectrum from 181108.wiff (sample 8) - FM-DNA-KB15, -TOF MS (600 - 3500) from 1.702 to 1.720 min Reconstruction, Input m/z: 643.3 to 1731.8 Da, Input spectrum isotope resolution: Very low (1000)
5162.55
Intensity
Mass, Da
O
N
H
S
KB15
Calculated Molecular Weight : 5163.52
Found Molecular Weight : 5162.55

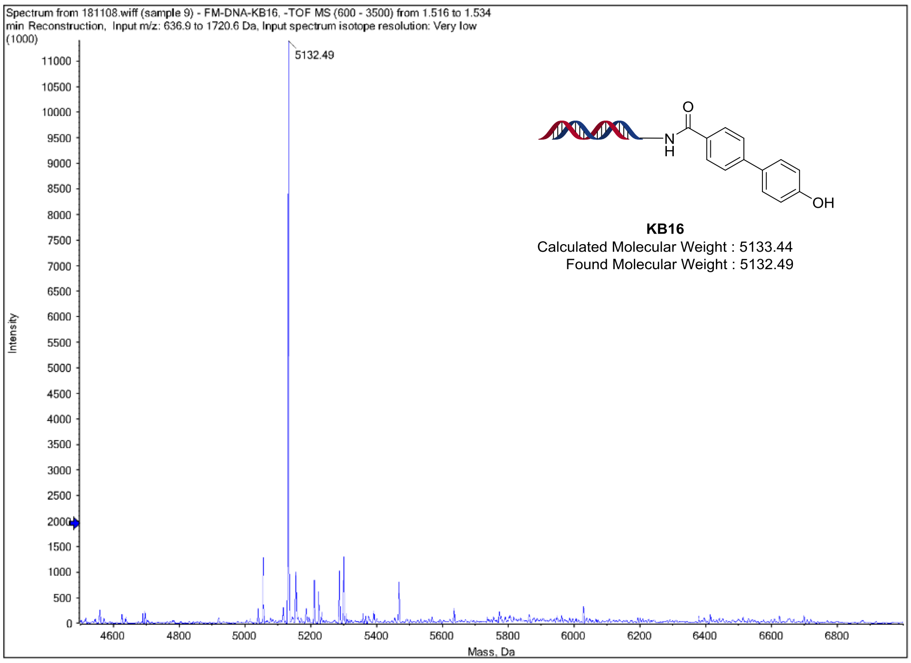

Spectrum from 181108.wiff (sample 9) - FM-DNA-KB16, -TOF MS (600 - 3500) from 1.516 to 1.534 min Reconstruction, Input m/z: 636.9 to 1720.6 Da, Input spectrum isotope resolution: Very low (1000)
5132.49
Intensity
Mass, Da
KB16
Calculated Molecular Weight : 5133.44
Found Molecular Weight : 5132.49

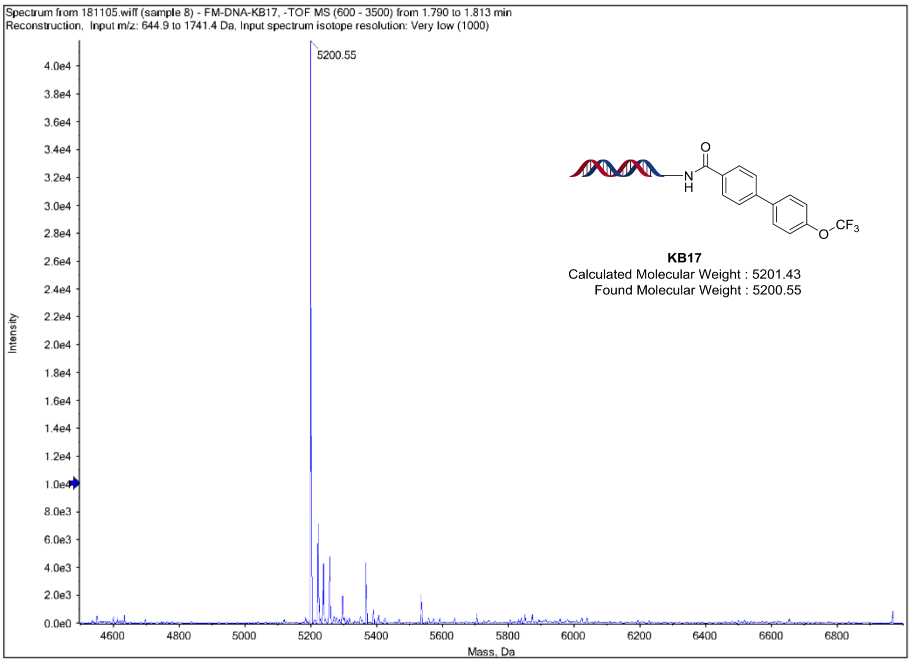

Spectrum from 181105.wiff (sample 8) - FM-DNA-KB17, -TOF MS (600 - 3500) from 1.790 to 1.813 min Reconstruction, Input m/z: 644.9 to 1741.4 Da, Input spectrum isotope resolution: Very low (1000)
5200.55
Intensity
Mass, Da
KB17
Calculated Molecular Weight : 5201.43
Found Molecular Weight : 5200.55

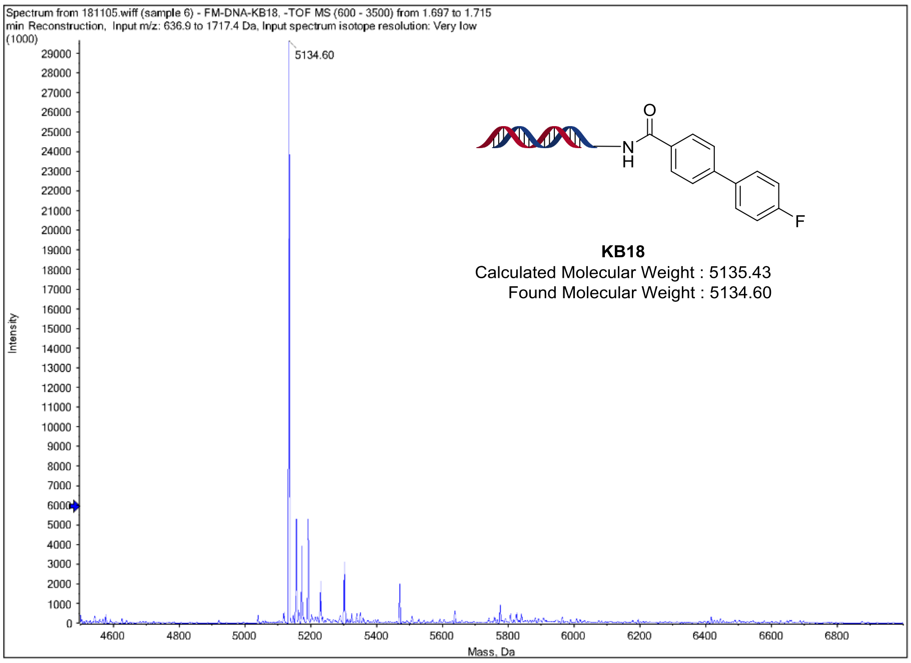
Spectrum from 181105.wiff (sample 6) - FM-DNA-KB18, -TOF MS (600 - 3500) from 1.697 to 1.715 min Reconstruction, Input m/z: 636.9 to 1717.4 Da, Input spectrum isotope resolution: Very low (1000)
5134.60
KB18
Calculated Molecular Weight : 5135.43
Found Molecular Weight : 5134.60
Intensity
Mass, Da

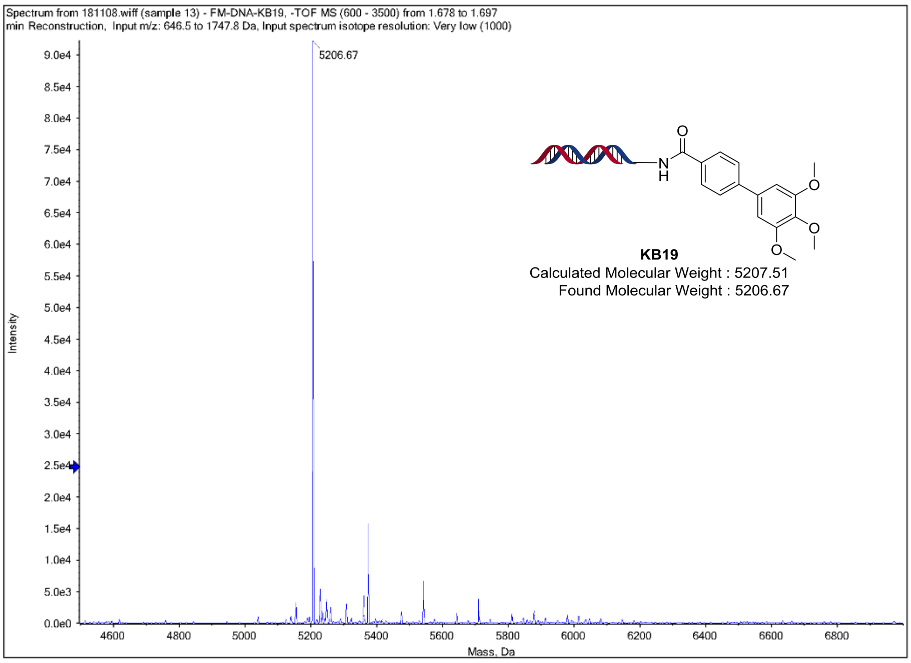
Spectrum from 181108.wiff (sample 13) - FM-DNA-KB19, -TOF MS (600 - 3500) from 1.678 to 1.697 min Reconstruction, Input m/z: 646.5 to 1747.8 Da, Input spectrum isotope resolution: Very low (1000)
5206.67
KB19
Calculated Molecular Weight : 5207.51
Found Molecular Weight : 5206.67
Intensity
Mass, Da

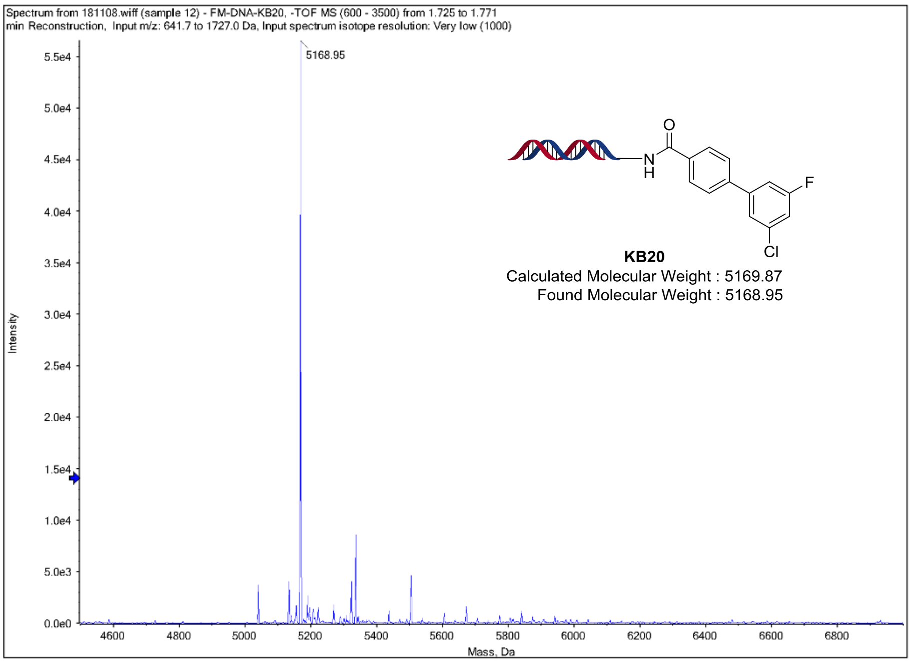
Spectrum from 181108.wiff (sample 12) - FM-DNA-KB20, -TOF MS (600 - 3500) from 1.725 to 1.771 min Reconstruction, Input m/z: 641.7 to 1727.0 Da, Input spectrum isotope resolution: Very low (1000)
5168.95
Intensity
Mass, Da
O
N
H
F
Cl
KB20
Calculated Molecular Weight : 5169.87
Found Molecular Weight : 5168.95

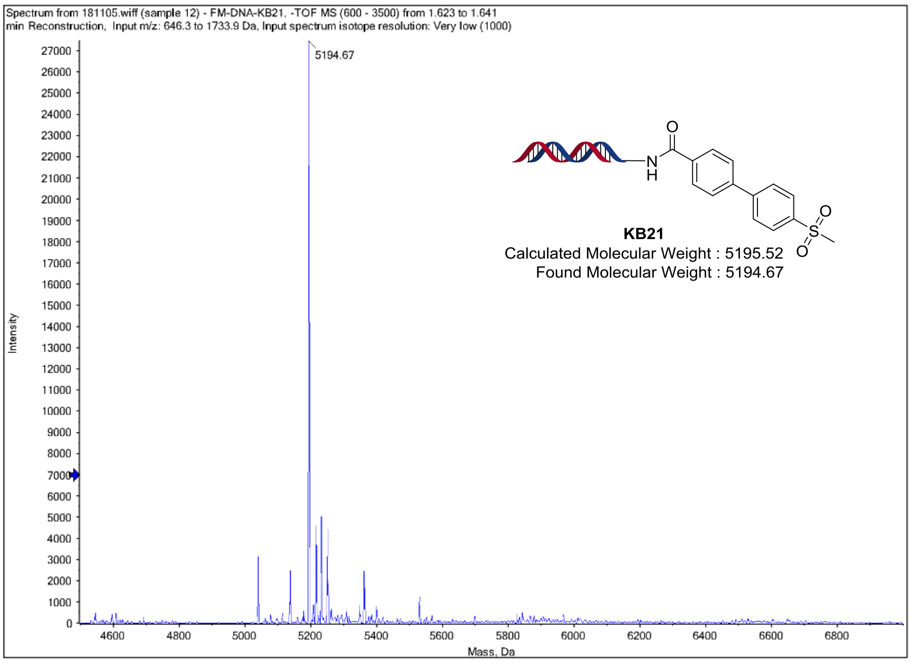
Spectrum from 181105.wiff (sample 12) - FM-DNA-KB21, -TOF MS (600 - 3500) from 1.623 to 1.641 min Reconstruction, Input m/z: 646.3 to 1733.9 Da, Input spectrum isotope resolution: Very low (1000)
5194.67
Intensity
Mass, Da
O
N
H
S
O
O
KB21
Calculated Molecular Weight : 5195.52
Found Molecular Weight : 5194.67

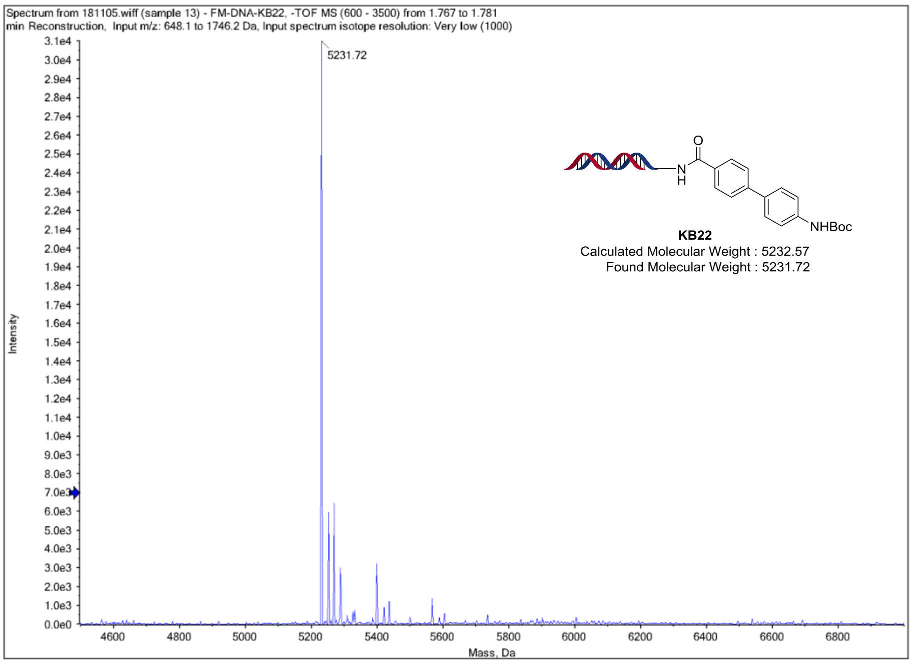
Spectrum from 181105.wiff (sample 13) - FM-DNA-KB22, -TOF MS (600 - 3500) from 1.767 to 1.781 min Reconstruction, Input m/z: 648.1 to 1746.2 Da, Input spectrum isotope resolution: Very low (1000)
5231.72
Intensity
Mass, Da
O
N
H
NHBoc
KB22
Calculated Molecular Weight : 5232.57
Found Molecular Weight : 5231.72

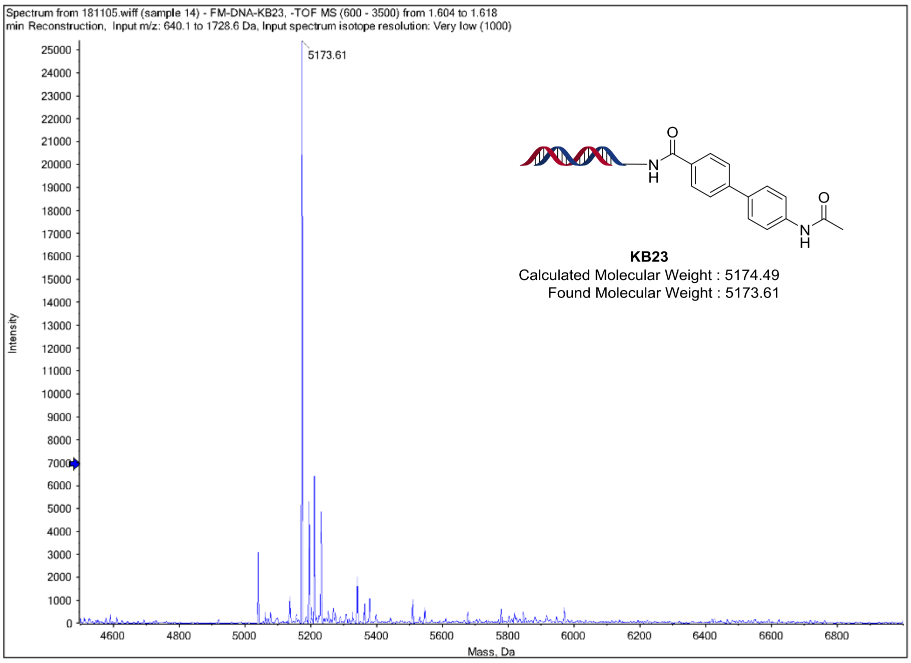
Spectrum from 181105.wiff (sample 14) - FM-DNA-KB23, -TOF MS (600 - 3500) from 1.604 to 1.618 min Reconstruction, Input m/z: 640.1 to 1728.6 Da, Input spectrum isotope resolution: Very low (1000)
5173.61
Intensity
Mass, Da
O
N
H
O
N
H
KB23
Calculated Molecular Weight : 5174.49
Found Molecular Weight : 5173.61

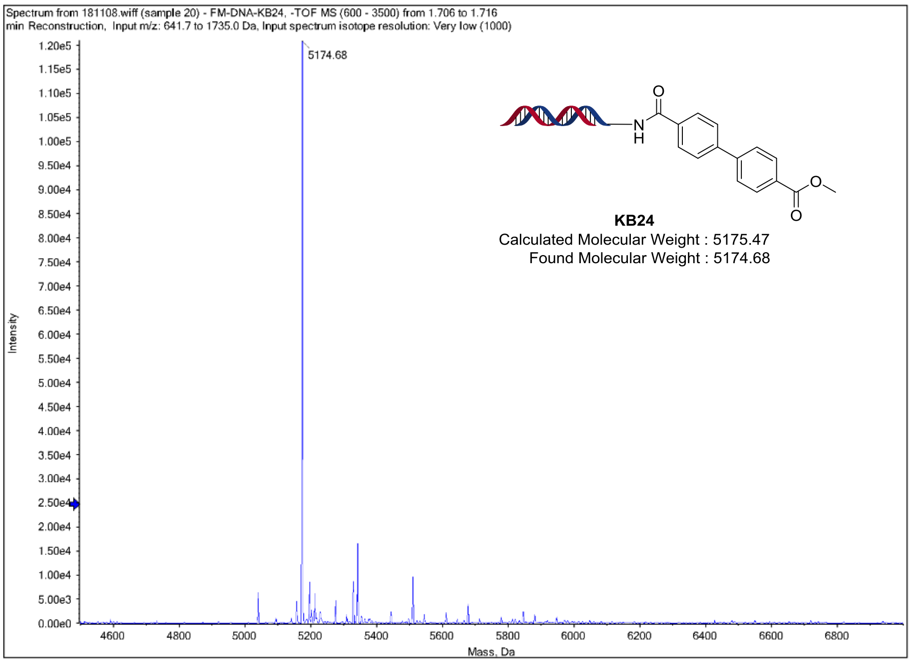

Spectrum from 181108.wiff (sample 20) - FM-DNA-KB24, -TOF MS (600 - 3500) from 1.706 to 1.716 min Reconstruction, Input m/z: 641.7 to 1735.0 Da, Input spectrum isotope resolution: Very low (1000)
5174.68
KB24
Calculated Molecular Weight : 5175.47
Found Molecular Weight : 5174.68
Intensity
Mass, Da

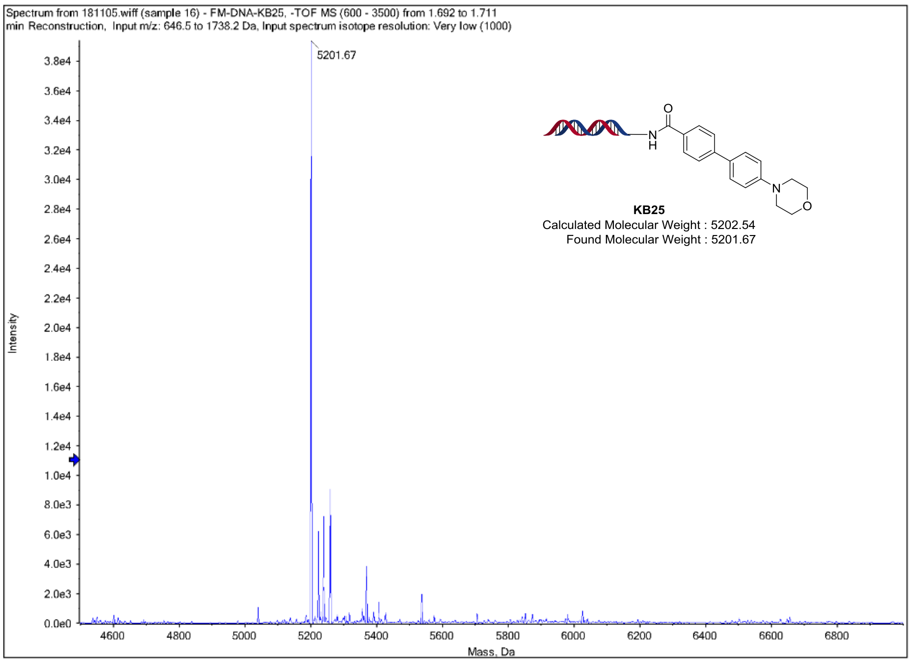

Spectrum from 181105.wiff (sample 16) - FM-DNA-KB25, -TOF MS (600 - 3500) from 1.692 to 1.711 min Reconstruction, Input m/z: 646.5 to 1738.2 Da, Input spectrum isotope resolution: Very low (1000)
5201.67
KB25
Calculated Molecular Weight : 5202.54
Found Molecular Weight : 5201.67
Intensity
Mass, Da

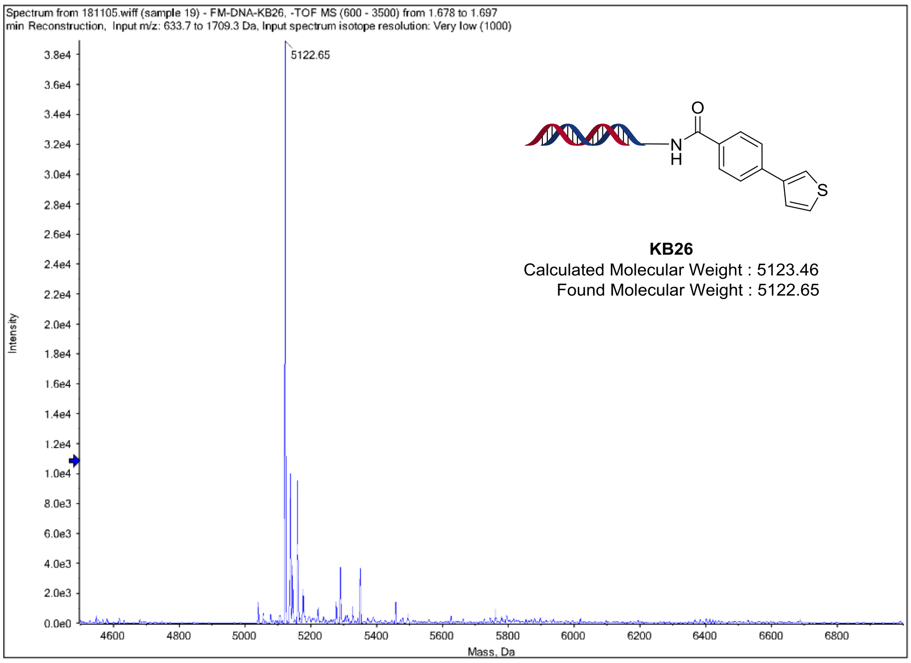
Spectrum from 181105.wiff (sample 19) - FM-DNA-KB26, -TOF MS (600 - 3500) from 1.678 to 1.697 min Reconstruction, Input m/z: 633.7 to 1709.3 Da, Input spectrum isotope resolution: Very low (1000)
5122.65
Intensity
Mass, Da
KB26
Calculated Molecular Weight : 5123.46
Found Molecular Weight : 5122.65

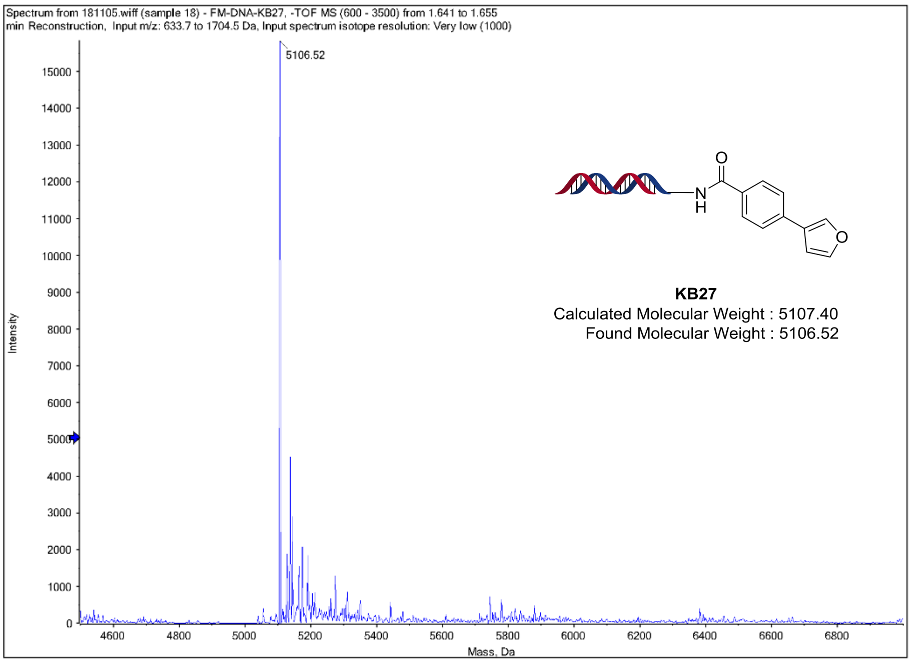
Spectrum from 181105.wiff (sample 18) - FM-DNA-KB27, -TOF MS (600 - 3500) from 1.641 to 1.655 min Reconstruction, Input m/z: 633.7 to 1704.5 Da, Input spectrum isotope resolution: Very low (1000)
5106.52
Intensity
Mass, Da
KB27
Calculated Molecular Weight : 5107.40
Found Molecular Weight : 5106.52

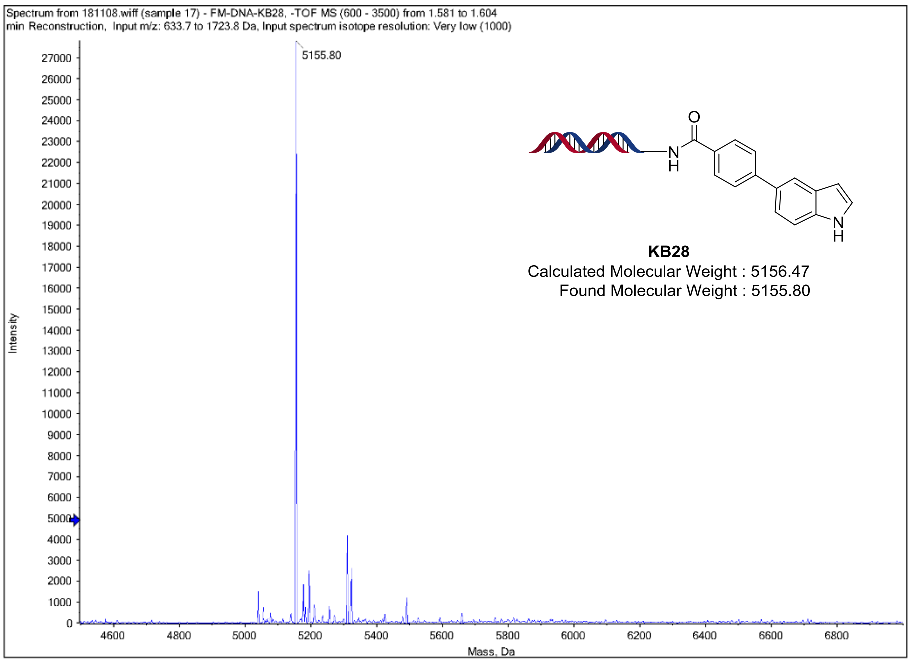
Spectrum from 181108.wiff (sample 17) - FM-DNA-KB28, -TOF MS (600 - 3500) from 1.581 to 1.604 min Reconstruction, Input m/z: 633.7 to 1723.8 Da, Input spectrum isotope resolution: Very low (1000)
5155.80
Intensity
Mass, Da
KB28
Calculated Molecular Weight : 5156.47
Found Molecular Weight : 5155.80

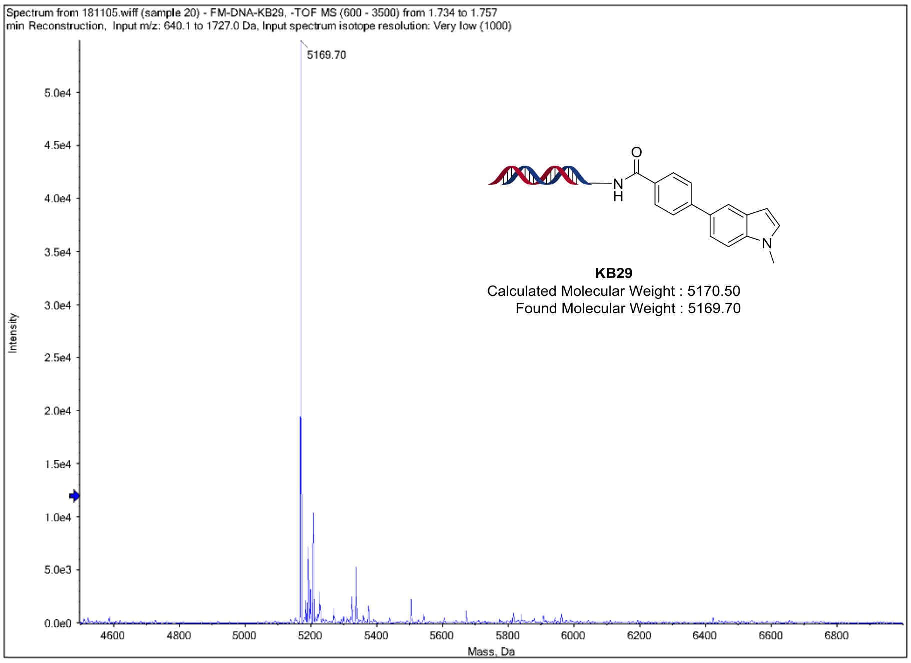
Spectrum from 181105.wiff (sample 20) - FM-DNA-KB29, -TOF MS (600 - 3500) from 1.734 to 1.757 min Reconstruction, Input m/z: 640.1 to 1727.0 Da, Input spectrum isotope resolution: Very low (1000)
5169.70
Intensity
Mass, Da
KB29
Calculated Molecular Weight : 5170.50
Found Molecular Weight : 5169.70

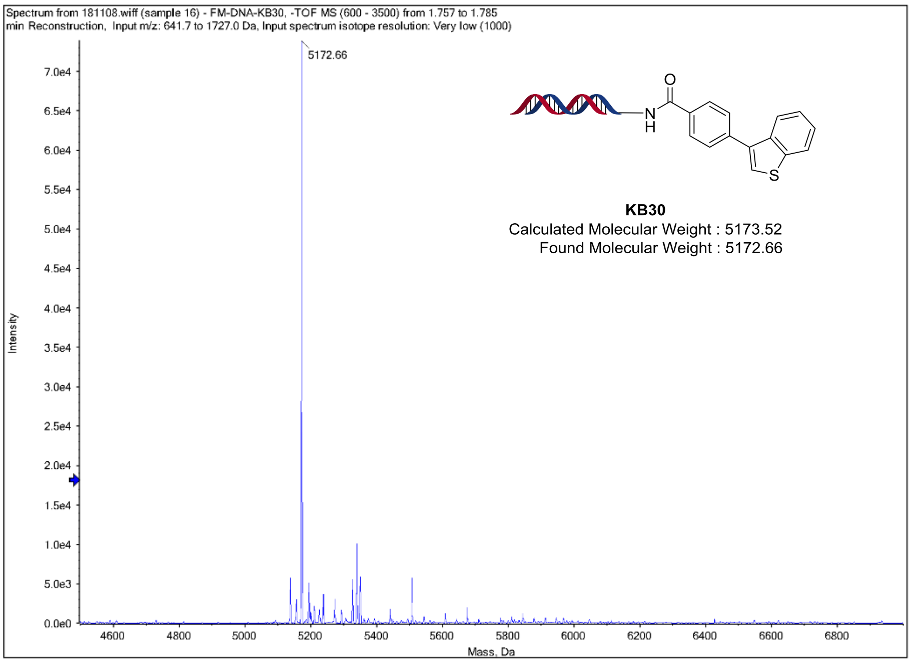

Spectrum from 181108.wiff (sample 16) - FM-DNA-KB30, -TOF MS (600 - 3500) from 1.757 to 1.785 min Reconstruction, Input m/z: 641.7 to 1727.0 Da, Input spectrum isotope resolution: Very low (1000)
5172.66
Intensity
Mass, Da
KB30
Calculated Molecular Weight : 5173.52
Found Molecular Weight : 5172.66

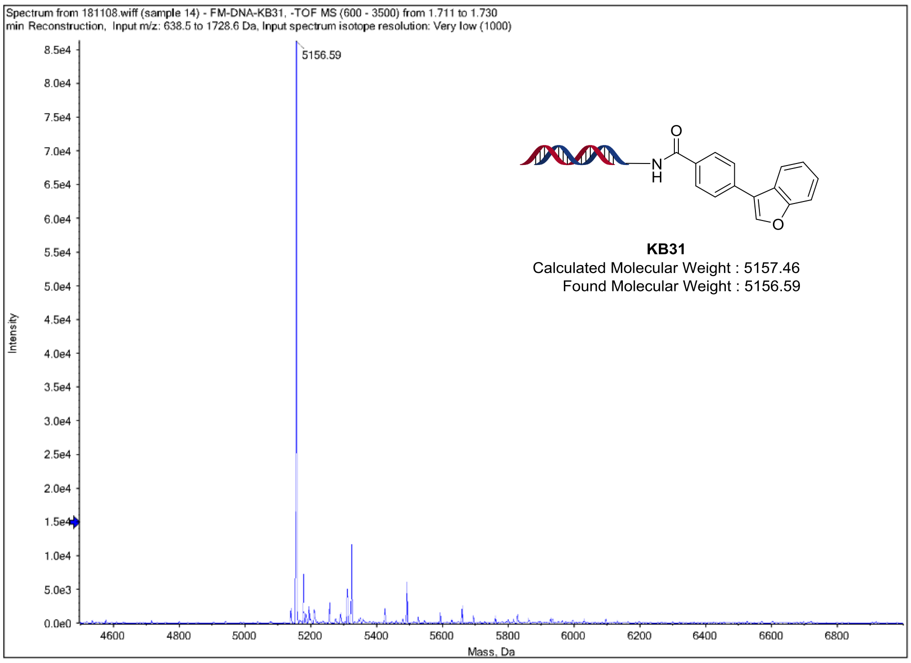

Spectrum from 181108.wiff (sample 14) - FM-DNA-KB31, -TOF MS (600 - 3500) from 1.711 to 1.730 min Reconstruction, Input m/z: 638.5 to 1728.6 Da, Input spectrum isotope resolution: Very low (1000)
5156.59
Intensity
Mass, Da
KB31
Calculated Molecular Weight : 5157.46
Found Molecular Weight : 5156.59

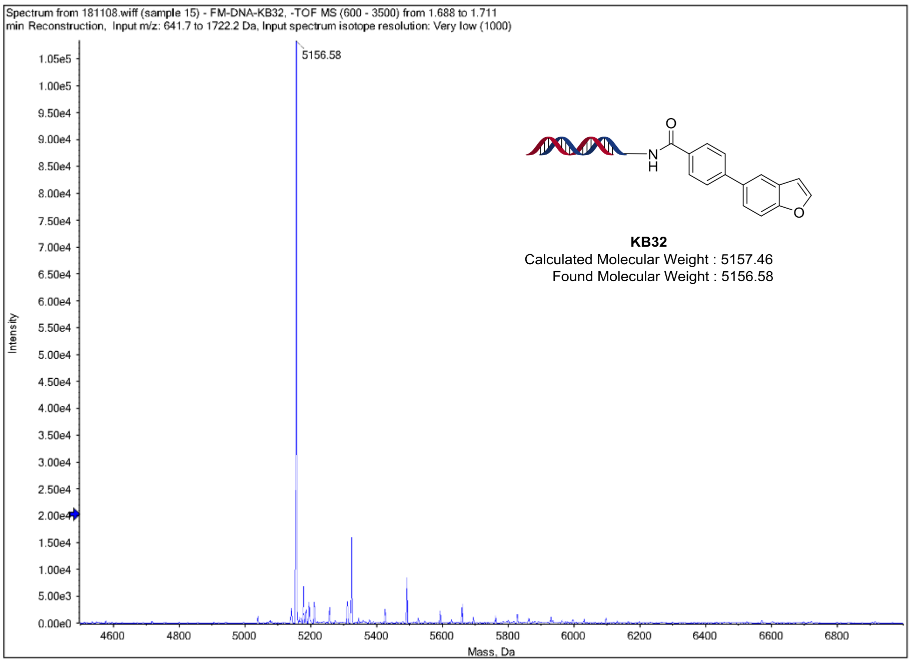
Spectrum from 181108.wiff (sample 15) - FM-DNA-KB32, -TOF MS (600 - 3500) from 1.688 to 1.711 min Reconstruction, Input m/z: 641.7 to 1722.2 Da, Input spectrum isotope resolution: Very low (1000)
5156.58
Intensity
Mass, Da
KB32
Calculated Molecular Weight : 5157.46
Found Molecular Weight : 5156.58

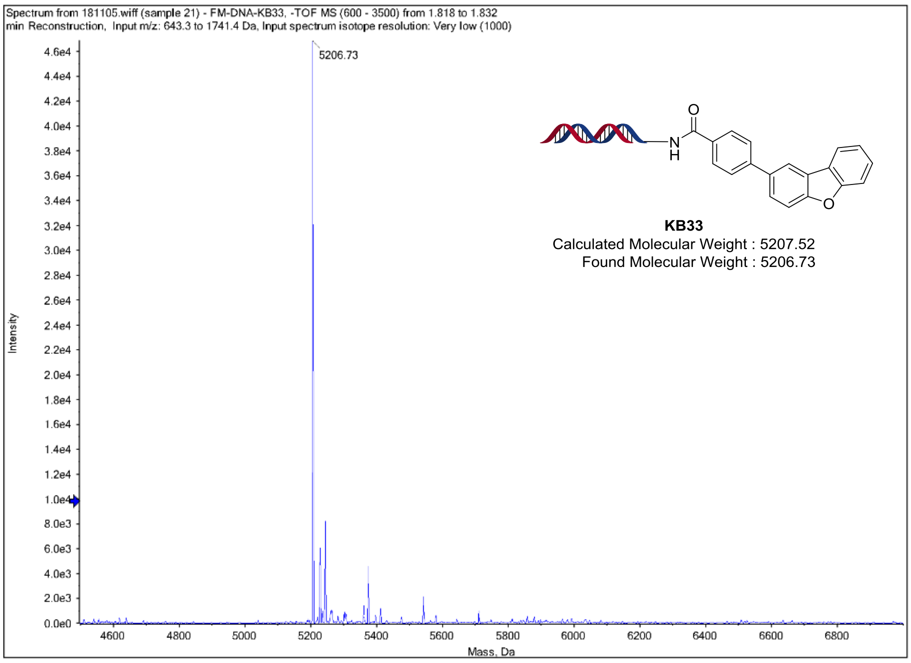
Spectrum from 181105.wiff (sample 21) - FM-DNA-KB33, -TOF MS (600 - 3500) from 1.818 to 1.832 min Reconstruction, Input m/z: 643.3 to 1741.4 Da, Input spectrum isotope resolution: Very low (1000)
5206.73
Intensity
Mass, Da
KB33
Calculated Molecular Weight : 5207.52
Found Molecular Weight : 5206.73

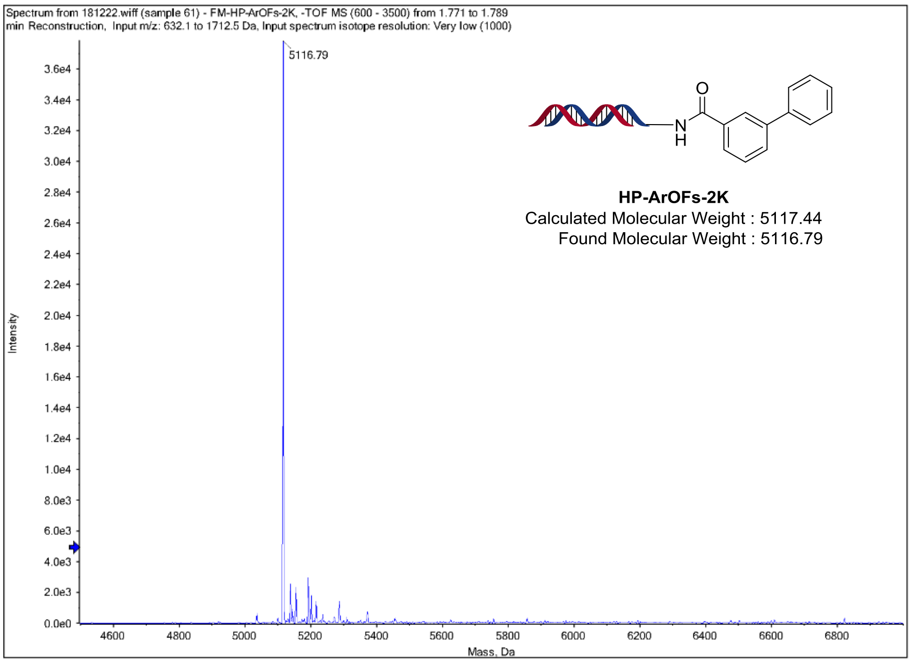
Spectrum from 181222.wiff (sample 61) - FM-HP-ArOFs-2K, -TOF MS (600 - 3500) from 1.771 to 1.789 min Reconstruction, Input m/z: 632.1 to 1712.5 Da, Input spectrum isotope resolution: Very low (1000)
5116.79
Intensity
Mass, Da
HP-ArOFs-2K
Calculated Molecular Weight : 5117.44
Found Molecular Weight : 5116.79

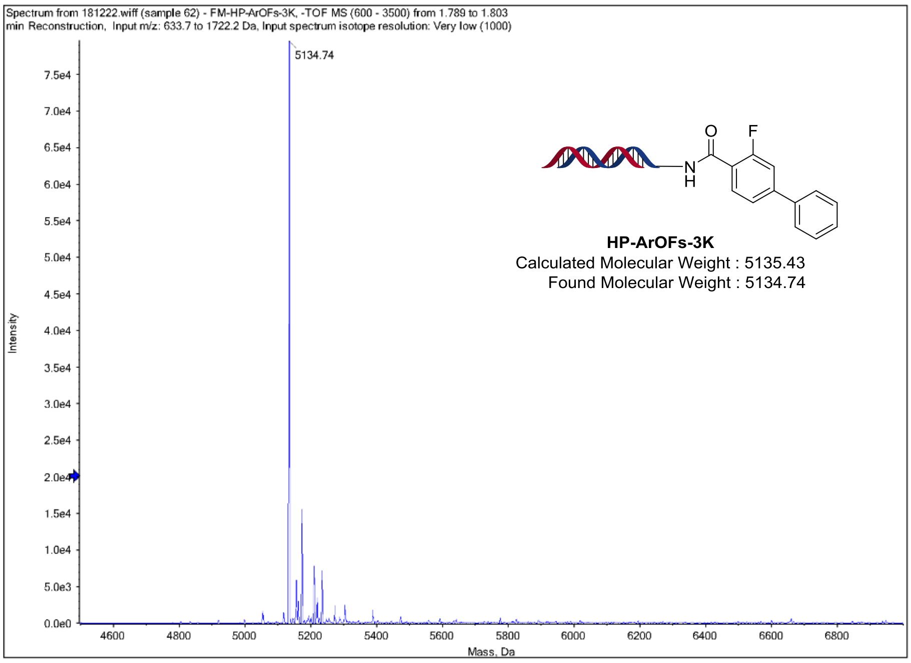
Spectrum from 181222.wiff (sample 62) - FM-HP-ArOFs-3K, -TOF MS (600 - 3500) from 1.789 to 1.803 min Reconstruction, Input m/z: 633.7 to 1722.2 Da, Input spectrum isotope resolution: Very low (1000)
5134.74
Intensity
Mass, Da
HP-ArOFs-3K
Calculated Molecular Weight : 5135.43
Found Molecular Weight : 5134.74

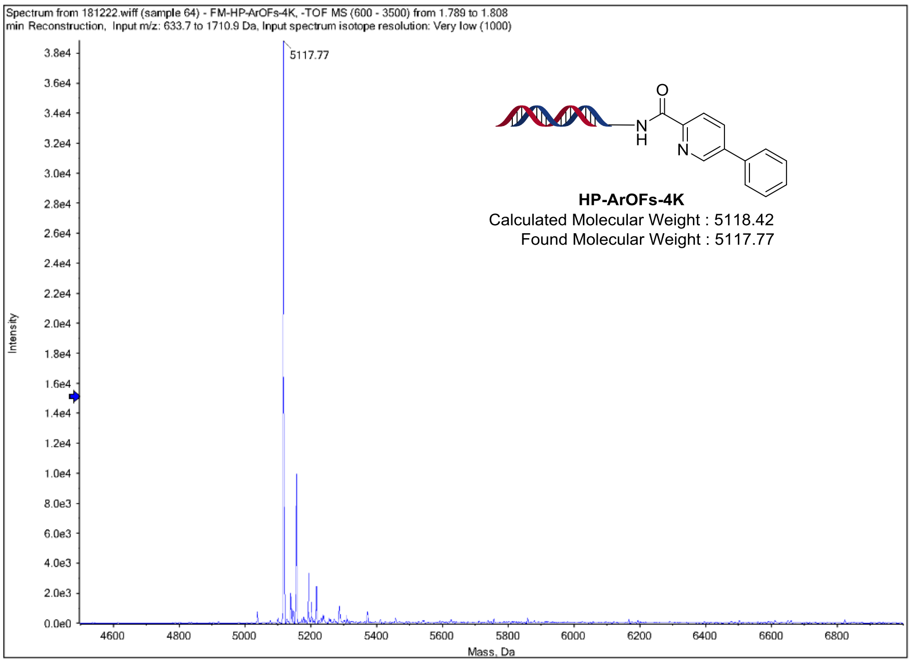
Spectrum from 181222.wiff (sample 64) - FM-HP-ArOFs-4K, -TOF MS (600 - 3500) from 1.789 to 1.808 min Reconstruction, Input m/z: 633.7 to 1710.9 Da, Input spectrum isotope resolution: Very low (1000)
5117.77
HP-ArOFs-4K
Calculated Molecular Weight : 5118.42
Found Molecular Weight : 5117.77
Intensity
Mass, Da

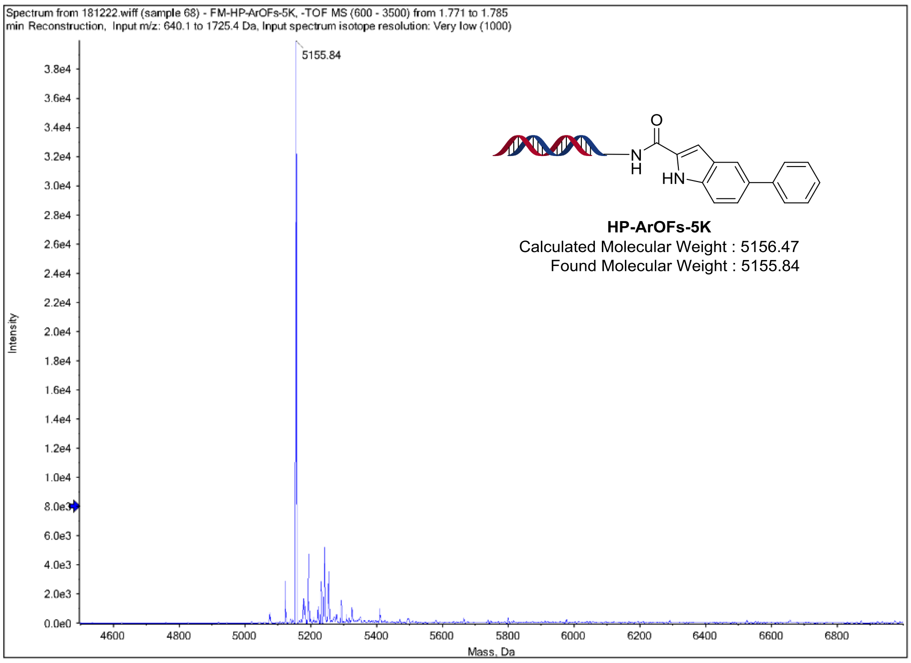
Spectrum from 181222.wiff (sample 68) - FM-HP-ArOFs-5K, -TOF MS (600 - 3500) from 1.771 to 1.785 min Reconstruction, Input m/z: 640.1 to 1725.4 Da, Input spectrum isotope resolution: Very low (1000)
5155.84
HP-ArOFs-5K
Calculated Molecular Weight : 5156.47
Found Molecular Weight : 5155.84
Intensity
Mass, Da

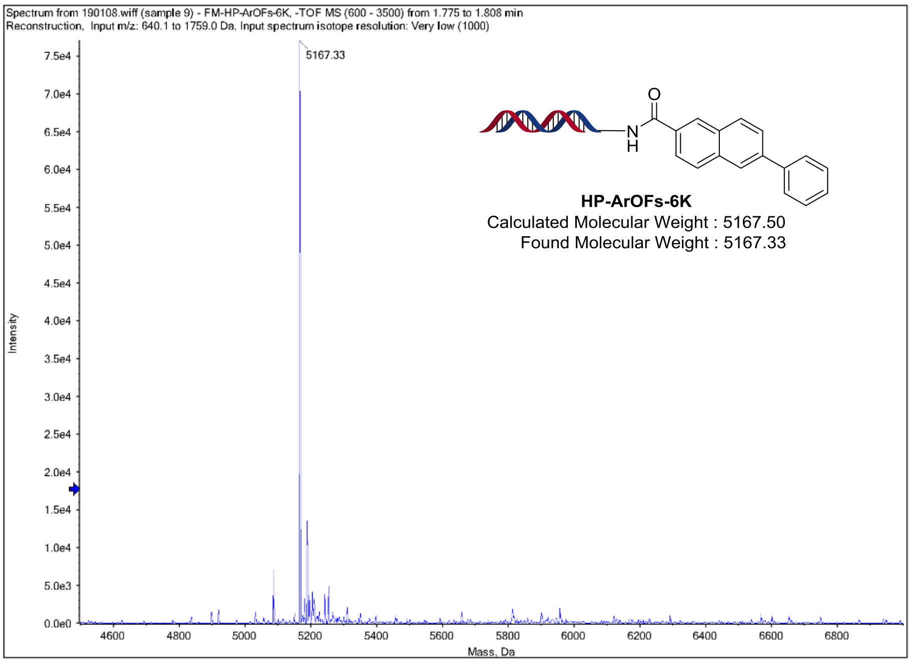

Spectrum from 190108.wiff (sample 9) - FM-HP-ArOFs-6K, -TOF MS (600 - 3500) from 1.775 to 1.808 min
Reconstruction, Input m/z: 640.1 to 1759.0 Da, Input spectrum isotope resolution: Very low (1000)
5167.33
HP-ArOFs-6K
Calculated Molecular Weight : 5167.50
Found Molecular Weight : 5167.33
Intensity
Mass, Da

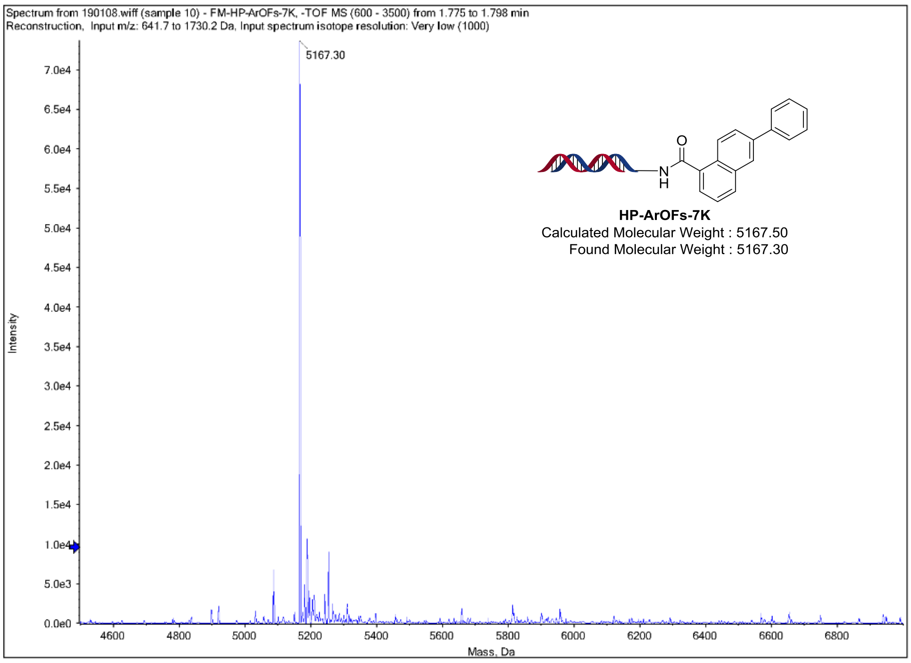

Spectrum from 190108.wiff (sample 10) - FM-HP-ArOFs-7K, -TOF MS (600 - 3500) from 1.775 to 1.798 min
Reconstruction, Input m/z: 641.7 to 1730.2 Da, Input spectrum isotope resolution: Very low (1000)
5167.30
HP-ArOFs-7K
Calculated Molecular Weight : 5167.50
Found Molecular Weight : 5167.30
Intensity
Mass, Da

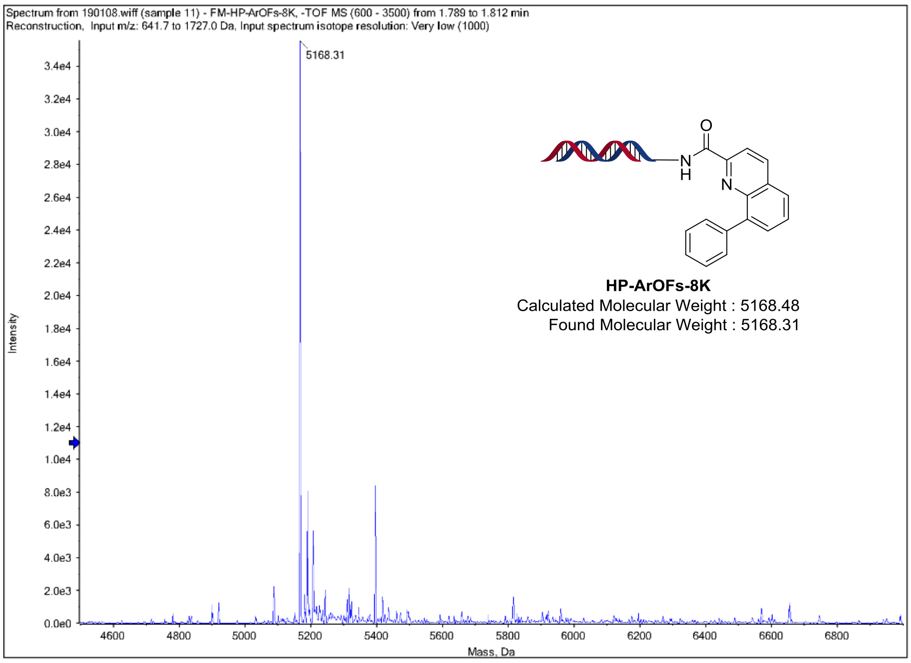
Spectrum from 190108.wiff (sample 11) - FM-HP-ArOFs-8K, -TOF MS (600 - 3500) from 1.789 to 1.812 min
Reconstruction, Input m/z: 641.7 to 1727.0 Da, Input spectrum isotope resolution: Very low (1000)
5168.31
Intensity
Mass, Da
O
N
H
N
HP-ArOFs-8K
Calculated Molecular Weight : 5168.48
Found Molecular Weight : 5168.31

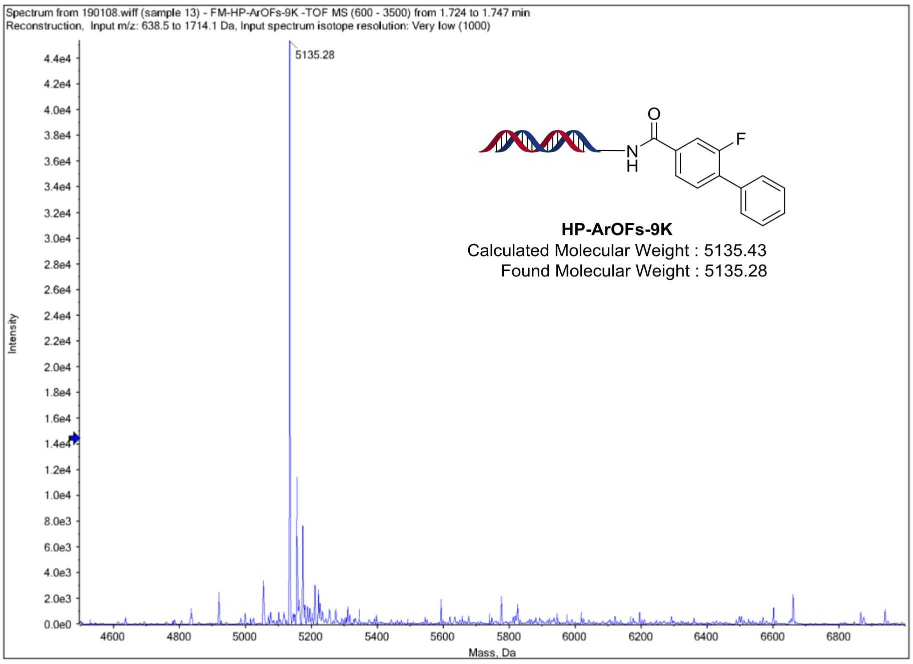
Spectrum from 190108.wiff (sample 13) - FM-HP-ArOFs-9K -TOF MS (600 - 3500) from 1.724 to 1.747 min
Reconstruction, Input m/z: 638.5 to 1714.1 Da, Input spectrum isotope resolution: Very low (1000)
5135.28
Intensity
Mass, Da
O
F
N
H
HP-ArOFs-9K
Calculated Molecular Weight : 5135.43
Found Molecular Weight : 5135.28

MS Spectra of On-DNA Sonogashira coupling products

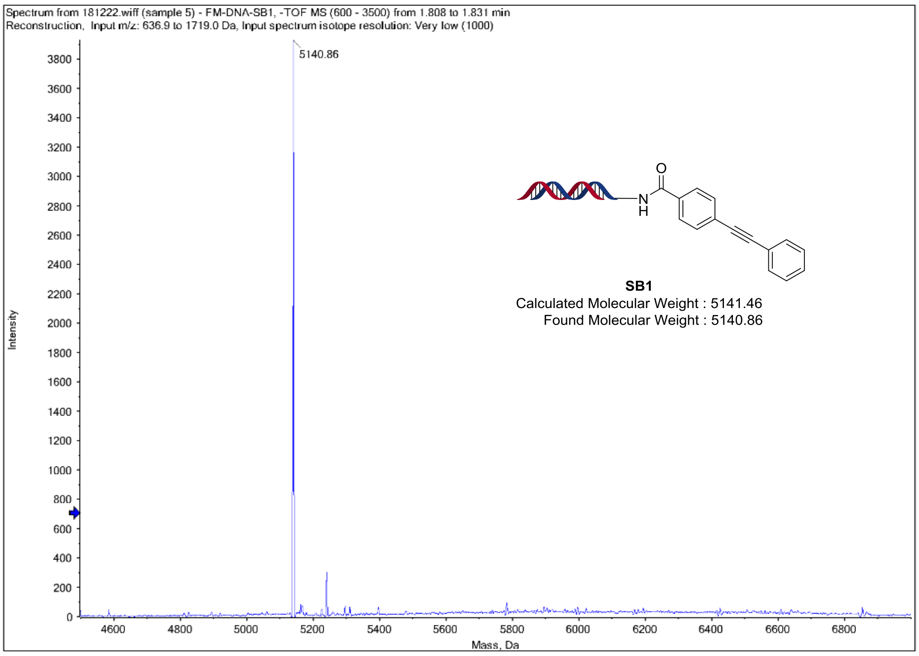

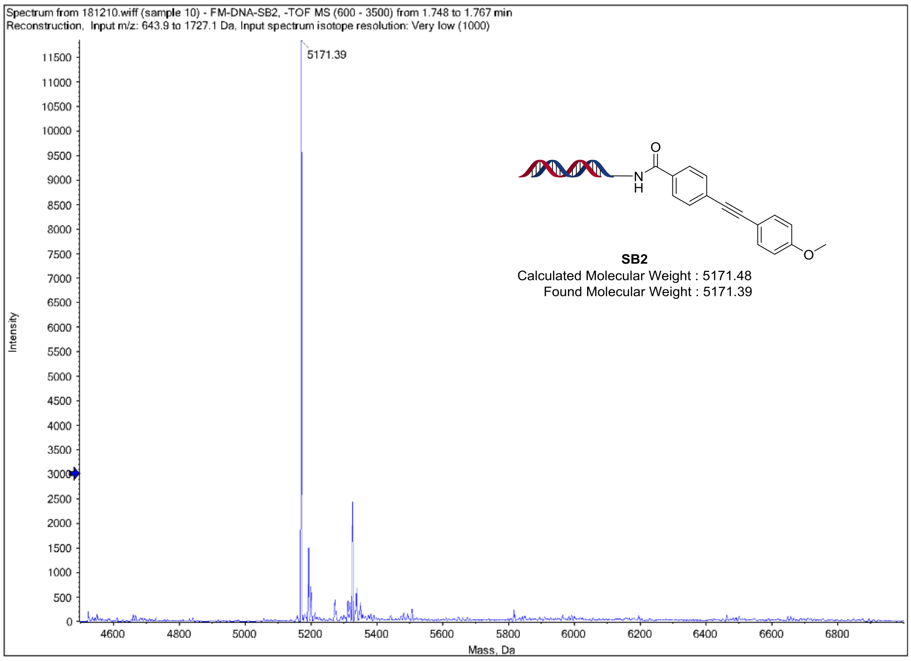

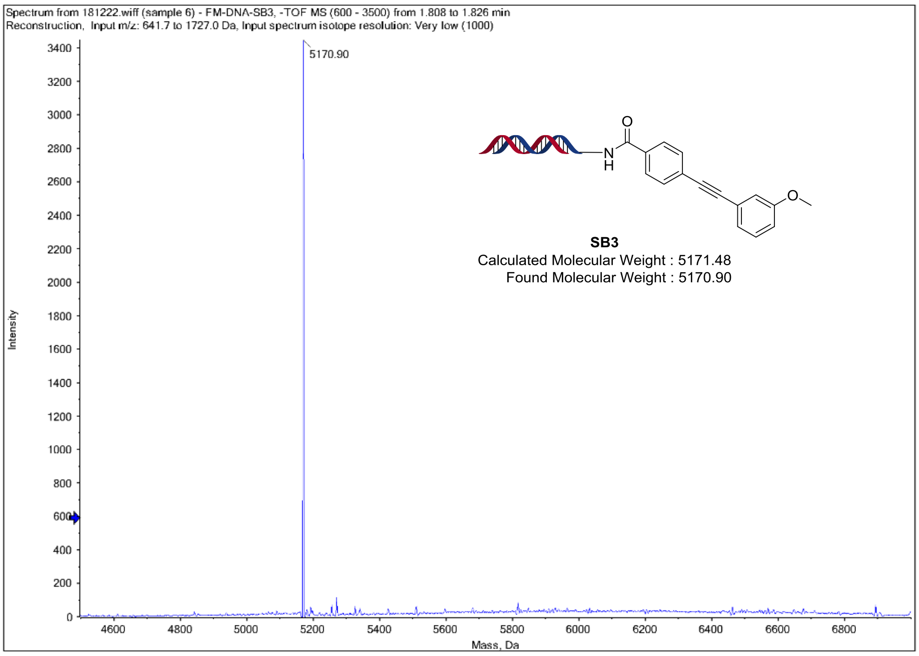
Spectrum from 181222.wiff (sample 6) - FM-DNA-SB3, -TOF MS (600 - 3500) from 1.808 to 1.826 min
Reconstruction, Input m/z: 641.7 to 1727.0 Da, Input spectrum isotope resolution: Very low (1000)
5170.90
SB3
Calculated Molecular Weight : 5171.48
Found Molecular Weight : 5170.90
Intensity
Mass, Da

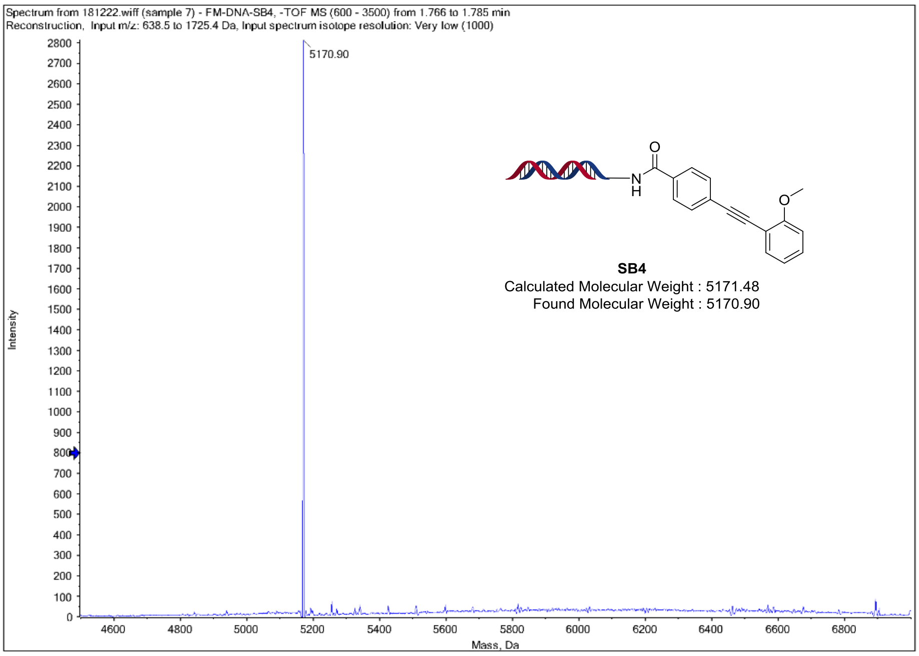
Spectrum from 181222.wiff (sample 7) - FM-DNA-SB4, -TOF MS (600 - 3500) from 1.766 to 1.785 min
Reconstruction, Input m/z: 638.5 to 1725.4 Da, Input spectrum isotope resolution: Very low (1000)
5170.90
SB4
Calculated Molecular Weight : 5171.48
Found Molecular Weight : 5170.90
Intensity
Mass, Da

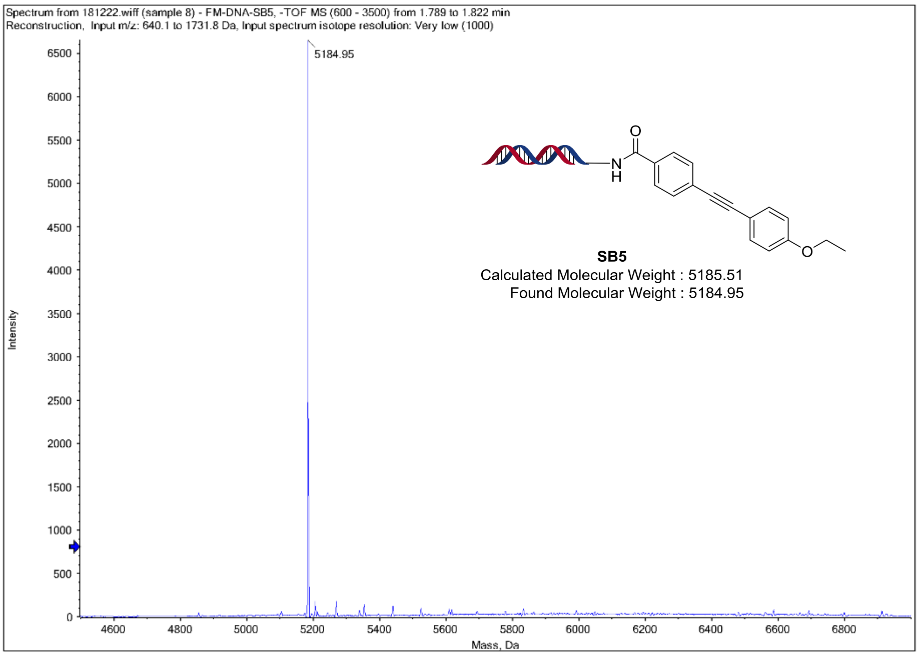
Spectrum from 181222.wiff (sample 8) - FM-DNA-SB5, -TOF MS (600 - 3500) from 1.789 to 1.822 min
Reconstruction, Input m/z: 640.1 to 1731.8 Da, Input spectrum isotope resolution: Very low (1000)
5184.95
Intensity
Mass, Da
SB5
Calculated Molecular Weight : 5185.51
Found Molecular Weight : 5184.95

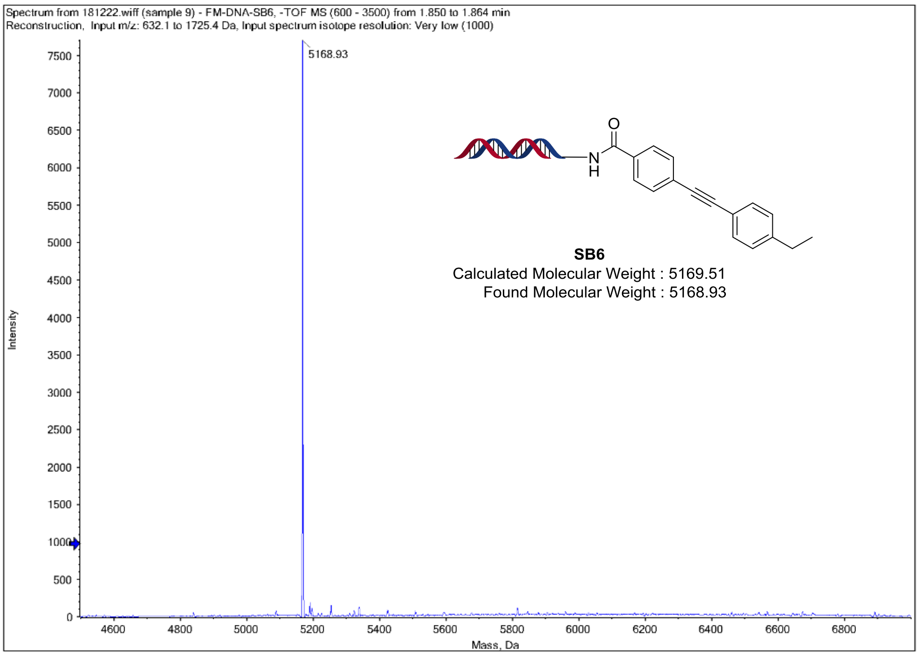
Spectrum from 181222.wiff (sample 9) - FM-DNA-SB6, -TOF MS (600 - 3500) from 1.850 to 1.864 min
Reconstruction, Input m/z: 632.1 to 1725.4 Da, Input spectrum isotope resolution: Very low (1000)
5168.93
Intensity
Mass, Da
SB6
Calculated Molecular Weight : 5169.51
Found Molecular Weight : 5168.93

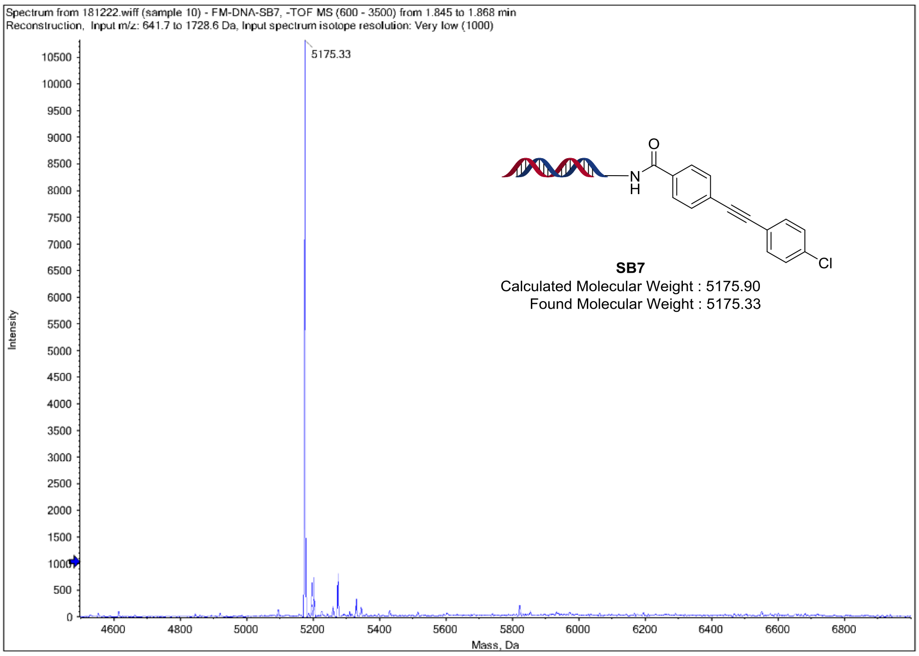

Spectrum from 181222.wiff (sample 10) - FM-DNA-SB7, -TOF MS (600 - 3500) from 1.845 to 1.868 min
Reconstruction, Input m/z: 641.7 to 1728.6 Da, Input spectrum isotope resolution: Very low (1000)
5175.33
Intensity
Mass, Da
O
N
H
Cl
SB7
Calculated Molecular Weight : 5175.90
Found Molecular Weight : 5175.33

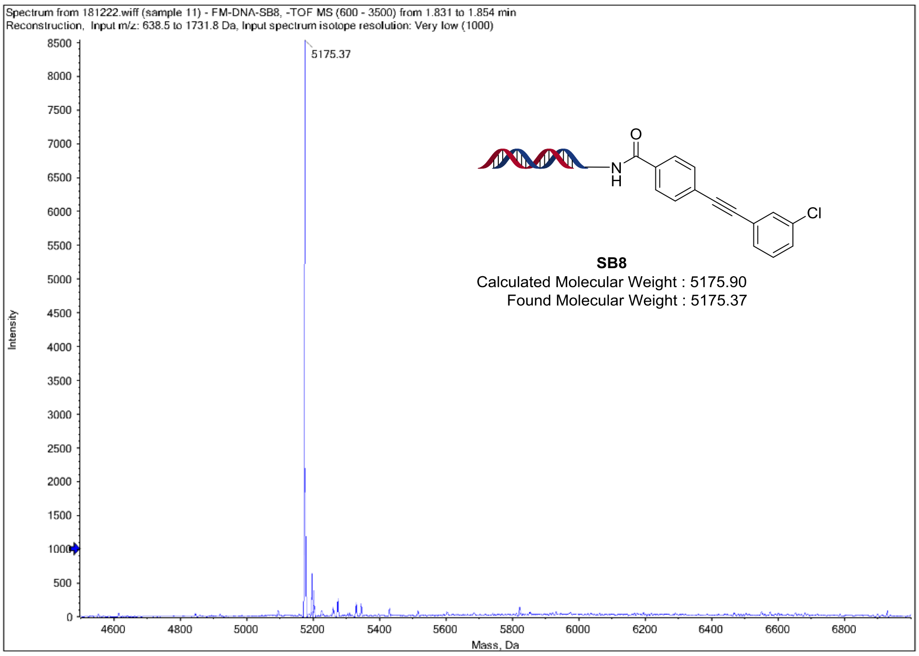

Spectrum from 181222.wiff (sample 11) - FM-DNA-SB8, -TOF MS (600 - 3500) from 1.831 to 1.854 min
Reconstruction, Input m/z: 638.5 to 1731.8 Da, Input spectrum isotope resolution: Very low (1000)
5175.37
Intensity
Mass, Da
O
N
H
Cl
SB8
Calculated Molecular Weight : 5175.90
Found Molecular Weight : 5175.37

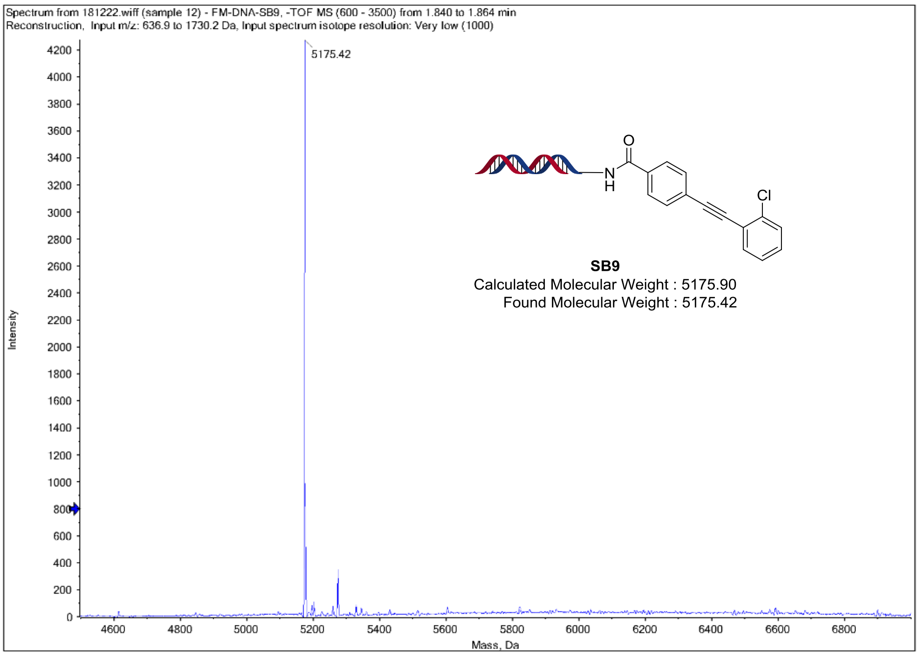
Spectrum from 181222.wiff (sample 12) - FM-DNA-SB9, -TOF MS (600 - 3500) from 1.840 to 1.864 min
Reconstruction, Input m/z: 636.9 to 1730.2 Da, Input spectrum isotope resolution: Very low (1000)
5175.42
Intensity
Mass, Da
Cl
SB9
Calculated Molecular Weight : 5175.90
Found Molecular Weight : 5175.42

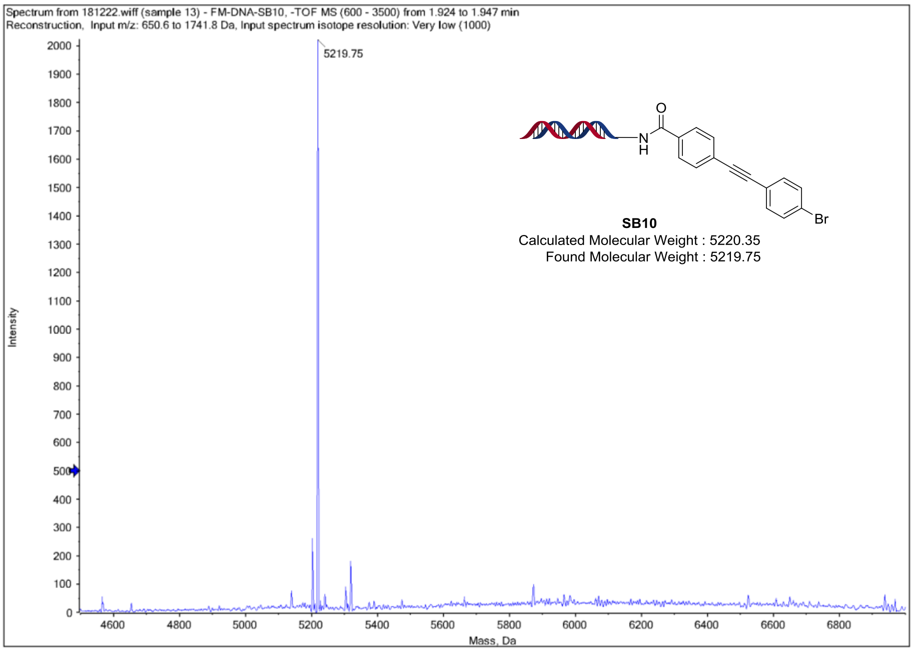
Spectrum from 181222.wiff (sample 13) - FM-DNA-SB10, -TOF MS (600 - 3500) from 1.924 to 1.947 min
Reconstruction, Input m/z: 650.6 to 1741.8 Da, Input spectrum isotope resolution: Very low (1000)
5219.75
Intensity
Mass, Da
Br
SB10
Calculated Molecular Weight : 5220.35
Found Molecular Weight : 5219.75

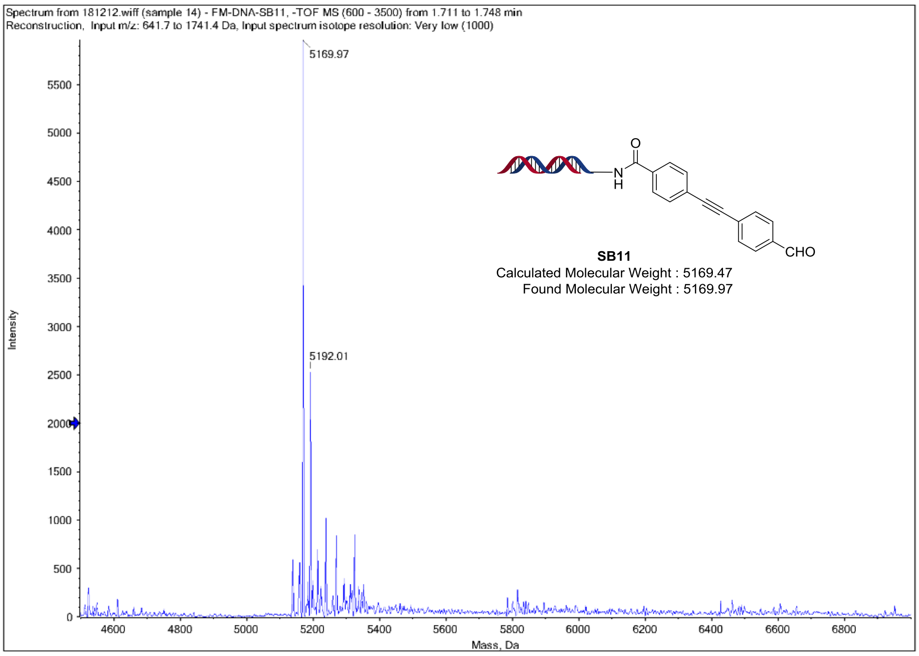
Spectrum from 181212.wiff (sample 14) - FM-DNA-SB11, -TOF MS (600 - 3500) from 1.711 to 1.748 min
Reconstruction, Input m/z: 641.7 to 1741.4 Da, Input spectrum isotope resolution: Very low (1000)
5169.97
5192.01
Intensity
Mass, Da
CHO
SB11
Calculated Molecular Weight : 5169.47
Found Molecular Weight : 5169.97

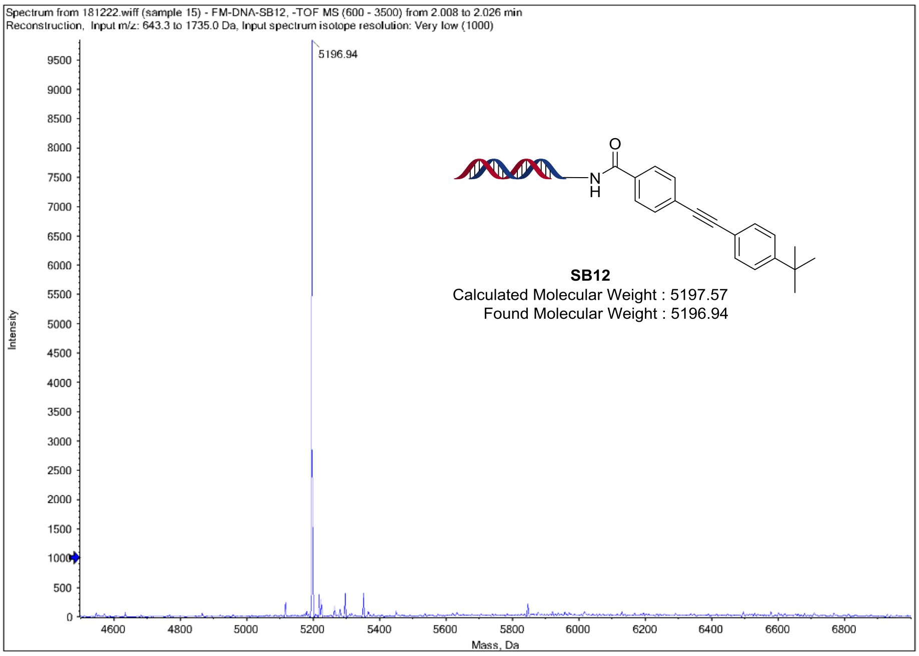
Spectrum from 181222.wiff (sample 15) - FM-DNA-SB12, -TOF MS (600 - 3500) from 2.008 to 2.026 min
Reconstruction, Input m/z: 643.3 to 1735.0 Da, Input spectrum isotope resolution: Very low (1000)
5196.94
Intensity
Mass, Da
SB12
Calculated Molecular Weight : 5197.57
Found Molecular Weight : 5196.94

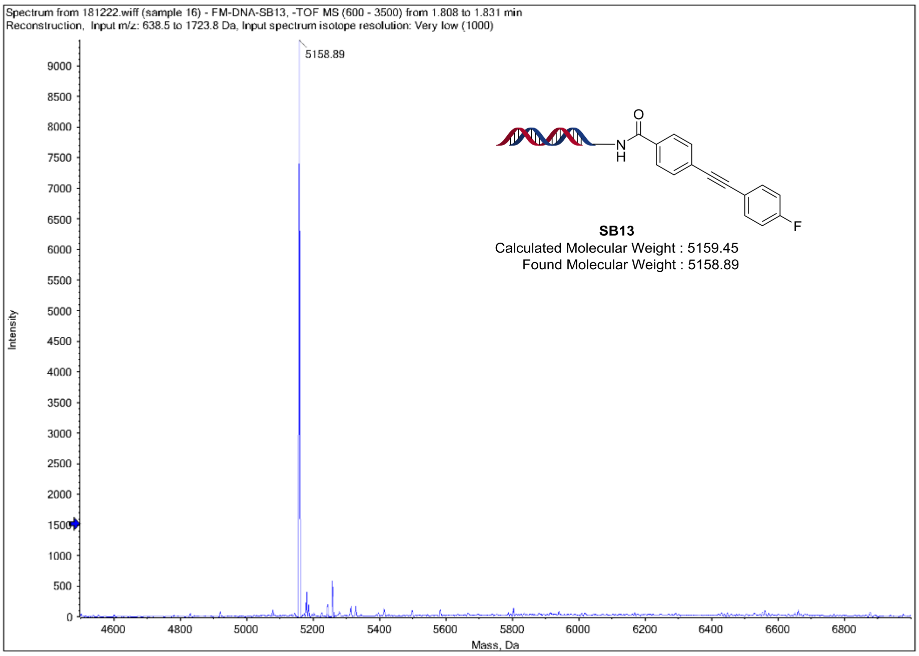
Spectrum from 181222.wiff (sample 16) - FM-DNA-SB13, -TOF MS (600 - 3500) from 1.808 to 1.831 min
Reconstruction, Input m/z: 638.5 to 1723.8 Da, Input spectrum isotope resolution: Very low (1000)
5158.89
SB13
Calculated Molecular Weight : 5159.45
Found Molecular Weight : 5158.89
Intensity
Mass, Da

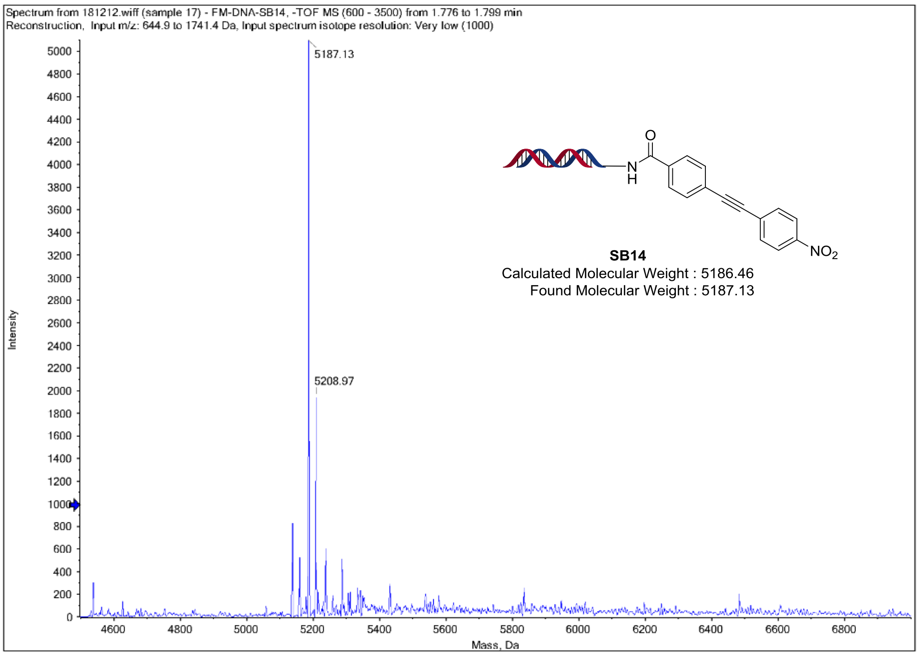
Spectrum from 181212.wiff (sample 17) - FM-DNA-SB14, -TOF MS (600 - 3500) from 1.776 to 1.799 min
Reconstruction, Input m/z: 644.9 to 1741.4 Da, Input spectrum isotope resolution: Very low (1000)
5187.13
5208.97
SB14
Calculated Molecular Weight : 5186.46
Found Molecular Weight : 5187.13
Intensity
Mass, Da

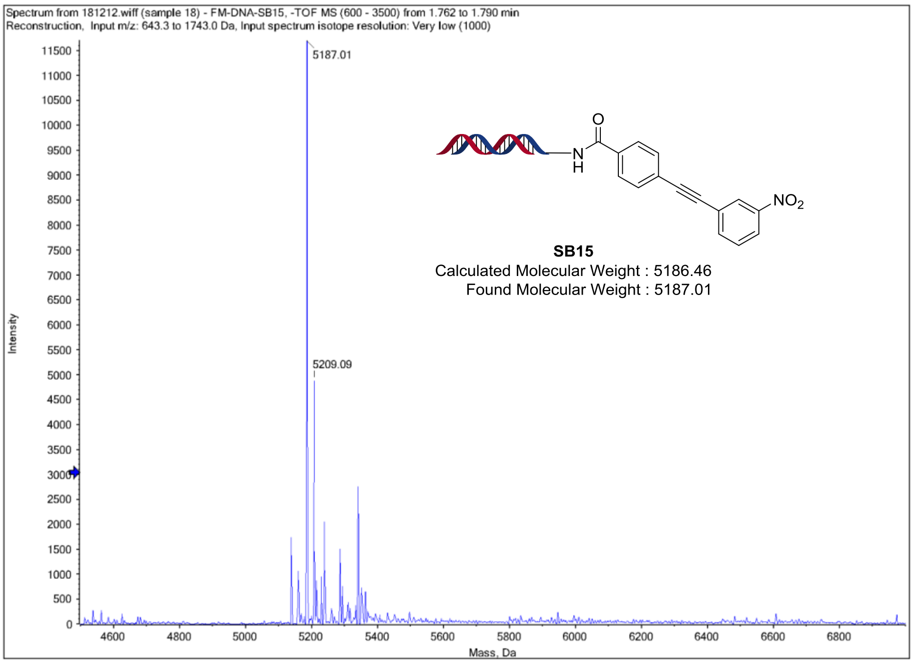
Spectrum from 181212.wiff (sample 18) - FM-DNA-SB15, -TOF MS (600 - 3500) from 1.762 to 1.790 min
Reconstruction, Input m/z: 643.3 to 1743.0 Da, Input spectrum isotope resolution: Very low (1000)
5187.01
5209.09
Intensity
Mass, Da
NO2
SB15
Calculated Molecular Weight : 5186.46
Found Molecular Weight : 5187.01

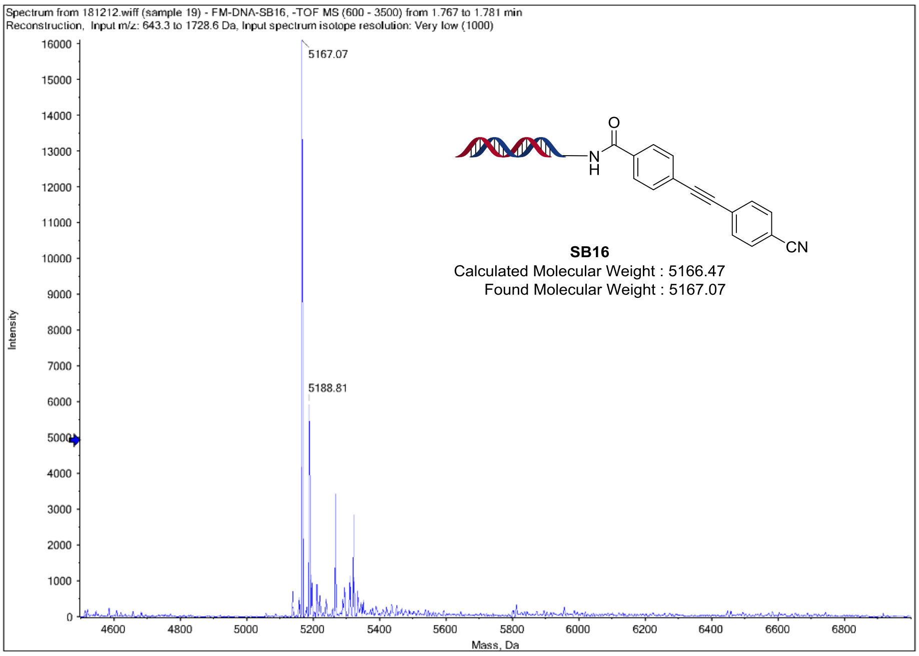
Spectrum from 181212.wiff (sample 19) - FM-DNA-SB16, -TOF MS (600 - 3500) from 1.767 to 1.781 min
Reconstruction, Input m/z: 643.3 to 1728.6 Da, Input spectrum isotope resolution: Very low (1000)
5167.07
5188.81
Intensity
Mass, Da
CN
SB16
Calculated Molecular Weight : 5166.47
Found Molecular Weight : 5167.07

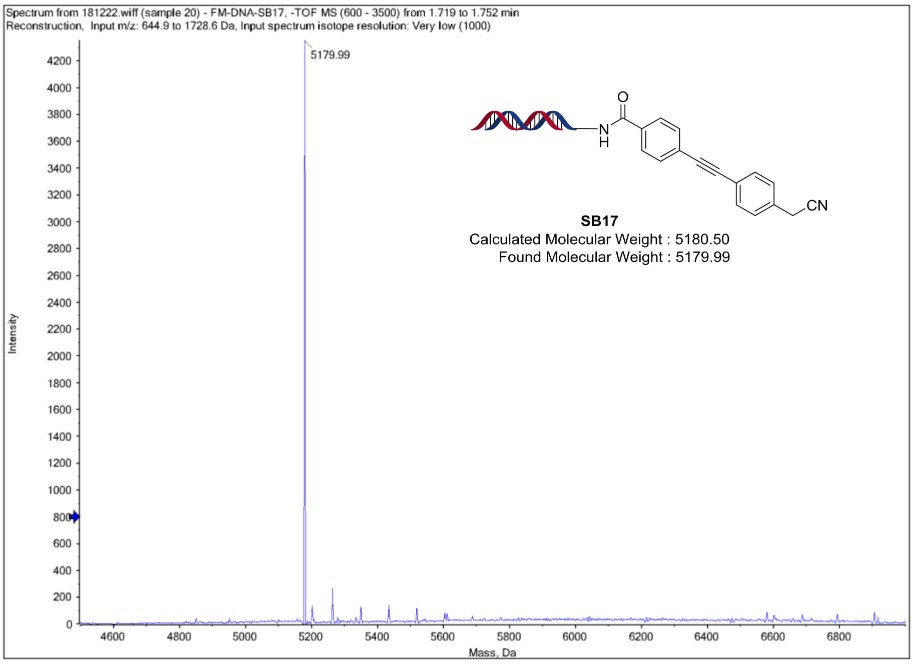
Spectrum from 181222.wiff (sample 20) - FM-DNA-SB17, -TOF MS (600 - 3500) from 1.719 to 1.752 min
Reconstruction, Input m/z: 644.9 to 1728.6 Da, Input spectrum isotope resolution: Very low (1000)
5179.99
SB17
Calculated Molecular Weight : 5180.50
Found Molecular Weight : 5179.99
Intensity
Mass, Da

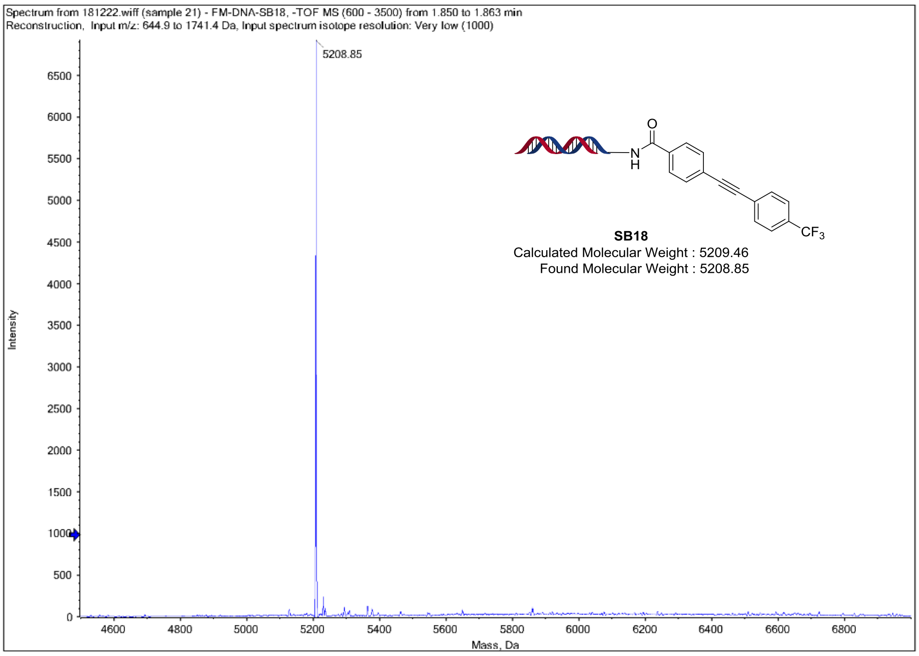
Spectrum from 181222.wiff (sample 21) - FM-DNA-SB18, -TOF MS (600 - 3500) from 1.850 to 1.863 min
Reconstruction, Input m/z: 644.9 to 1741.4 Da, Input spectrum isotope resolution: Very low (1000)
5208.85
SB18
Calculated Molecular Weight : 5209.46
Found Molecular Weight : 5208.85
Intensity
Mass, Da

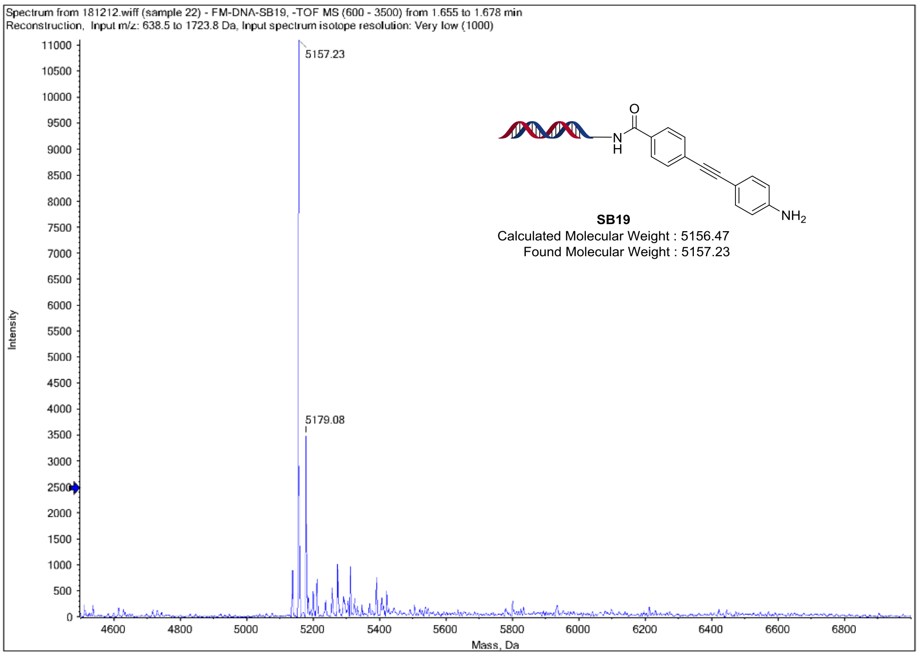
Spectrum from 181212.wiff (sample 22) - FM-DNA-SB19, -TOF MS (600 - 3500) from 1.655 to 1.678 min
Reconstruction, Input m/z: 638.5 to 1723.8 Da, Input spectrum isotope resolution: Very low (1000)
5157.23
5179.08
Intensity
Mass, Da
SB19
Calculated Molecular Weight : 5156.47
Found Molecular Weight : 5157.23

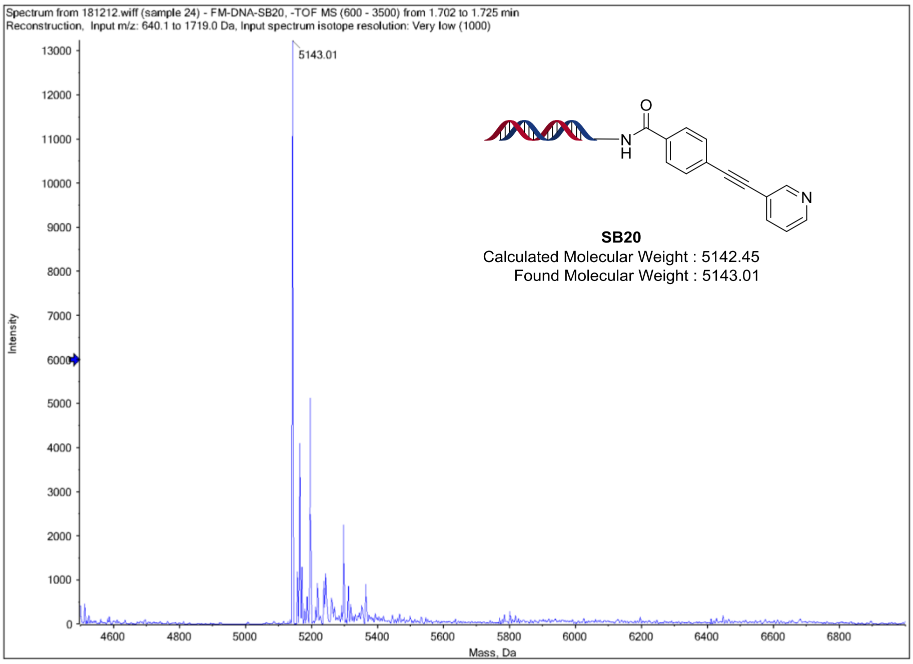
Spectrum from 181212.wiff (sample 24) - FM-DNA-SB20, -TOF MS (600 - 3500) from 1.702 to 1.725 min
Reconstruction, Input m/z: 640.1 to 1719.0 Da, Input spectrum isotope resolution: Very low (1000)
5143.01
Intensity
Mass, Da
SB20
Calculated Molecular Weight : 5142.45
Found Molecular Weight : 5143.01

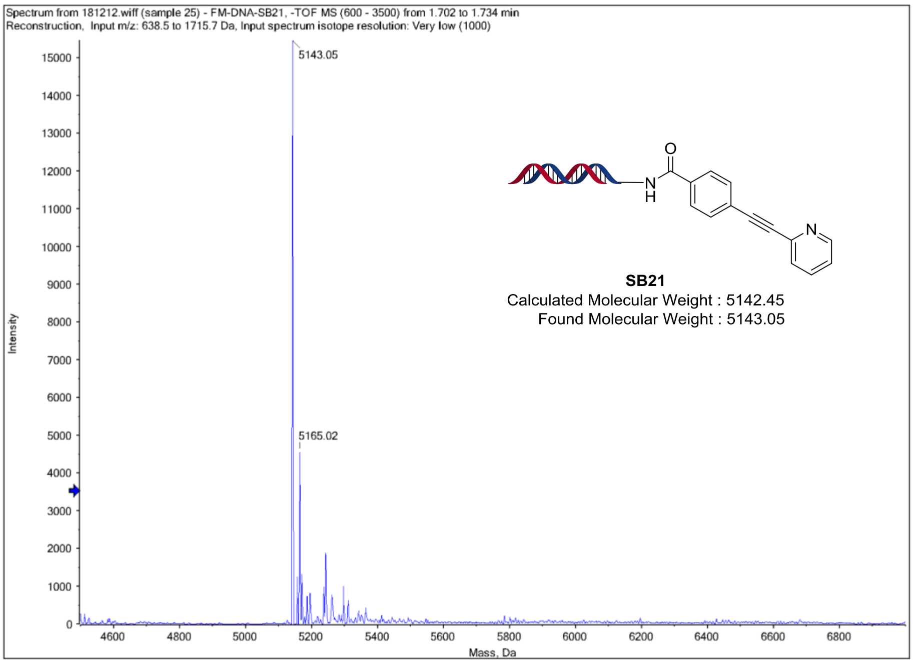
Spectrum from 181212.wiff (sample 25) - FM-DNA-SB21, -TOF MS (600 - 3500) from 1.702 to 1.734 min
Reconstruction, Input m/z: 638.5 to 1715.7 Da, Input spectrum isotope resolution: Very low (1000)
5143.05
5165.02
Intensity
Mass, Da
SB21
Calculated Molecular Weight : 5142.45
Found Molecular Weight : 5143.05

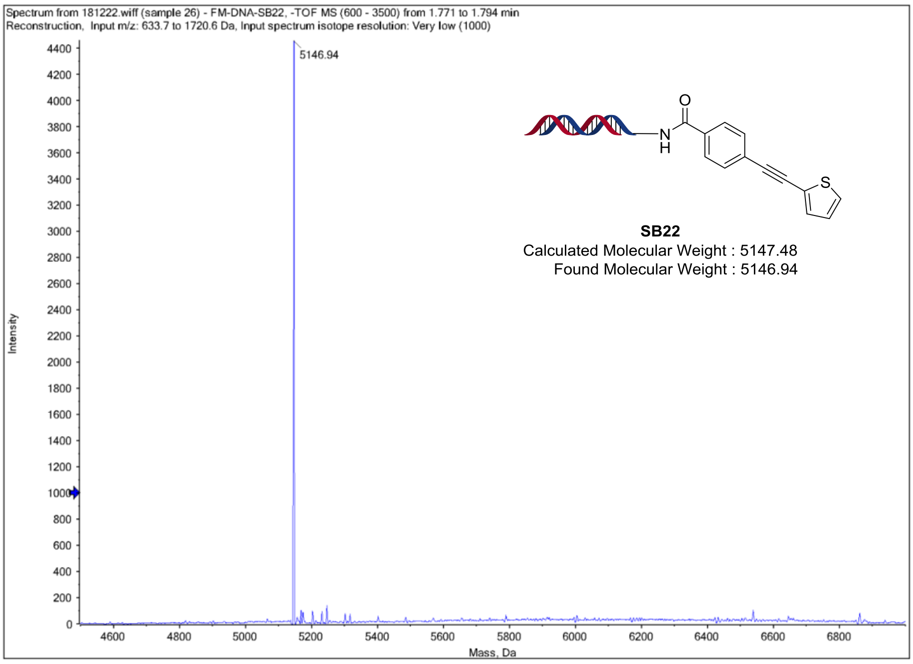
Spectrum from 181222.wiff (sample 26) - FM-DNA-SB22, -TOF MS (600 - 3500) from 1.771 to 1.794 min
Reconstruction, Input m/z: 633.7 to 1720.6 Da, Input spectrum isotope resolution: Very low (1000)
5146.94
Intensity
Mass, Da
SB22
Calculated Molecular Weight : 5147.48
Found Molecular Weight : 5146.94

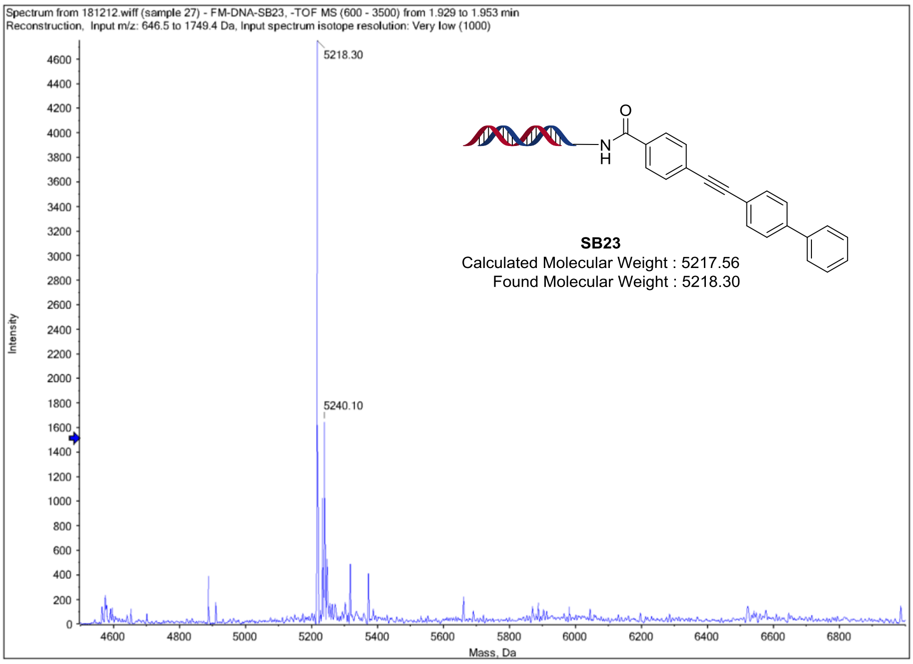
Spectrum from 181212.wiff (sample 27) - FM-DNA-SB23, -TOF MS (600 - 3500) from 1.929 to 1.953 min
Reconstruction, Input m/z: 646.5 to 1749.4 Da, Input spectrum isotope resolution: Very low (1000)
5218.30
5240.10
Intensity
Mass, Da
SB23
Calculated Molecular Weight : 5217.56
Found Molecular Weight : 5218.30

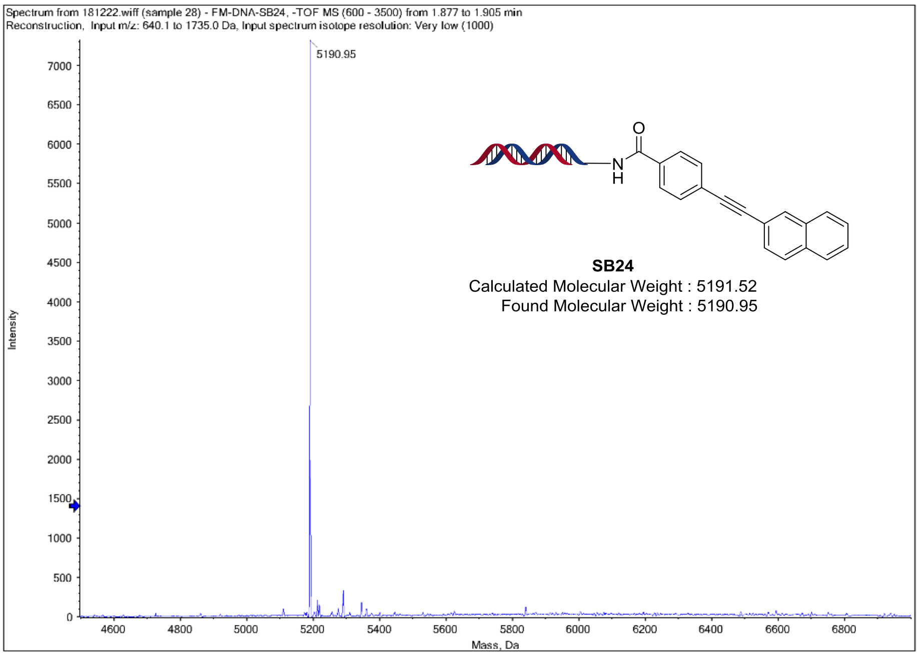
Spectrum from 181222.wiff (sample 28) - FM-DNA-SB24, -TOF MS (600 - 3500) from 1.877 to 1.905 min
Reconstruction, Input m/z: 640.1 to 1735.0 Da, Input spectrum isotope resolution: Very low (1000)
5190.95
Intensity
Mass, Da
SB24
Calculated Molecular Weight : 5191.52
Found Molecular Weight : 5190.95

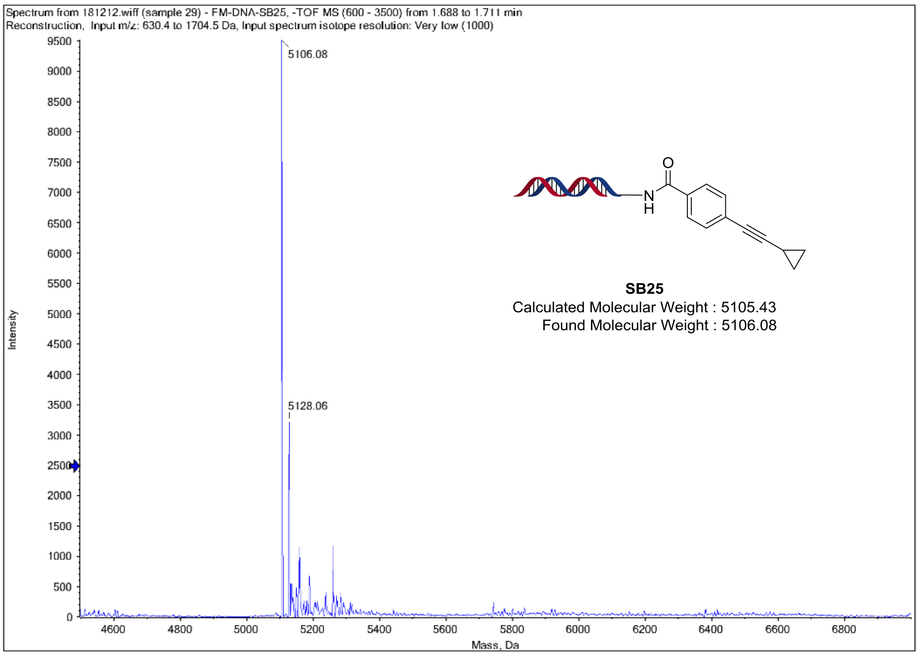
Spectrum from 181212.wiff (sample 29) - FM-DNA-SB25, -TOF MS (600 - 3500) from 1.688 to 1.711 min
Reconstruction, Input m/z: 630.4 to 1704.5 Da, Input spectrum isotope resolution: Very low (1000)
5106.08
5128.06
Intensity
Mass, Da
SB25
Calculated Molecular Weight : 5105.43
Found Molecular Weight : 5106.08

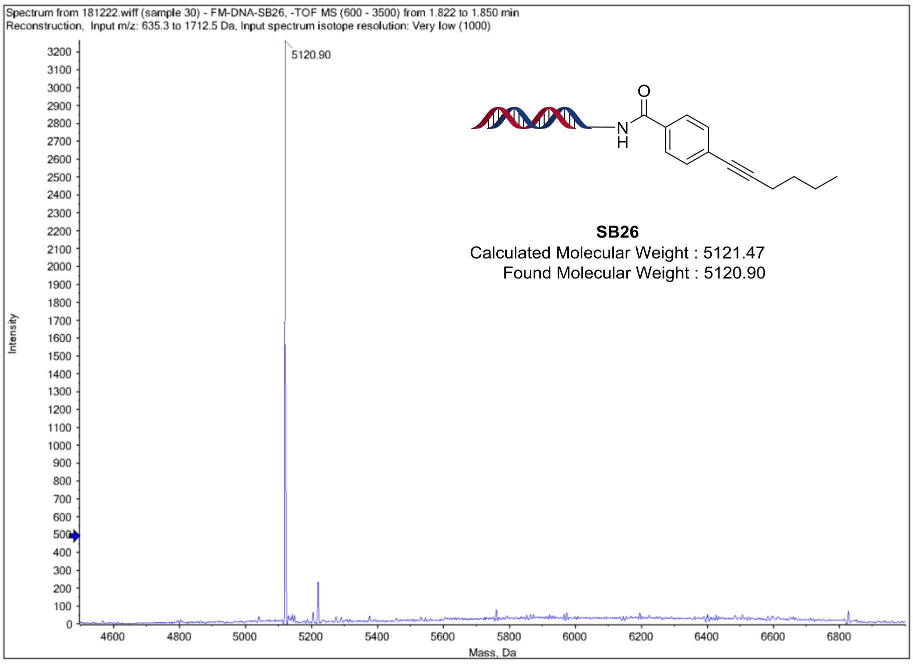
Spectrum from 181222.wiff (sample 30) - FM-DNA-SB26, -TOF MS (600 - 3500) from 1.822 to 1.850 min
Reconstruction, Input m/z: 635.3 to 1712.5 Da, Input spectrum isotope resolution: Very low (1000)
5120.90
Intensity
Mass, Da
SB26
Calculated Molecular Weight : 5121.47
Found Molecular Weight : 5120.90

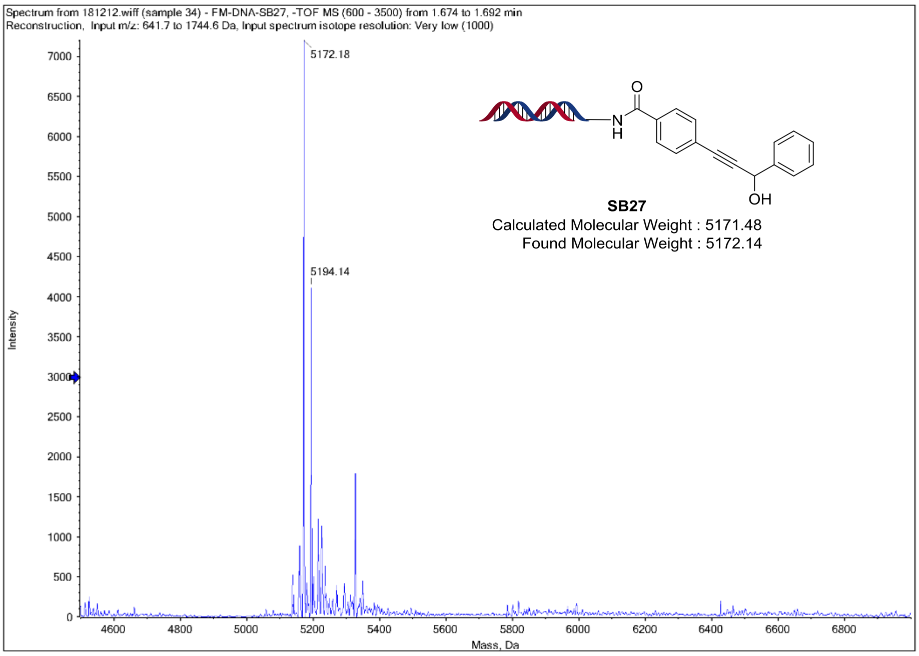

Spectrum from 181212.wiff (sample 34) - FM-DNA-SB27, -TOF MS (600 - 3500) from 1.674 to 1.692 min
Reconstruction, Input m/z: 641.7 to 1744.6 Da, Input spectrum isotope resolution: Very low (1000)
5172.18
5194.14
Intensity
Mass, Da
O
N
H
OH
SB27
Calculated Molecular Weight : 5171.48
Found Molecular Weight : 5172.14

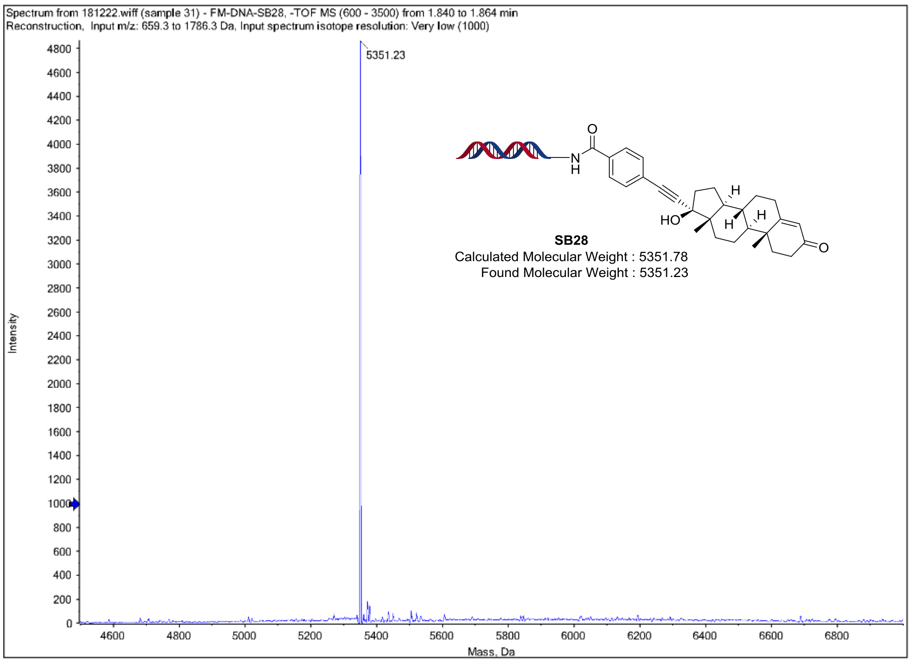

Spectrum from 181222.wiff (sample 31) - FM-DNA-SB28, -TOF MS (600 - 3500) from 1.840 to 1.864 min
Reconstruction, Input m/z: 659.3 to 1786.3 Da, Input spectrum isotope resolution: Very low (1000)
5351.23
Intensity
Mass, Da
O
N
H
HO
H
H
H
O
SB28
Calculated Molecular Weight : 5351.78
Found Molecular Weight : 5351.23

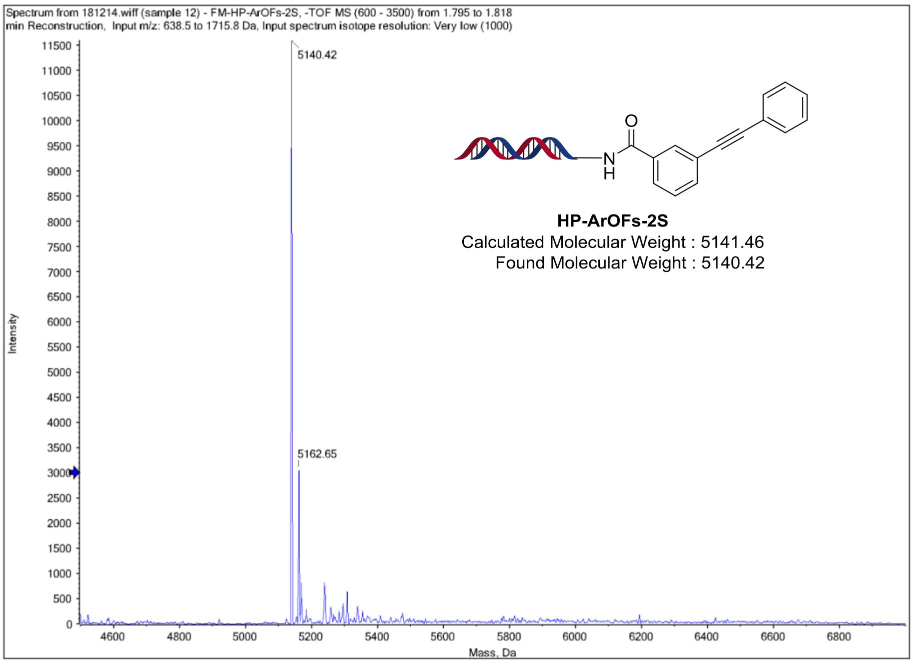

Spectrum from 181214.wiff (sample 12) - FM-HP-ArOFs-2S, -TOF MS (600 - 3500) from 1.795 to 1.818 min Reconstruction, Input m/z: 638.5 to 1715.8 Da, Input spectrum isotope resolution: Very low (1000)
5140.42
5162.65
Intensity
Mass, Da
O
N
H
HP-ArOFs-2S
Calculated Molecular Weight : 5141.46
Found Molecular Weight : 5140.42

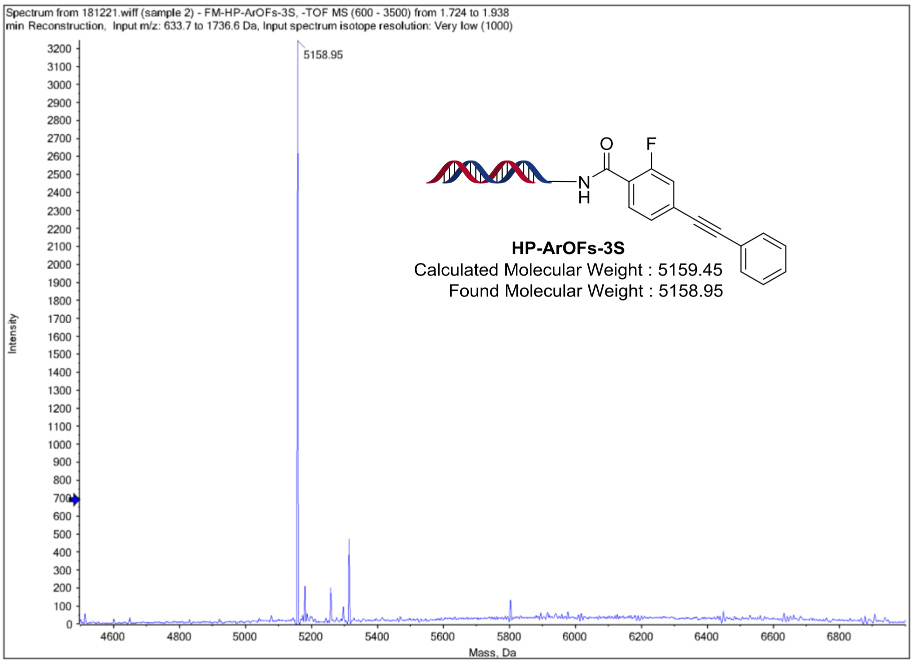

Spectrum from 181221.wiff (sample 2) - FM-HP-ArOFs-3S, -TOF MS (600 - 3500) from 1.724 to 1.938 min Reconstruction, Input m/z: 633.7 to 1736.6 Da, Input spectrum isotope resolution: Very low (1000)
5158.95
Intensity
Mass, Da
O
F
N
H
HP-ArOFs-3S
Calculated Molecular Weight : 5159.45
Found Molecular Weight : 5158.95

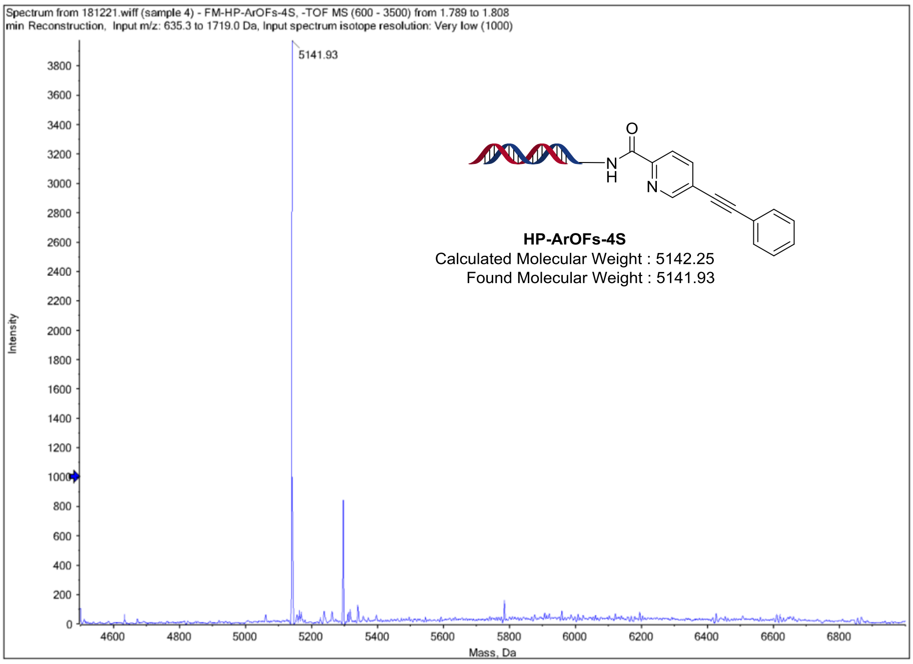
Spectrum from 181221.wiff (sample 4) - FM-HP-ArOFs-4S, -TOF MS (600 - 3500) from 1.789 to 1.808 min Reconstruction, Input m/z: 635.3 to 1719.0 Da, Input spectrum isotope resolution: Very low (1000)
5141.93
HP-ArOFs-4S
Calculated Molecular Weight : 5142.25
Found Molecular Weight : 5141.93
Intensity
Mass, Da

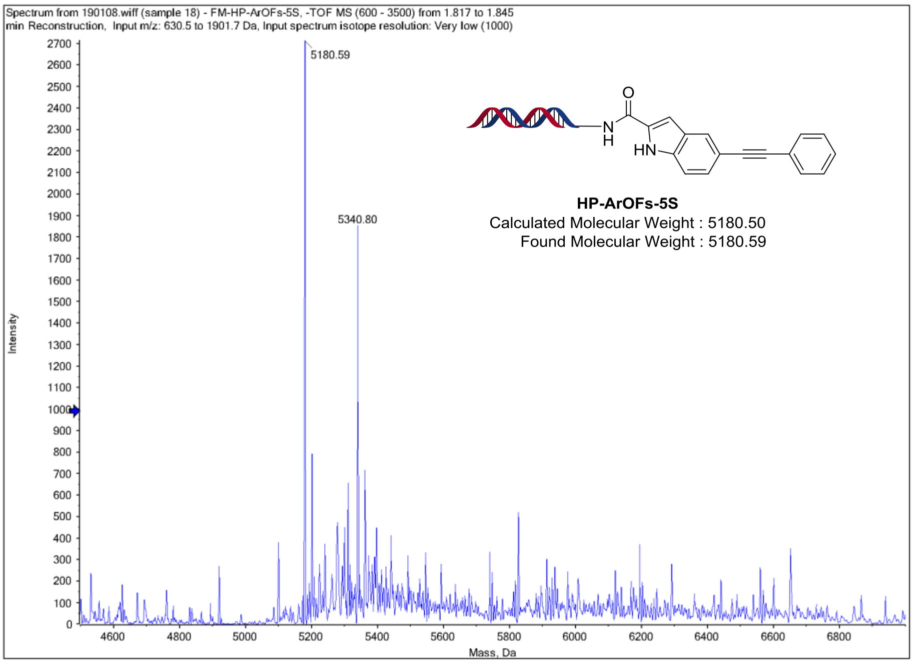
Spectrum from 190108.wiff (sample 18) - FM-HP-ArOFs-5S, -TOF MS (600 - 3500) from 1.817 to 1.845 min Reconstruction, Input m/z: 630.5 to 1901.7 Da, Input spectrum isotope resolution: Very low (1000)
5180.59
5340.80
HP-ArOFs-5S
Calculated Molecular Weight : 5180.50
Found Molecular Weight : 5180.59
Intensity
Mass, Da

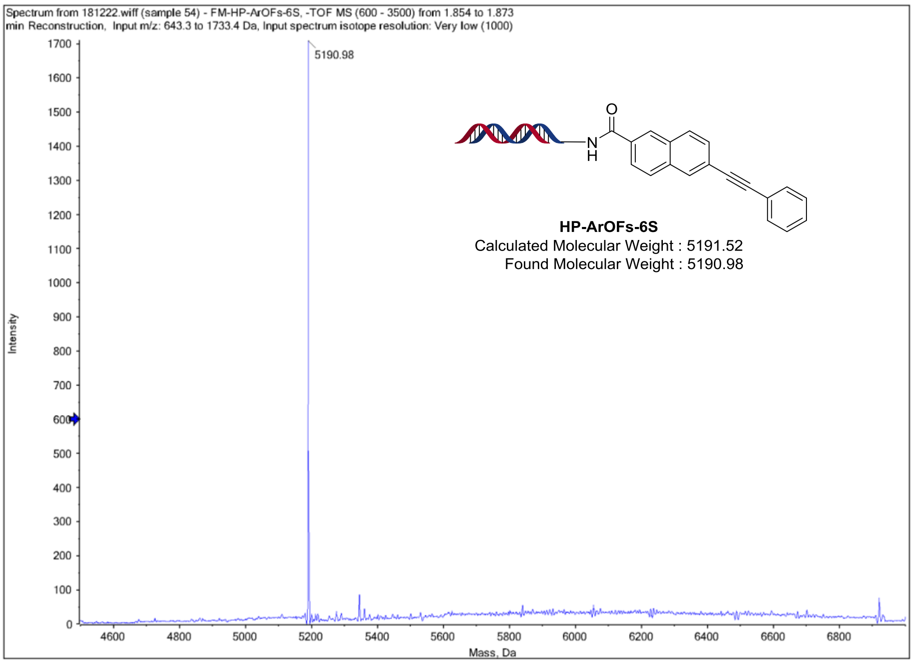
Spectrum from 181222.wiff (sample 54) - FM-HP-ArOFs-6S, -TOF MS (600 - 3500) from 1.854 to 1.873 min Reconstruction, Input m/z: 643.3 to 1733.4 Da, Input spectrum isotope resolution: Very low (1000)
5190.98
HP-ArOFs-6S
Calculated Molecular Weight : 5191.52
Found Molecular Weight : 5190.98
Intensity
Mass, Da

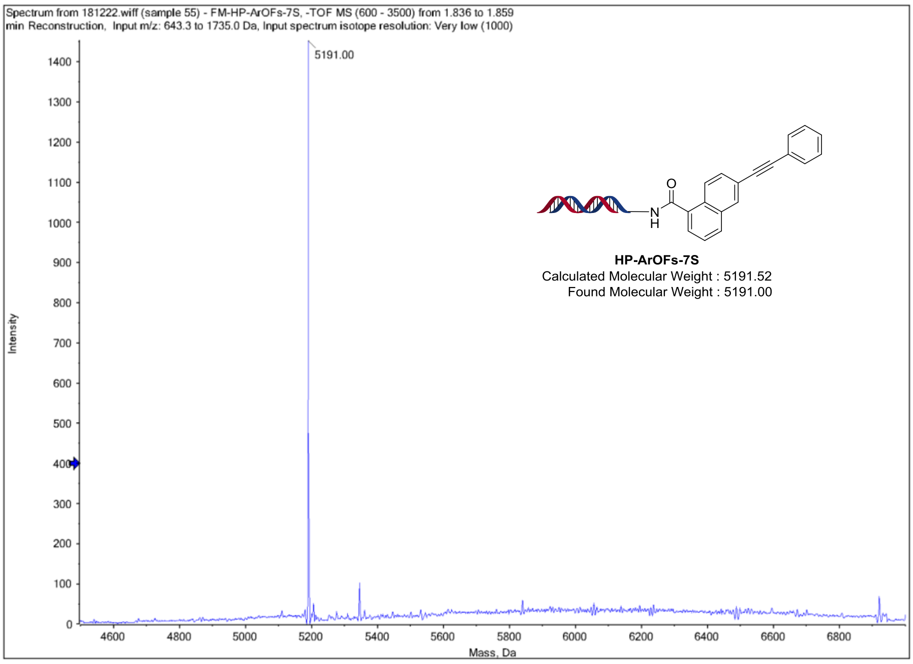
Spectrum from 181222.wiff (sample 55) - FM-HP-ArOFs-7S, -TOF MS (600 - 3500) from 1.836 to 1.859 min Reconstruction, Input m/z: 643.3 to 1735.0 Da, Input spectrum isotope resolution: Very low (1000)
5191.00
HP-ArOFs-7S
Calculated Molecular Weight : 5191.52
Found Molecular Weight : 5191.00
Intensity
Mass, Da

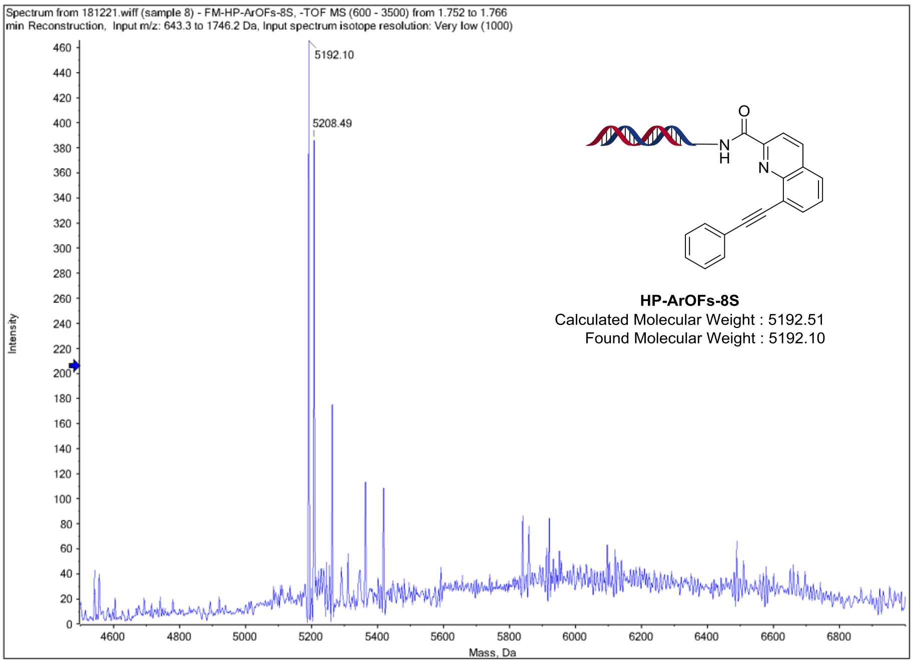
Spectrum from 181221.wiff (sample 8) - FM-HP-ArOFs-8S, -TOF MS (600 - 3500) from 1.752 to 1.766 min Reconstruction, Input m/z: 643.3 to 1746.2 Da, Input spectrum isotope resolution: Very low (1000)
5192.10
5208.49
Intensity
Mass, Da
HP-ArOFs-8S
Calculated Molecular Weight : 5192.51
Found Molecular Weight : 5192.10

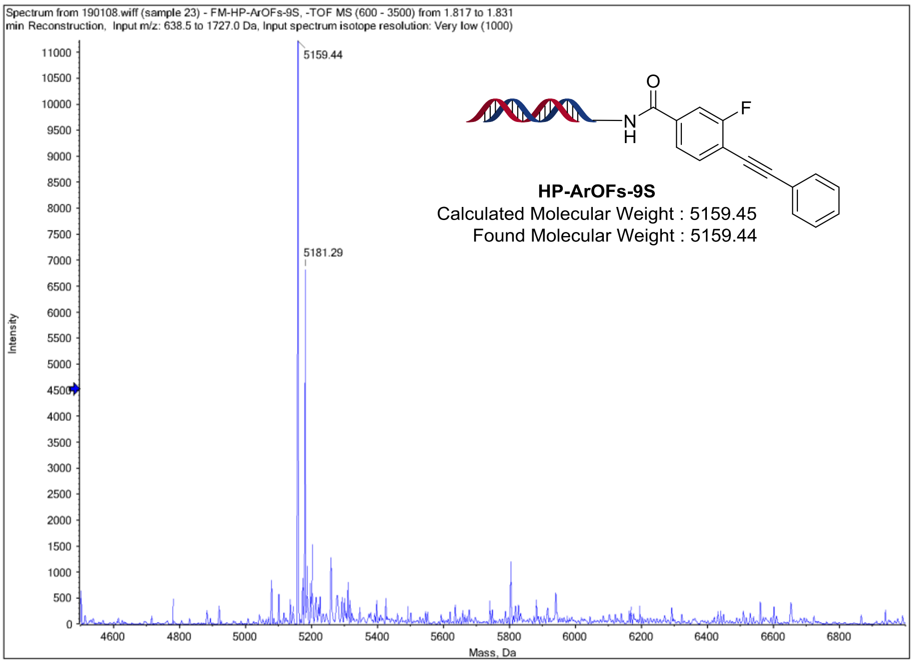
Spectrum from 190108.wiff (sample 23) - FM-HP-ArOFs-9S, -TOF MS (600 - 3500) from 1.817 to 1.831 min Reconstruction, Input m/z: 638.5 to 1727.0 Da, Input spectrum isotope resolution: Very low (1000)
5159.44
5181.29
Intensity
Mass, Da
HP-ArOFs-9S
Calculated Molecular Weight : 5159.45
Found Molecular Weight : 5159.44

MS Spectra of On-DNA Buchwald amination products

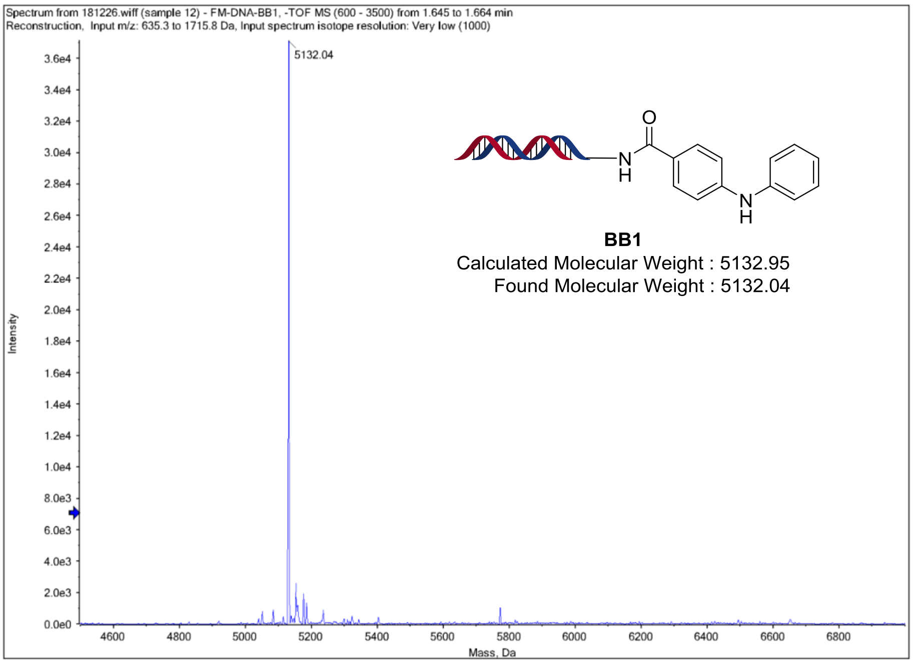

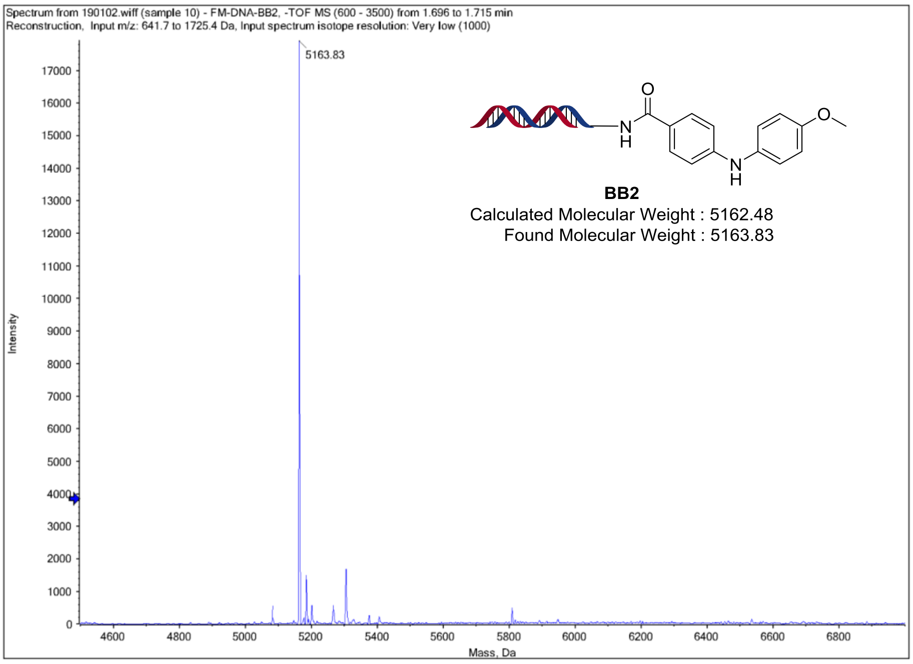
Spectrum from 190102.wiff (sample 10) - FM-DNA-BB2, -TOF MS (600 - 3500) from 1.696 to 1.715 min
Reconstruction, Input m/z: 641.7 to 1725.4 Da, Input spectrum isotope resolution: Very low (1000)
5163.83
BB2
Calculated Molecular Weight : 5162.48
Found Molecular Weight : 5163.83
Intensity
Mass, Da

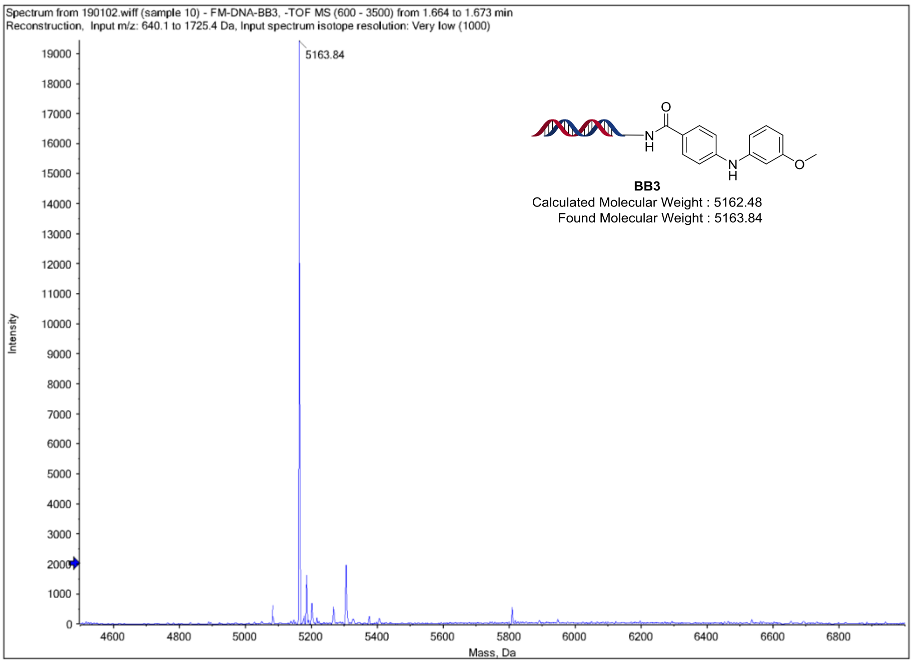
Spectrum from 190102.wiff (sample 10) - FM-DNA-BB3, -TOF MS (600 - 3500) from 1.664 to 1.673 min
Reconstruction, Input m/z: 640.1 to 1725.4 Da, Input spectrum isotope resolution: Very low (1000)
5163.84
BB3
Calculated Molecular Weight : 5162.48
Found Molecular Weight : 5163.84
Intensity
Mass, Da

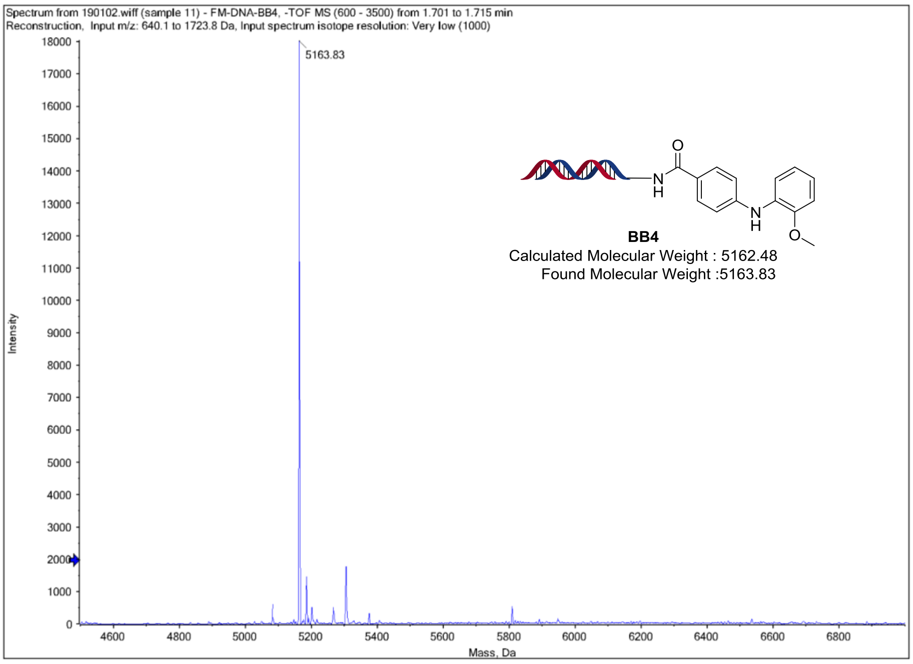
Spectrum from 190102.wiff (sample 11) - FM-DNA-BB4, -TOF MS (600 - 3500) from 1.701 to 1.715 min
Reconstruction, Input m/z: 640.1 to 1723.8 Da, Input spectrum isotope resolution: Very low (1000)
5163.83
BB4
Calculated Molecular Weight : 5162.48
Found Molecular Weight :5163.83
Intensity
Mass, Da

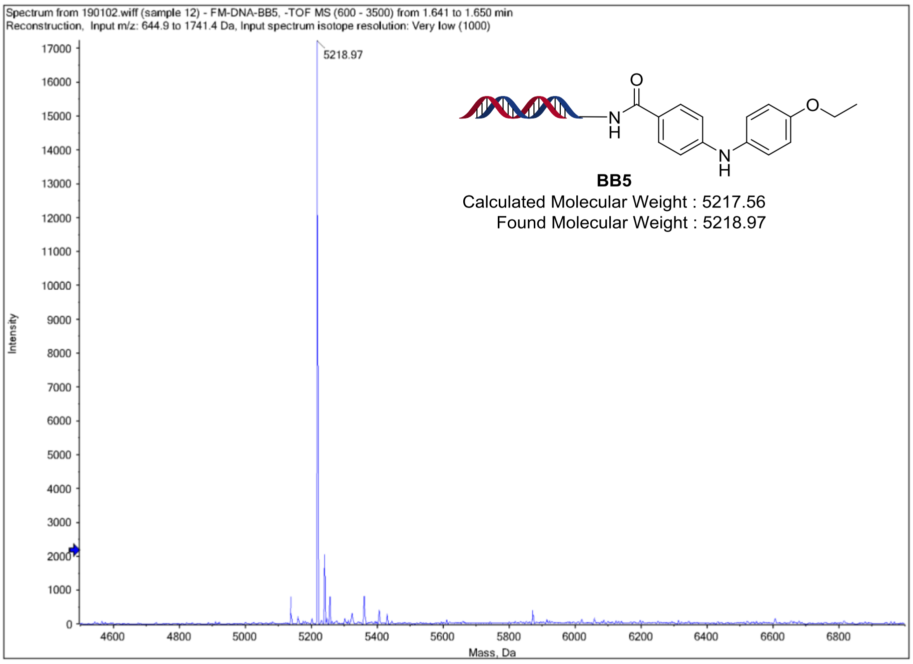
Spectrum from 190102.wiff (sample 12) - FM-DNA-BB5, -TOF MS (600 - 3500) from 1.641 to 1.650 min
Reconstruction, Input m/z: 644.9 to 1741.4 Da, Input spectrum isotope resolution: Very low (1000)
5218.97
BB5
Calculated Molecular Weight : 5217.56
Found Molecular Weight : 5218.97
Intensity
Mass, Da

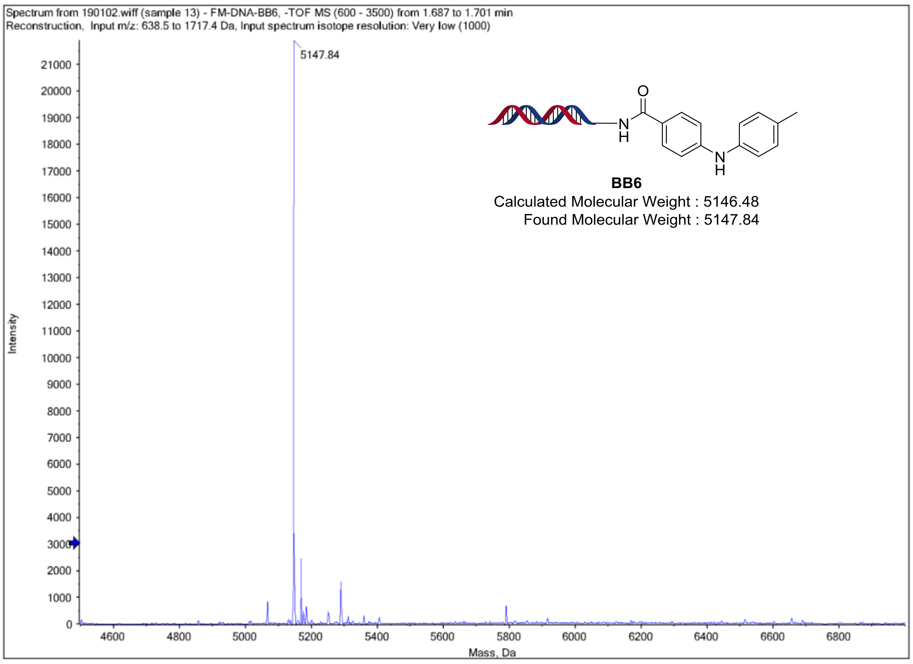
Spectrum from 190102.wiff (sample 13) - FM-DNA-BB6, -TOF MS (600 - 3500) from 1.687 to 1.701 min
Reconstruction, Input m/z: 638.5 to 1717.4 Da, Input spectrum isotope resolution: Very low (1000)
5147.84
BB6
Calculated Molecular Weight : 5146.48
Found Molecular Weight : 5147.84
Intensity
Mass, Da

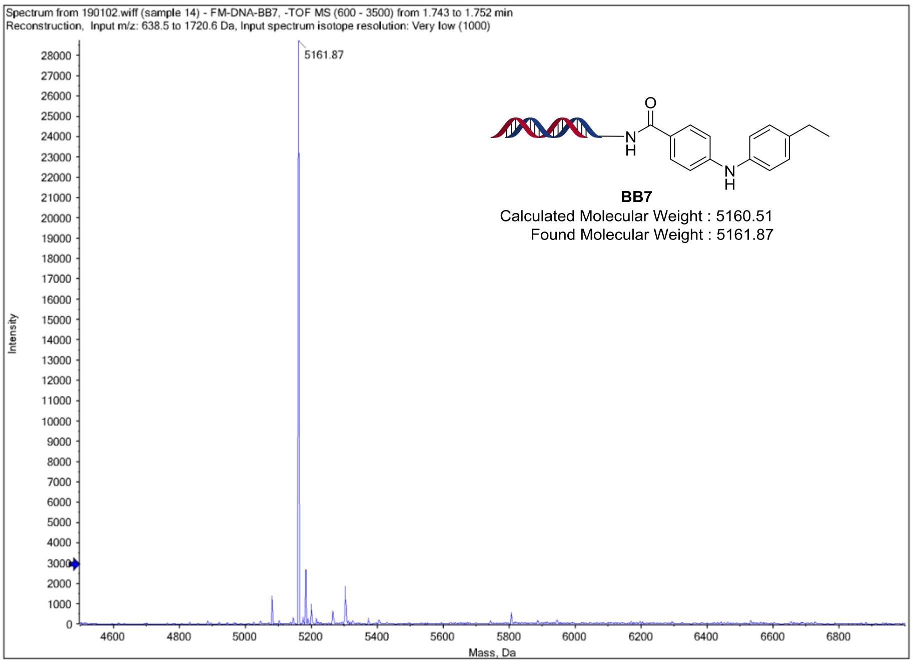
Spectrum from 190102.wiff (sample 14) - FM-DNA-BB7, -TOF MS (600 - 3500) from 1.743 to 1.752 min
Reconstruction, Input m/z: 638.5 to 1720.6 Da, Input spectrum isotope resolution: Very low (1000)
5161.87
BB7
Calculated Molecular Weight : 5160.51
Found Molecular Weight : 5161.87
Intensity
Mass, Da

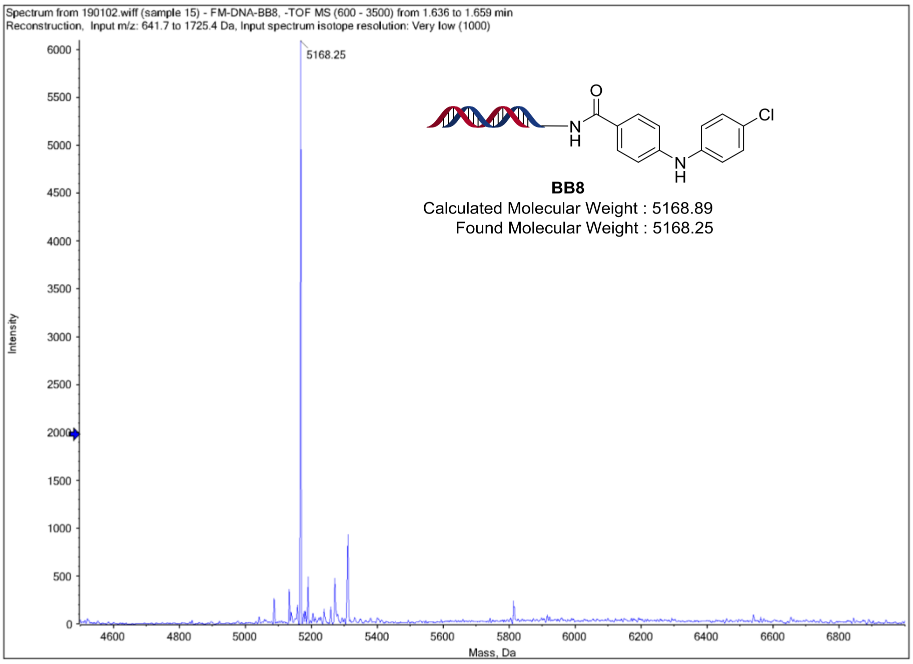

Spectrum from 190102.wiff (sample 15) - FM-DNA-BB8, -TOF MS (600 - 3500) from 1.636 to 1.659 min
Reconstruction, Input m/z: 641.7 to 1725.4 Da, Input spectrum isotope resolution: Very low (1000)
5168.25
BB8
Calculated Molecular Weight : 5168.89
Found Molecular Weight : 5168.25
Intensity
Mass, Da

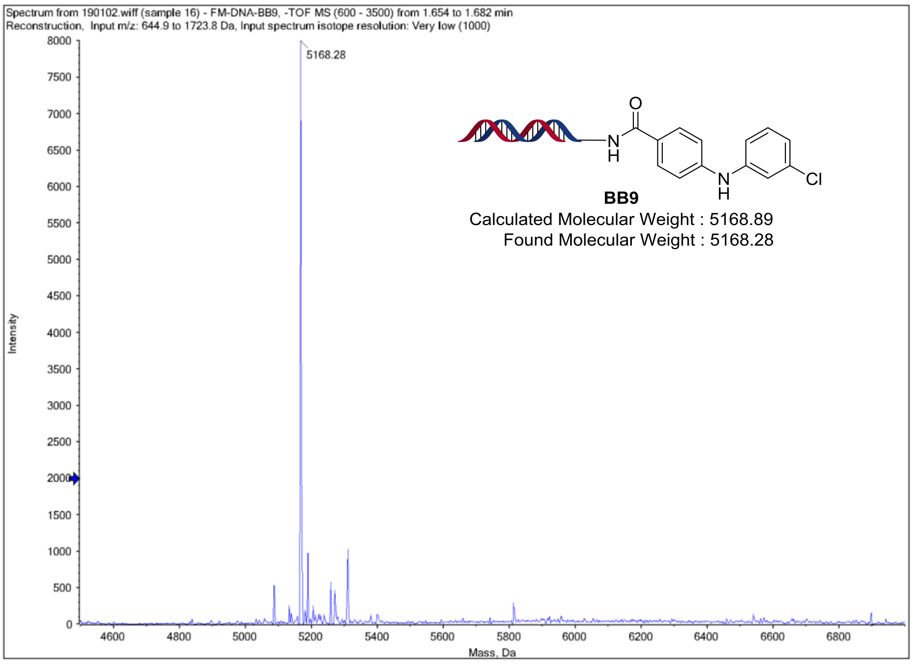

Spectrum from 190102.wiff (sample 16) - FM-DNA-BB9, -TOF MS (600 - 3500) from 1.654 to 1.682 min
Reconstruction, Input m/z: 644.9 to 1723.8 Da, Input spectrum isotope resolution: Very low (1000)
5168.28
BB9
Calculated Molecular Weight : 5168.89
Found Molecular Weight : 5168.28
Intensity
Mass, Da

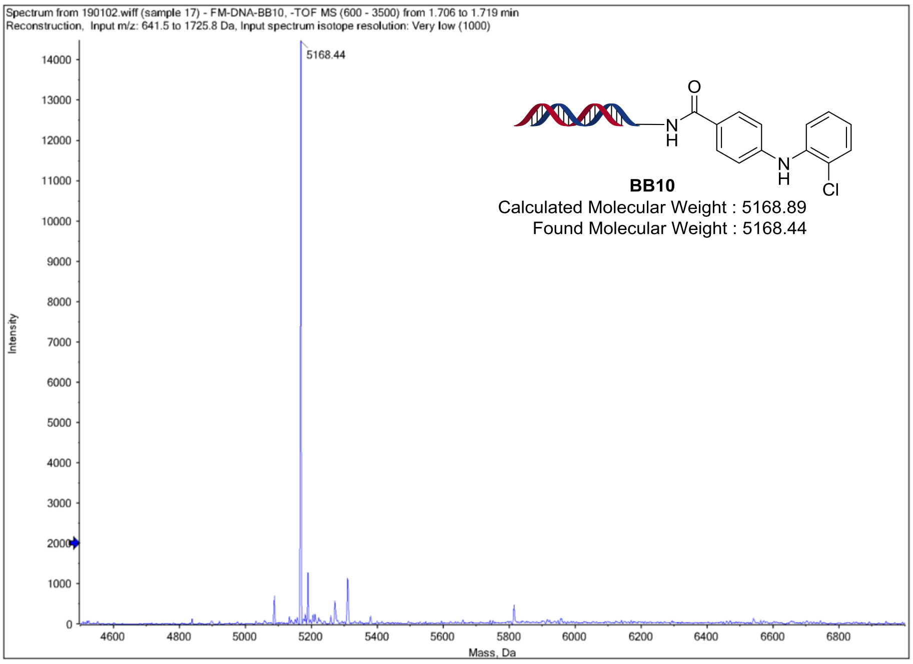
Spectrum from 190102.wiff (sample 17) - FM-DNA-BB10, -TOF MS (600 - 3500) from 1.706 to 1.719 min
Reconstruction, Input m/z: 641.5 to 1725.8 Da, Input spectrum isotope resolution: Very low (1000)
5168.44
Intensity
Mass, Da
BB10
Calculated Molecular Weight : 5168.89
Found Molecular Weight : 5168.44

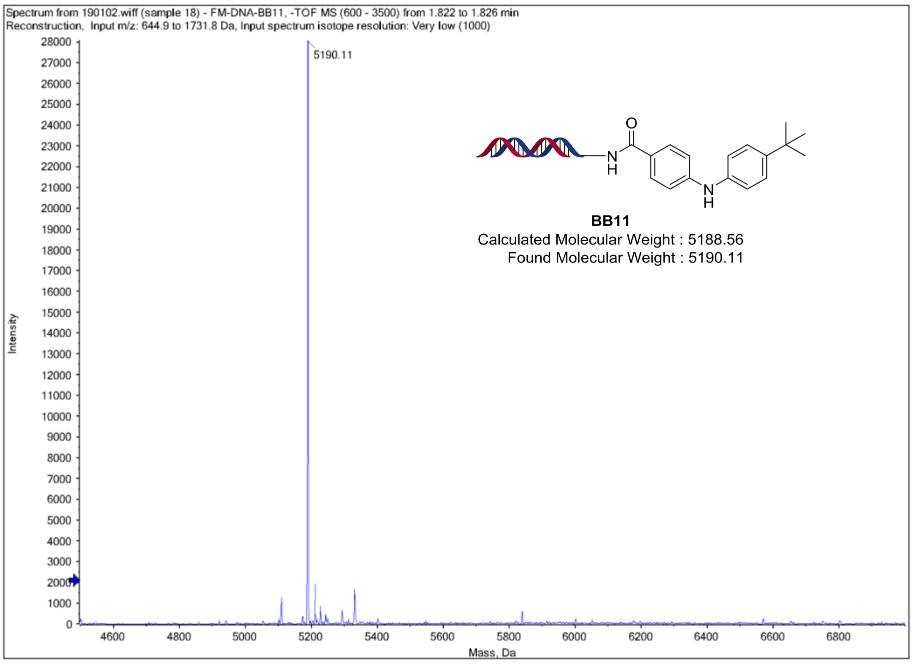
Spectrum from 190102.wiff (sample 18) - FM-DNA-BB11, -TOF MS (600 - 3500) from 1.822 to 1.826 min
Reconstruction, Input m/z: 644.9 to 1731.8 Da, Input spectrum isotope resolution: Very low (1000)
5190.11
Intensity
Mass, Da
BB11
Calculated Molecular Weight : 5188.56
Found Molecular Weight : 5190.11

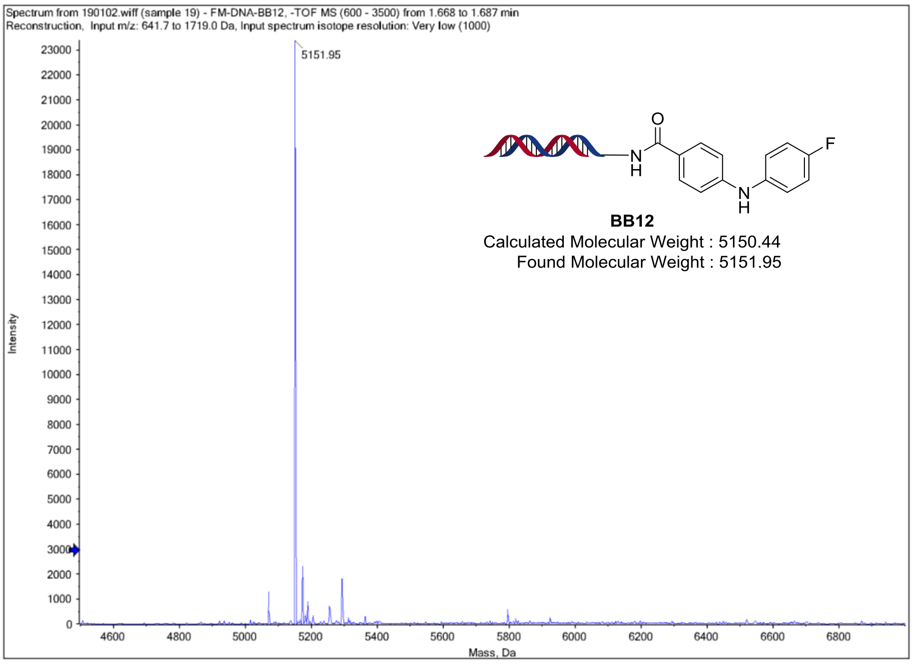
Spectrum from 190102.wiff (sample 19) - FM-DNA-BB12, -TOF MS (600 - 3500) from 1.668 to 1.687 min
Reconstruction, Input m/z: 641.7 to 1719.0 Da, Input spectrum isotope resolution: Very low (1000)
5151.95
BB12
Calculated Molecular Weight : 5150.44
Found Molecular Weight : 5151.95
Intensity
Mass, Da

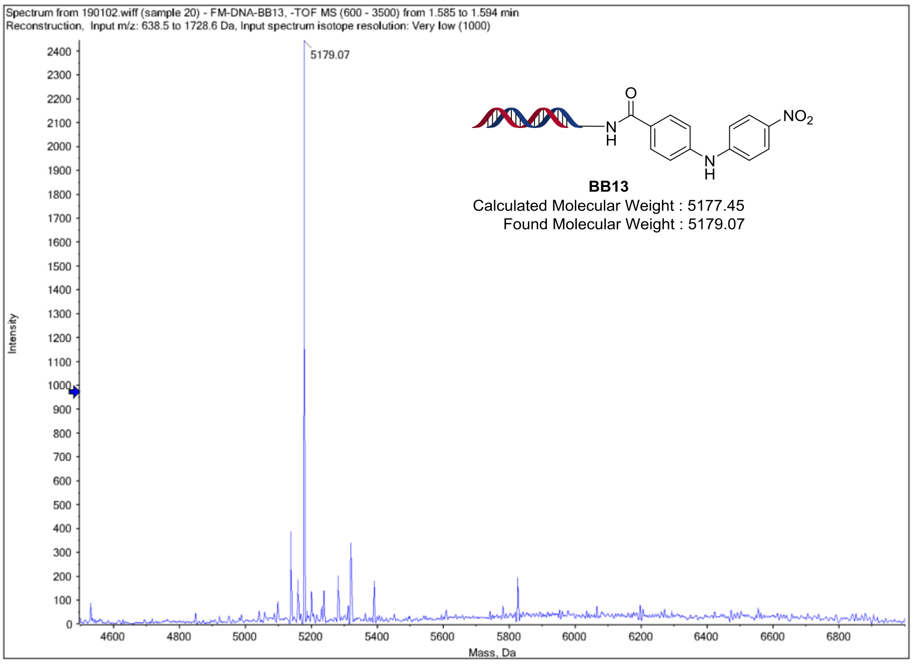
Spectrum from 190102.wiff (sample 20) - FM-DNA-BB13, -TOF MS (600 - 3500) from 1.585 to 1.594 min
Reconstruction, Input m/z: 638.5 to 1728.6 Da, Input spectrum isotope resolution: Very low (1000)
5179.07
BB13
Calculated Molecular Weight : 5177.45
Found Molecular Weight : 5179.07
Intensity
Mass, Da

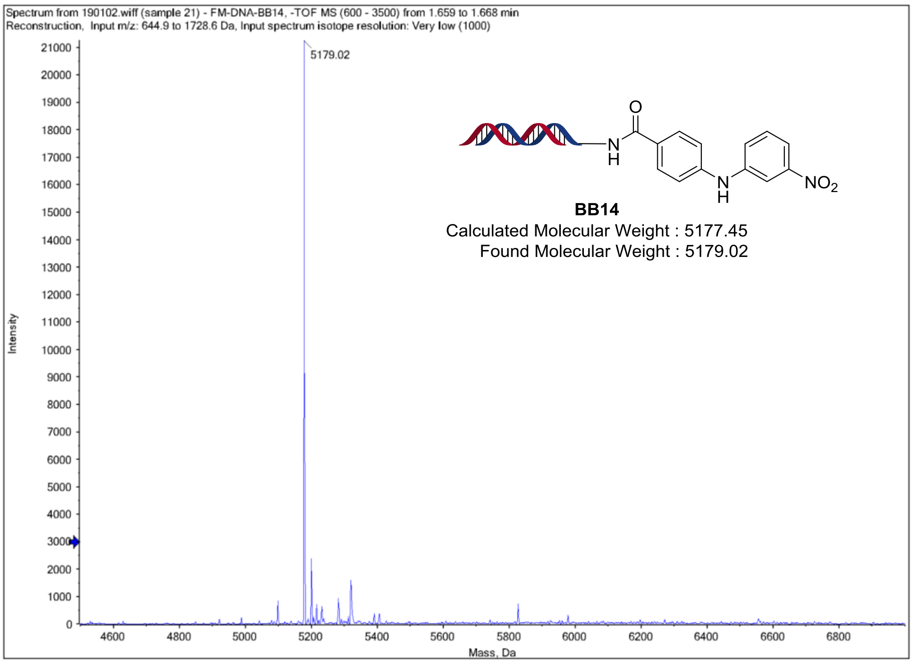
Spectrum from 190102.wiff (sample 21) - FM-DNA-BB14, -TOF MS (600 - 3500) from 1.659 to 1.668 min
Reconstruction, Input m/z: 644.9 to 1728.6 Da, Input spectrum isotope resolution: Very low (1000)
5179.02
Intensity
Mass, Da
NO2
BB14
Calculated Molecular Weight : 5177.45
Found Molecular Weight : 5179.02

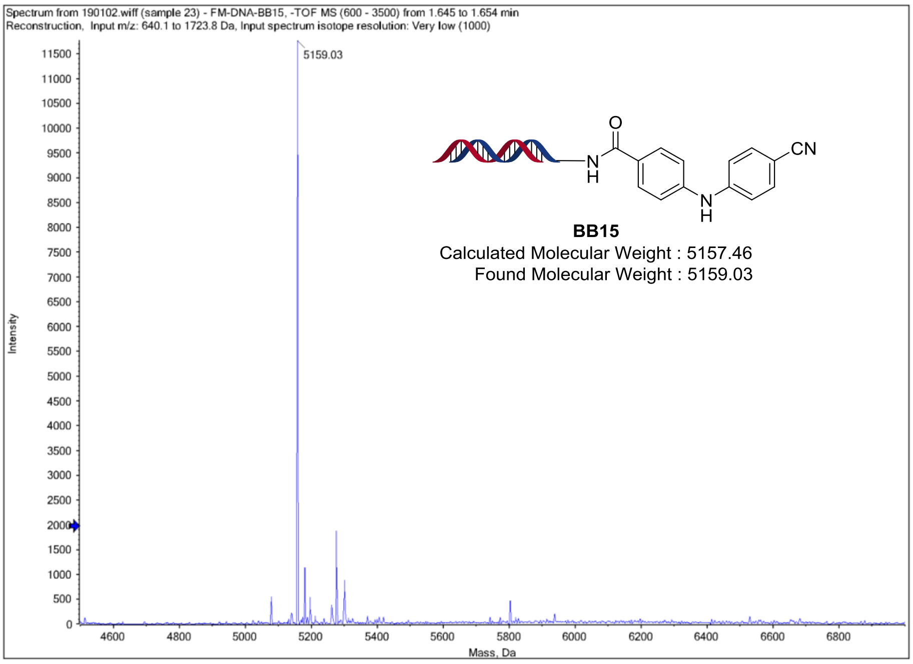
Spectrum from 190102.wiff (sample 23) - FM-DNA-BB15, -TOF MS (600 - 3500) from 1.645 to 1.654 min
Reconstruction, Input m/z: 640.1 to 1723.8 Da, Input spectrum isotope resolution: Very low (1000)
5159.03
Intensity
Mass, Da
CN
BB15
Calculated Molecular Weight : 5157.46
Found Molecular Weight : 5159.03

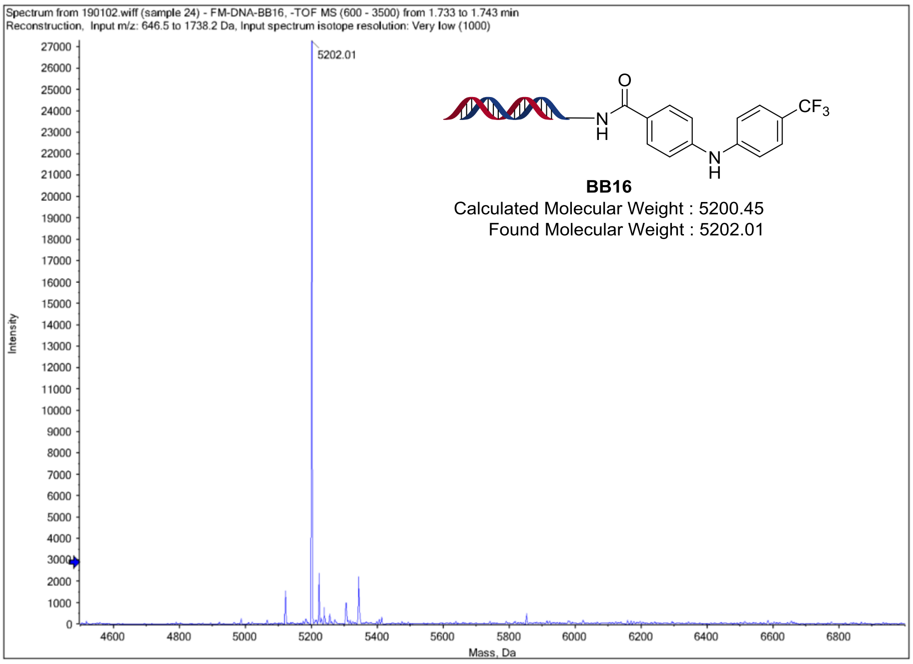
Spectrum from 190102.wiff (sample 24) - FM-DNA-BB16, -TOF MS (600 - 3500) from 1.733 to 1.743 min
Reconstruction, Input m/z: 646.5 to 1738.2 Da, Input spectrum isotope resolution: Very low (1000)
5202.01
BB16
Calculated Molecular Weight : 5200.45
Found Molecular Weight : 5202.01
Intensity
Mass, Da

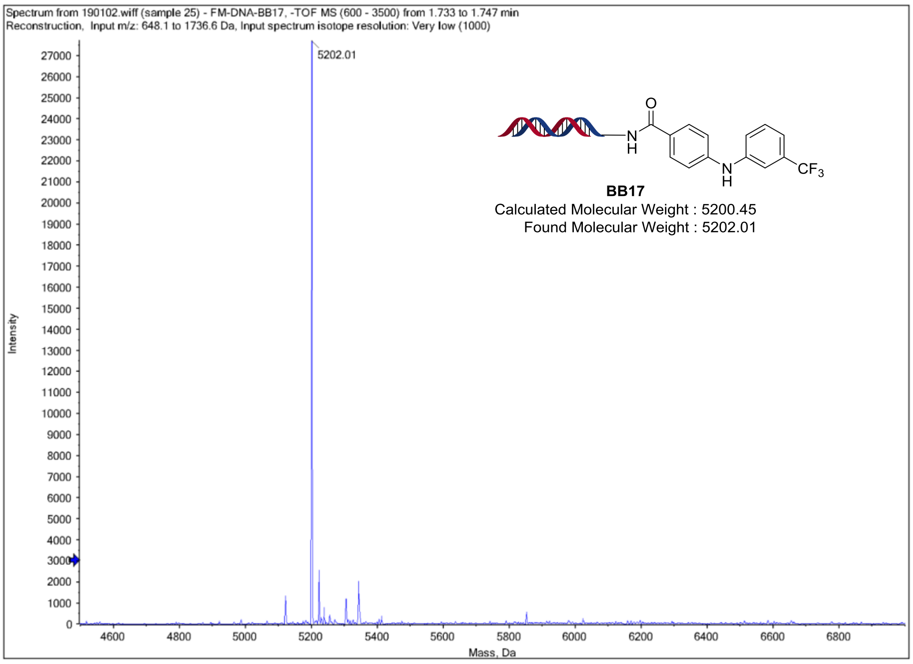
Spectrum from 190102.wiff (sample 25) - FM-DNA-BB17, -TOF MS (600 - 3500) from 1.733 to 1.747 min
Reconstruction, Input m/z: 648.1 to 1736.6 Da, Input spectrum isotope resolution: Very low (1000)
5202.01
BB17
Calculated Molecular Weight : 5200.45
Found Molecular Weight : 5202.01
Intensity
Mass, Da

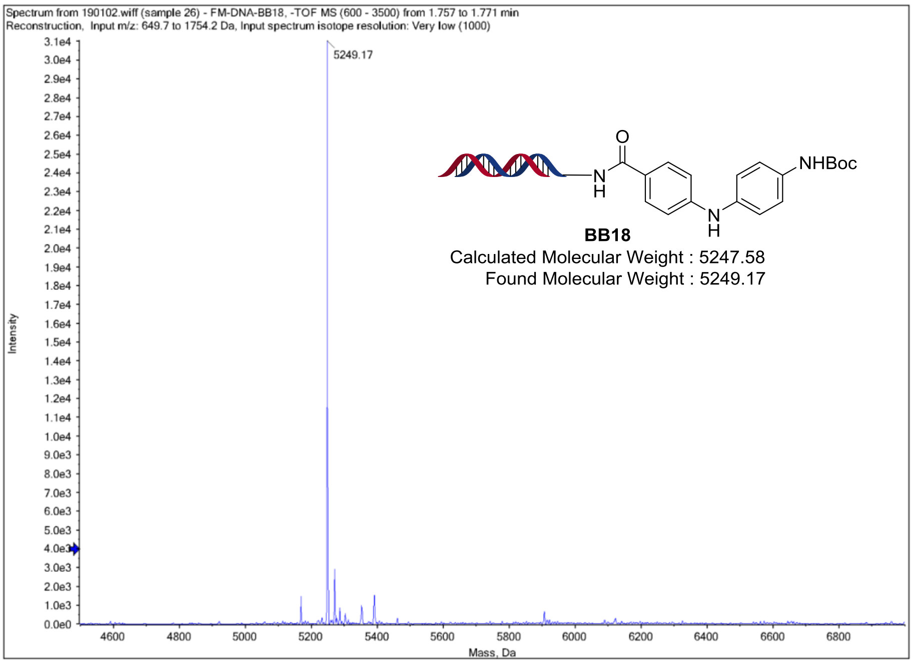
Spectrum from 190102.wiff (sample 26) - FM-DNA-BB18, -TOF MS (600 - 3500) from 1.757 to 1.771 min
Reconstruction, Input m/z: 649.7 to 1754.2 Da, Input spectrum isotope resolution: Very low (1000)
5249.17
Intensity
Mass, Da
NHBoc
BB18
Calculated Molecular Weight : 5247.58
Found Molecular Weight : 5249.17

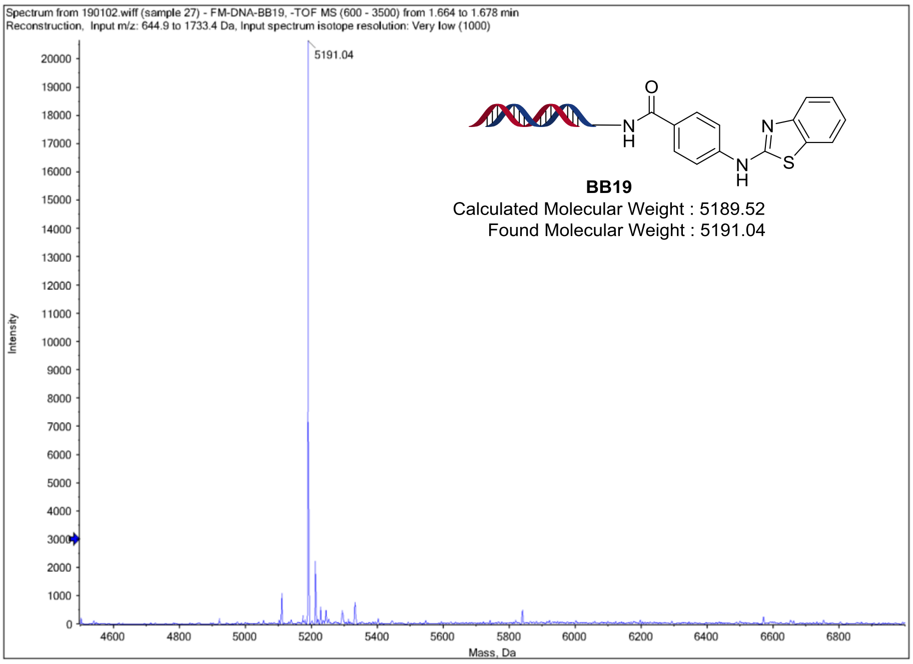
Spectrum from 190102.wiff (sample 27) - FM-DNA-BB19, -TOF MS (600 - 3500) from 1.664 to 1.678 min
Reconstruction, Input m/z: 644.9 to 1733.4 Da, Input spectrum isotope resolution: Very low (1000)
5191.04
Intensity
Mass, Da
BB19
Calculated Molecular Weight : 5189.52
Found Molecular Weight : 5191.04

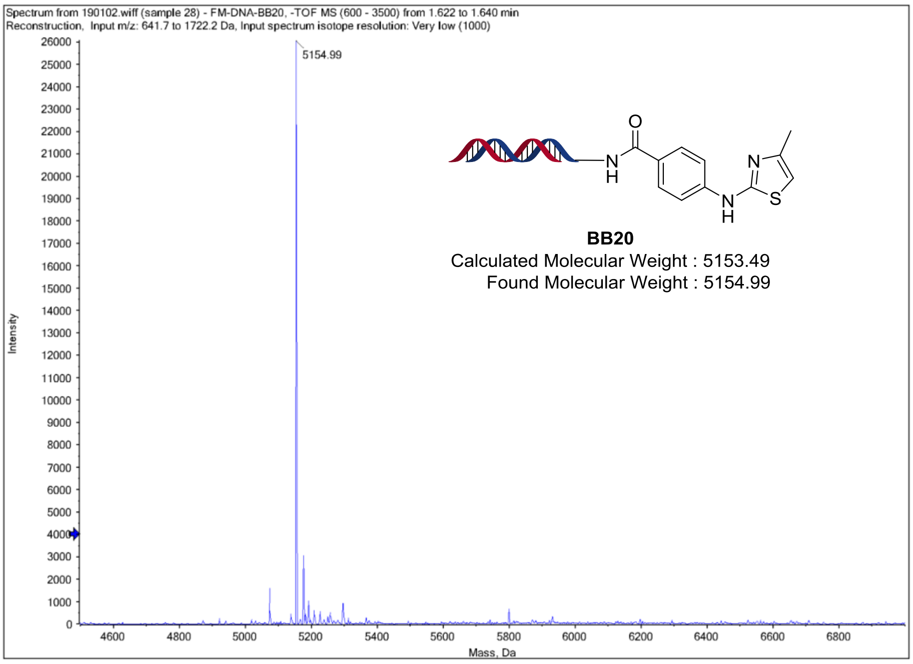

Spectrum from 190102.wiff (sample 28) - FM-DNA-BB20, -TOF MS (600 - 3500) from 1.622 to 1.640 min
Reconstruction, Input m/z: 641.7 to 1722.2 Da, Input spectrum isotope resolution: Very low (1000)
5154.99
Intensity
Mass, Da
BB20
Calculated Molecular Weight : 5153.49
Found Molecular Weight : 5154.99

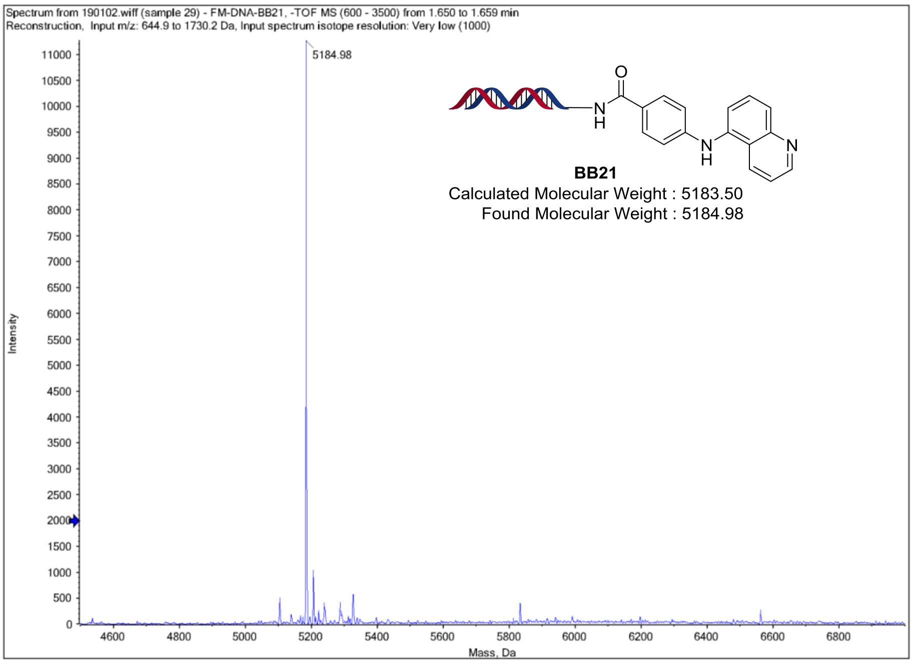

Spectrum from 190102.wiff (sample 29) - FM-DNA-BB21, -TOF MS (600 - 3500) from 1.650 to 1.659 min
Reconstruction, Input m/z: 644.9 to 1730.2 Da, Input spectrum isotope resolution: Very low (1000)
5184.98
Intensity
Mass, Da
BB21
Calculated Molecular Weight : 5183.50
Found Molecular Weight : 5184.98

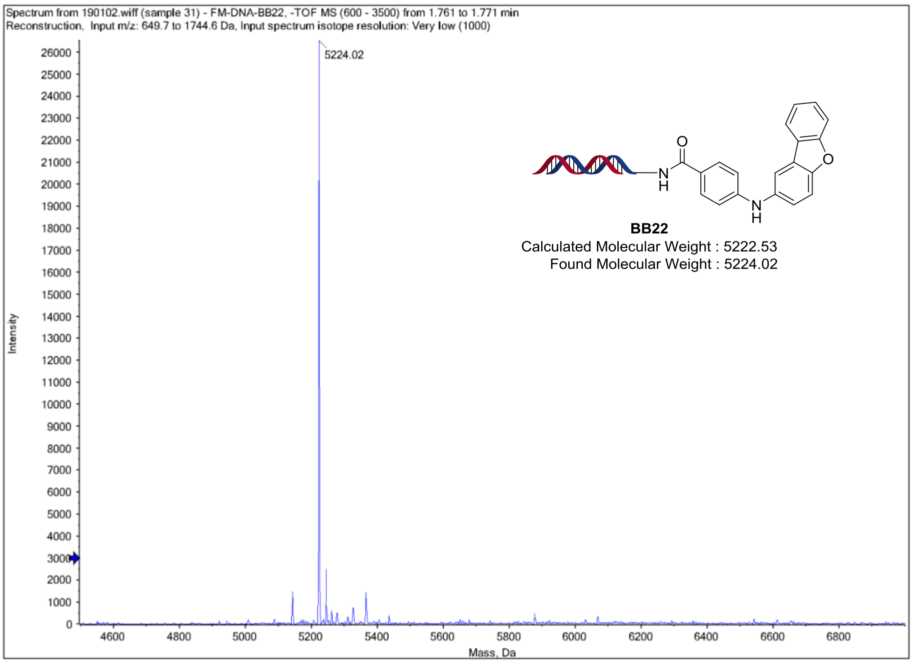
Spectrum from 190102.wiff (sample 31) - FM-DNA-BB22, -TOF MS (600 - 3500) from 1.761 to 1.771 min
Reconstruction, Input m/z: 649.7 to 1744.6 Da, Input spectrum isotope resolution: Very low (1000)
5224.02
Intensity
Mass, Da
BB22
Calculated Molecular Weight : 5222.53
Found Molecular Weight : 5224.02

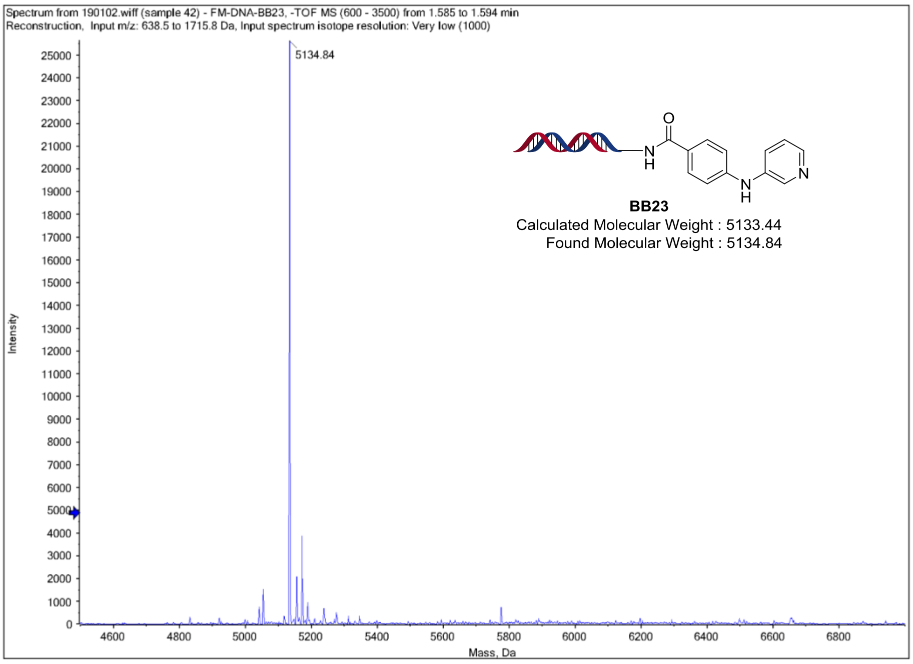
Spectrum from 190102.wiff (sample 42) - FM-DNA-BB23, -TOF MS (600 - 3500) from 1.585 to 1.594 min
Reconstruction, Input m/z: 638.5 to 1715.8 Da, Input spectrum isotope resolution: Very low (1000)
5134.84
Intensity
Mass, Da
BB23
Calculated Molecular Weight : 5133.44
Found Molecular Weight : 5134.84

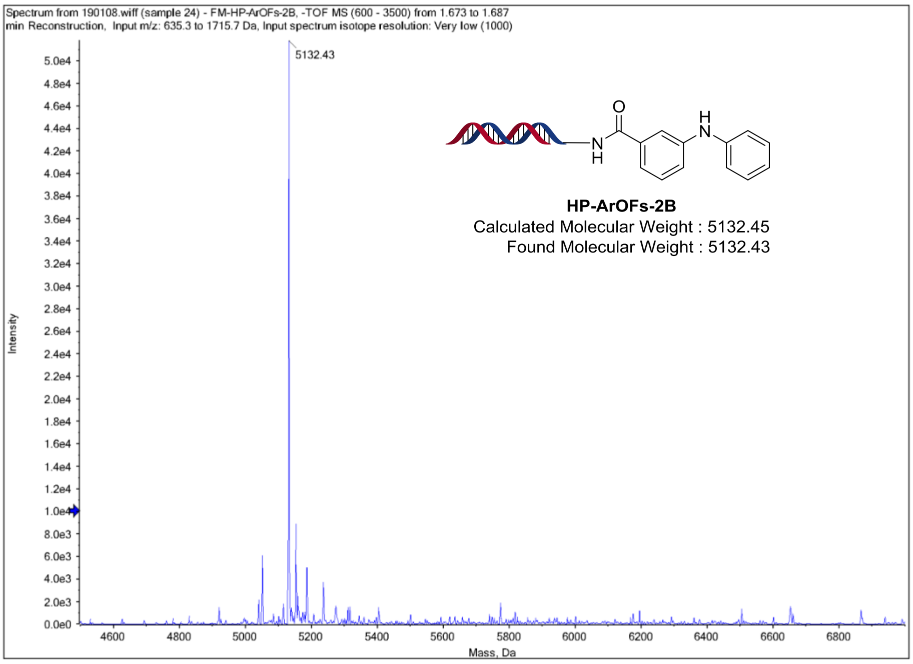
Spectrum from 190108.wiff (sample 24) - FM-HP-ArOFs-2B, -TOF MS (600 - 3500) from 1.673 to 1.687
min Reconstruction, Input m/z: 635.3 to 1715.7 Da, Input spectrum isotope resolution: Very low (1000)
5132.43
HP-ArOFs-2B
Calculated Molecular Weight : 5132.45
Found Molecular Weight : 5132.43
Intensity
Mass, Da

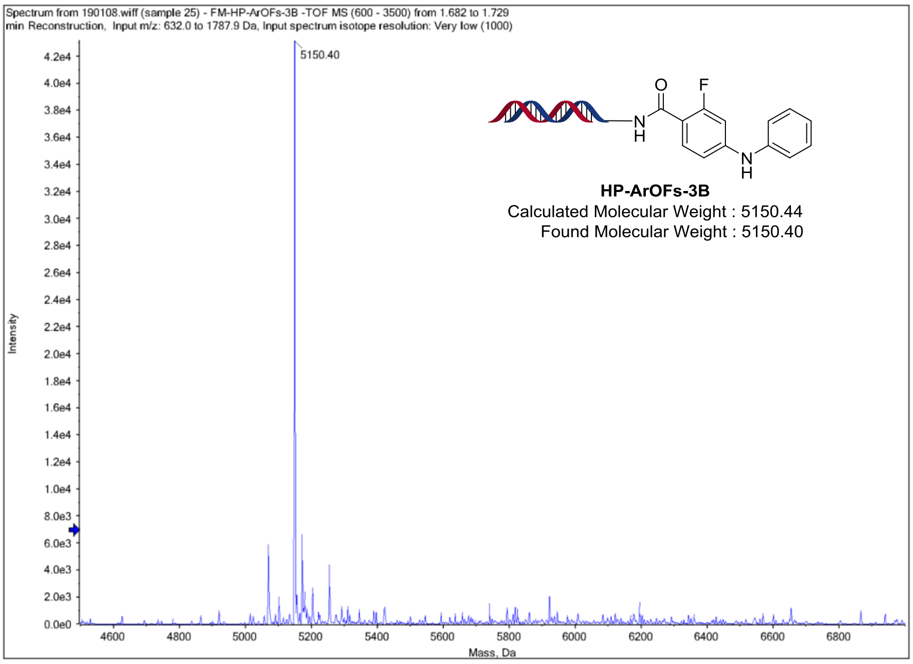
Spectrum from 190108.wiff (sample 25) - FM-HP-ArOFs-3B -TOF MS (600 - 3500) from 1.682 to 1.729 min Reconstruction, Input m/z: 632.0 to 1787.9 Da, Input spectrum isotope resolution: Very low (1000)
5150.40
HP-ArOFs-3B
Calculated Molecular Weight : 5150.44
Found Molecular Weight : 5150.40
Intensity
Mass, Da

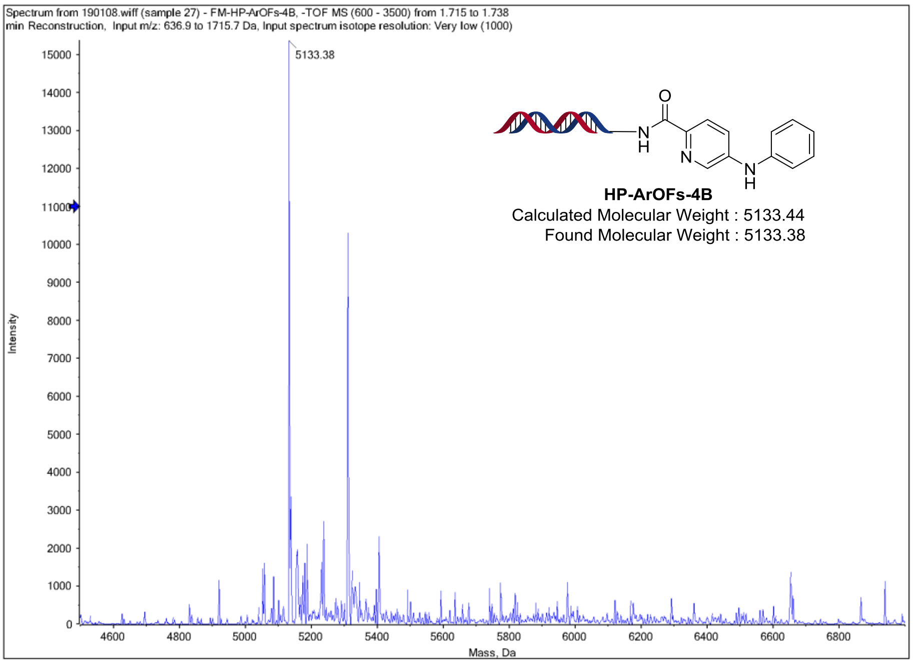
Spectrum from 190108.wiff (sample 27) - FM-HP-ArOFs-4B, -TOF MS (600 - 3500) from 1.715 to 1.738 min Reconstruction, Input m/z: 636.9 to 1715.7 Da, Input spectrum isotope resolution: Very low (1000)
5133.38
HP-ArOFs-4B
Calculated Molecular Weight : 5133.44
Found Molecular Weight : 5133.38
Intensity
Mass, Da

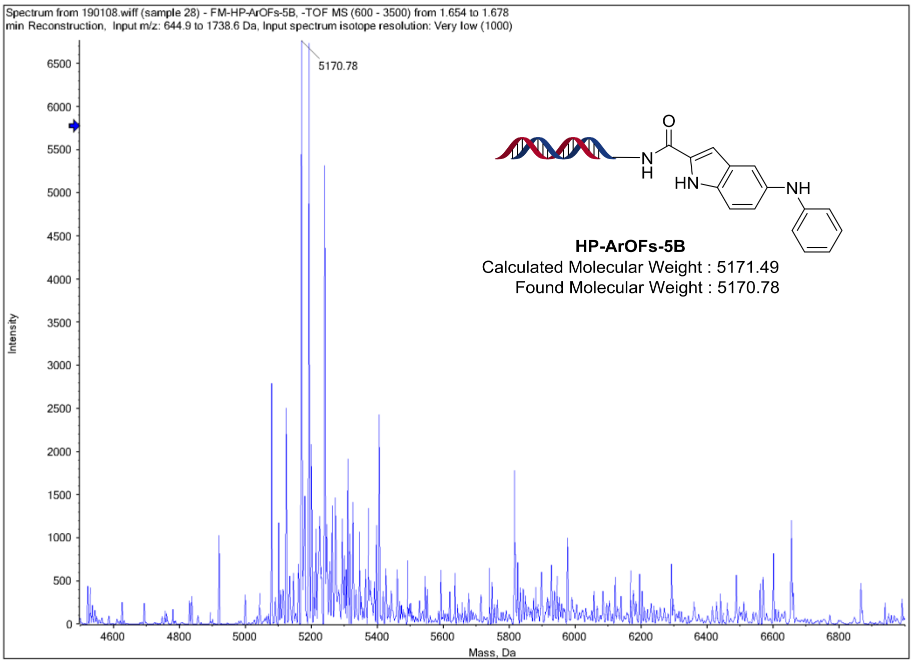
Spectrum from 190108.wiff (sample 28) - FM-HP-ArOFs-5B, -TOF MS (600 - 3500) from 1.654 to 1.678 min Reconstruction, Input m/z: 644.9 to 1738.6 Da, Input spectrum isotope resolution: Very low (1000)
5170.78
HP-ArOFs-5B
Calculated Molecular Weight : 5171.49
Found Molecular Weight : 5170.78
Intensity
Mass, Da

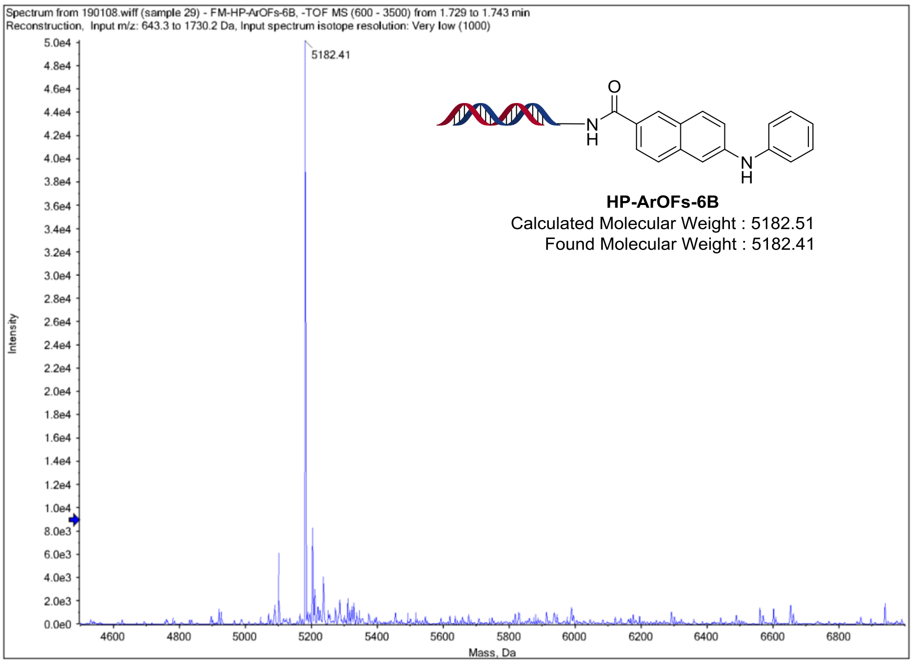
Spectrum from 190108.wiff (sample 29) - FM-HP-ArOFs-6B, -TOF MS (600 - 3500) from 1.729 to 1.743 min Reconstruction, Input m/z: 643.3 to 1730.2 Da, Input spectrum isotope resolution: Very low (1000)
5182.41
HP-ArOFs-6B
Calculated Molecular Weight : 5182.51
Found Molecular Weight : 5182.41
Intensity
Mass, Da

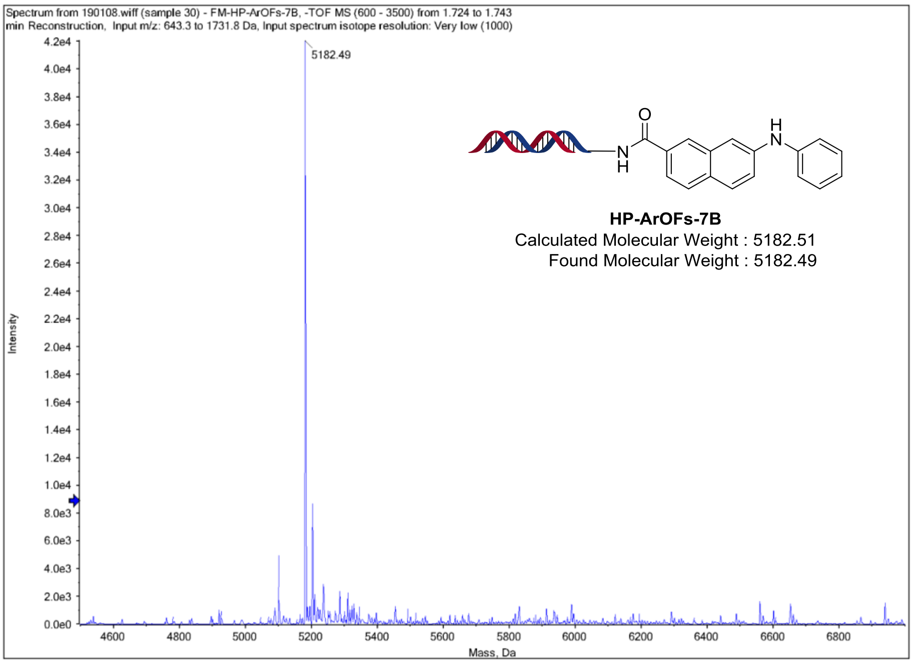
Spectrum from 190108.wiff (sample 30) - FM-HP-ArOFs-7B, -TOF MS (600 - 3500) from 1.724 to 1.743
min Reconstruction, Input m/z: 643.3 to 1731.8 Da, Input spectrum isotope resolution: Very low (1000)
5182.49
HP-ArOFs-7B
Calculated Molecular Weight : 5182.51
Found Molecular Weight : 5182.49
Intensity
Mass, Da

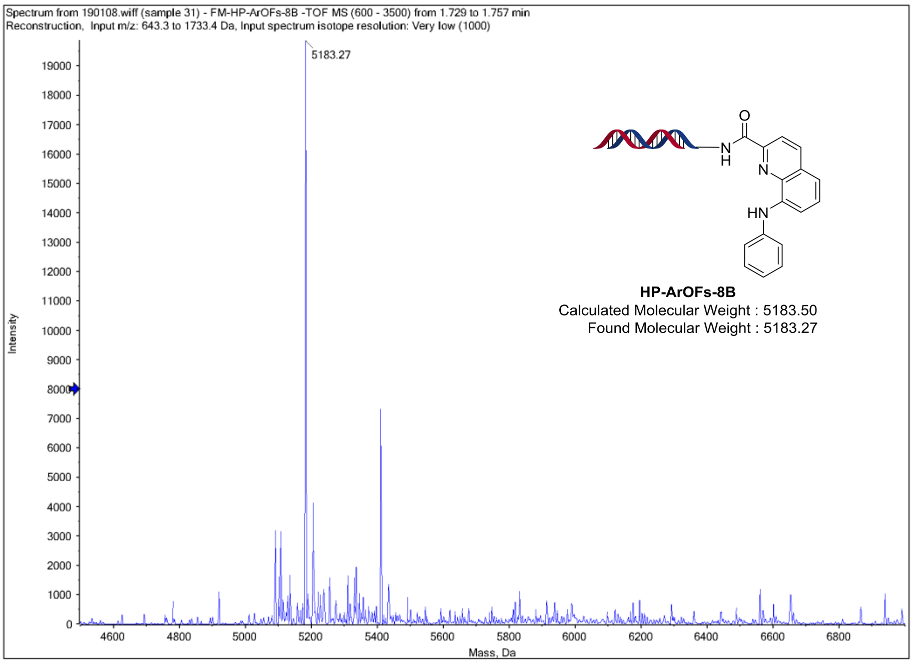
Spectrum from 190108.wiff (sample 31) - FM-HP-ArOFs-8B -TOF MS (600 - 3500) from 1.729 to 1.757 min
Reconstruction, Input m/z: 643.3 to 1733.4 Da, Input spectrum isotope resolution: Very low (1000)
5183.27
Intensity
Mass, Da
HP-ArOFs-8B
Calculated Molecular Weight : 5183.50
Found Molecular Weight : 5183.27

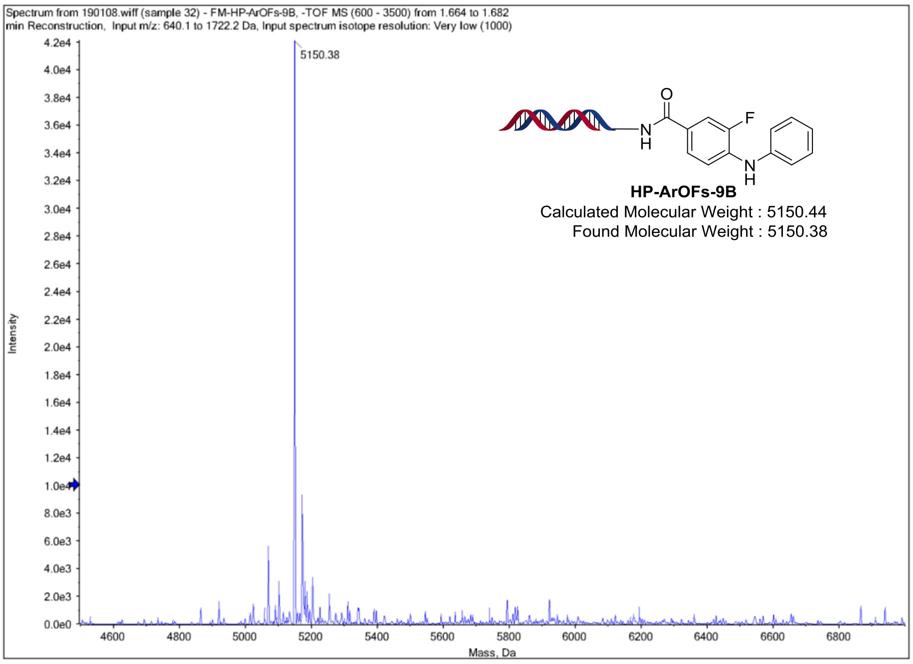
Spectrum from 190108.wiff (sample 32) - FM-HP-ArOFs-9B, -TOF MS (600 - 3500) from 1.664 to 1.682
min Reconstruction, Input m/z: 640.1 to 1722.2 Da, Input spectrum isotope resolution: Very low (1000)
5150.38
Intensity
Mass, Da
HP-ArOFs-9B
Calculated Molecular Weight : 5150.44
Found Molecular Weight : 5150.38
